# Supplementary material for: The SKBR3 cell-membrane proteome reveals telltales of aberrant cancer cell proliferation and targets for precision medicine applications
Source: Sci Rep. 2022 Jun 27;12:10847. doi: 10.1038/s41598-022-14418-0 (PMC9237123; doi:10.1038/s41598-022-14418-0)

The SKBR3 Cell-Membrane Proteome Reveals Telltales of Aberrant Cancer Cell Proliferation and Targets for Precision Medicine Applications

Arba Karcini and Iulia M. Lazar*

Virginia Tech, Blacksburg, VA

GPCR/PRM validations

Adhesion G protein-coupled receptor L1 (ADGRL1)

GSIYAGDVSSSVK, Charge 2, m/z = 635.3193

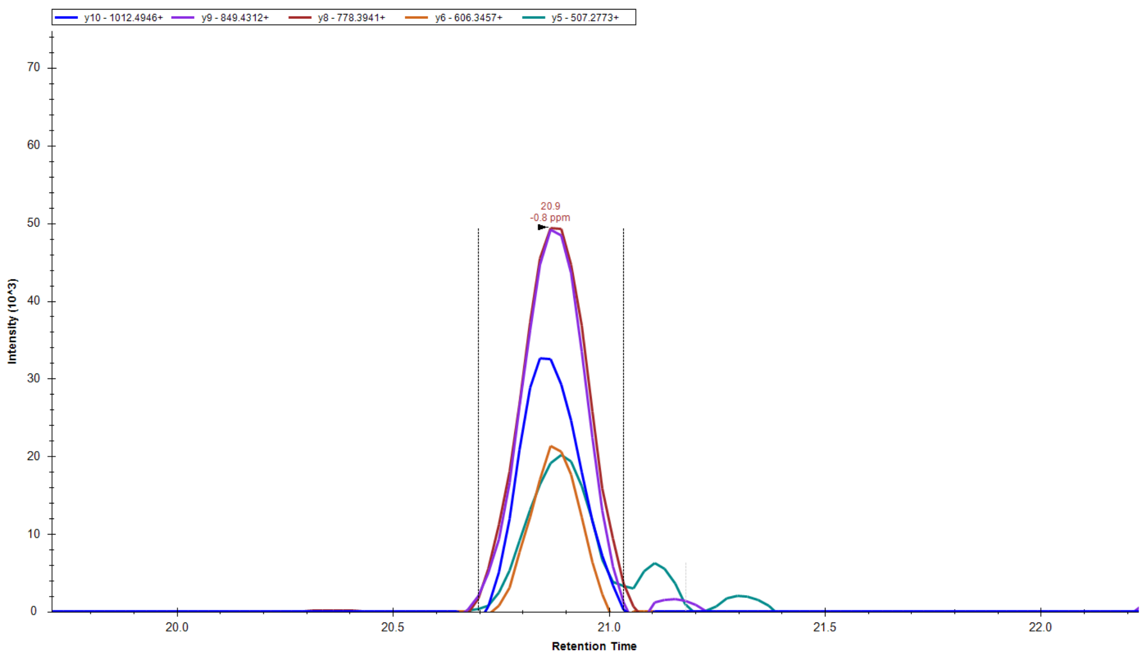

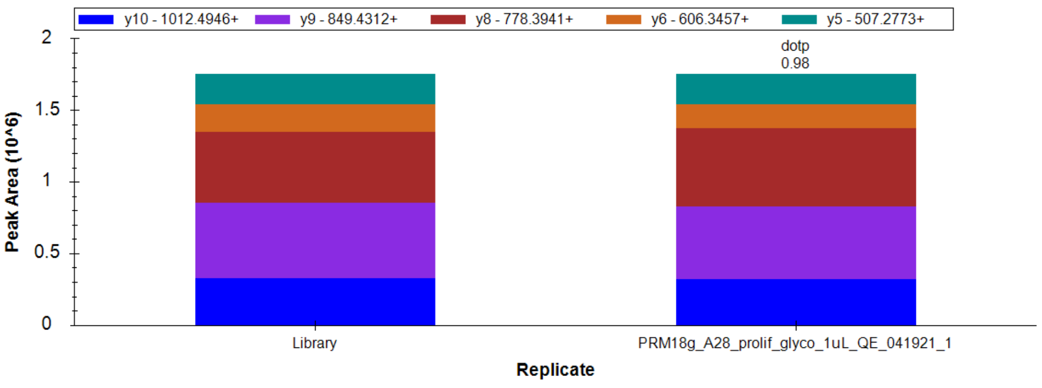

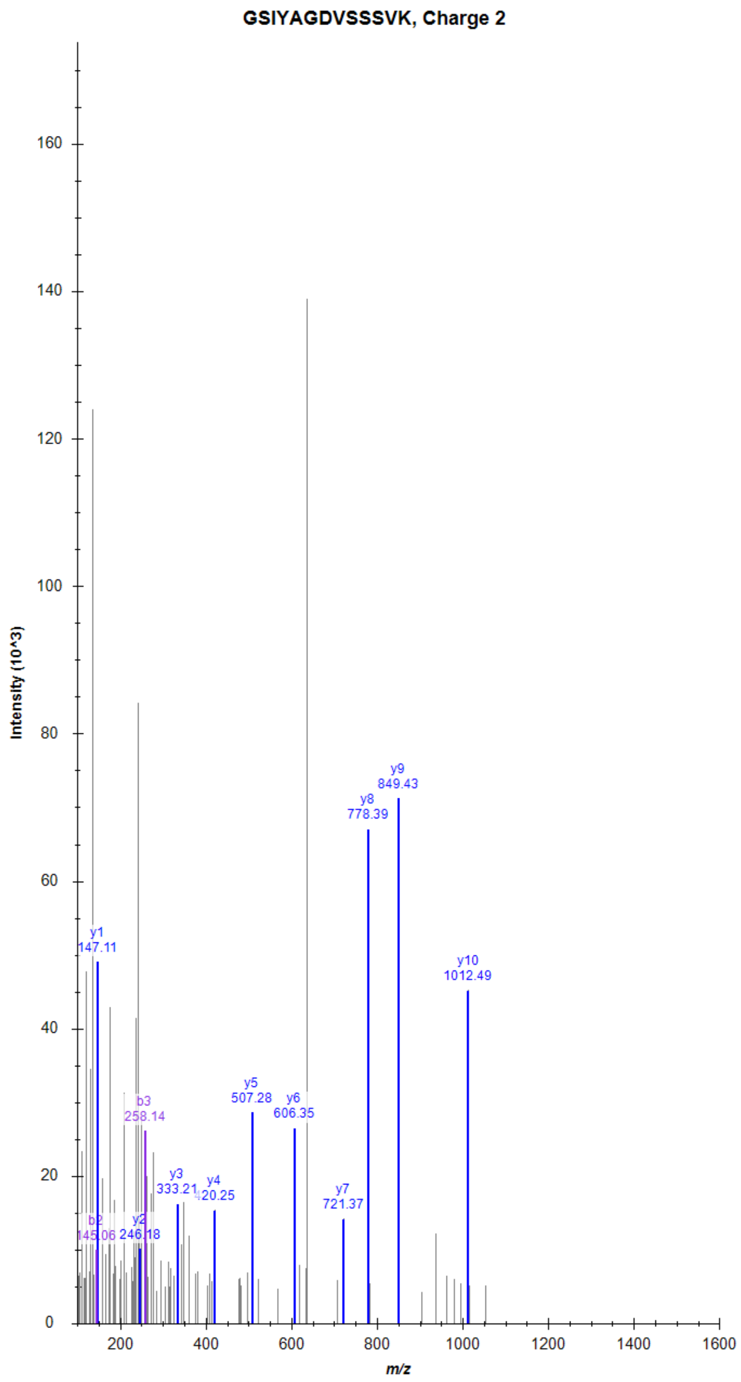

Adhesion G protein-coupled receptor L1 (ADGRL1)

SGETVINTANYHDTSPYR, Charge 3, m/z = 675.6484

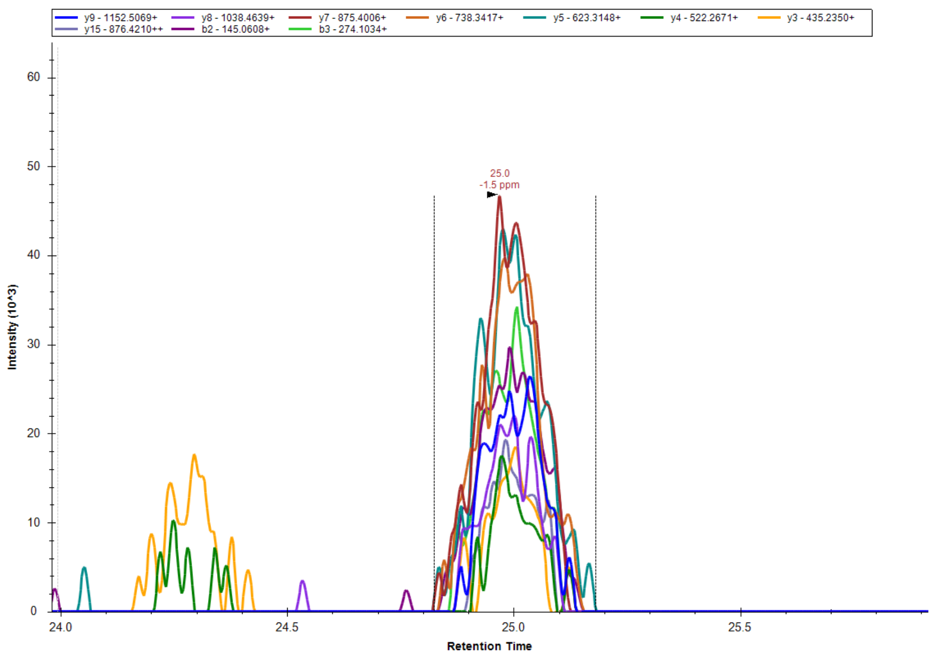

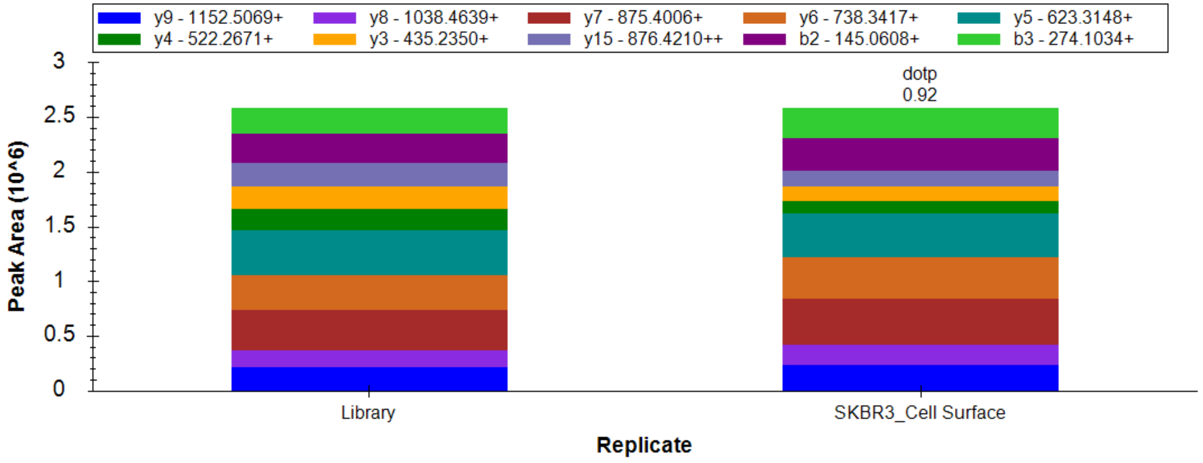

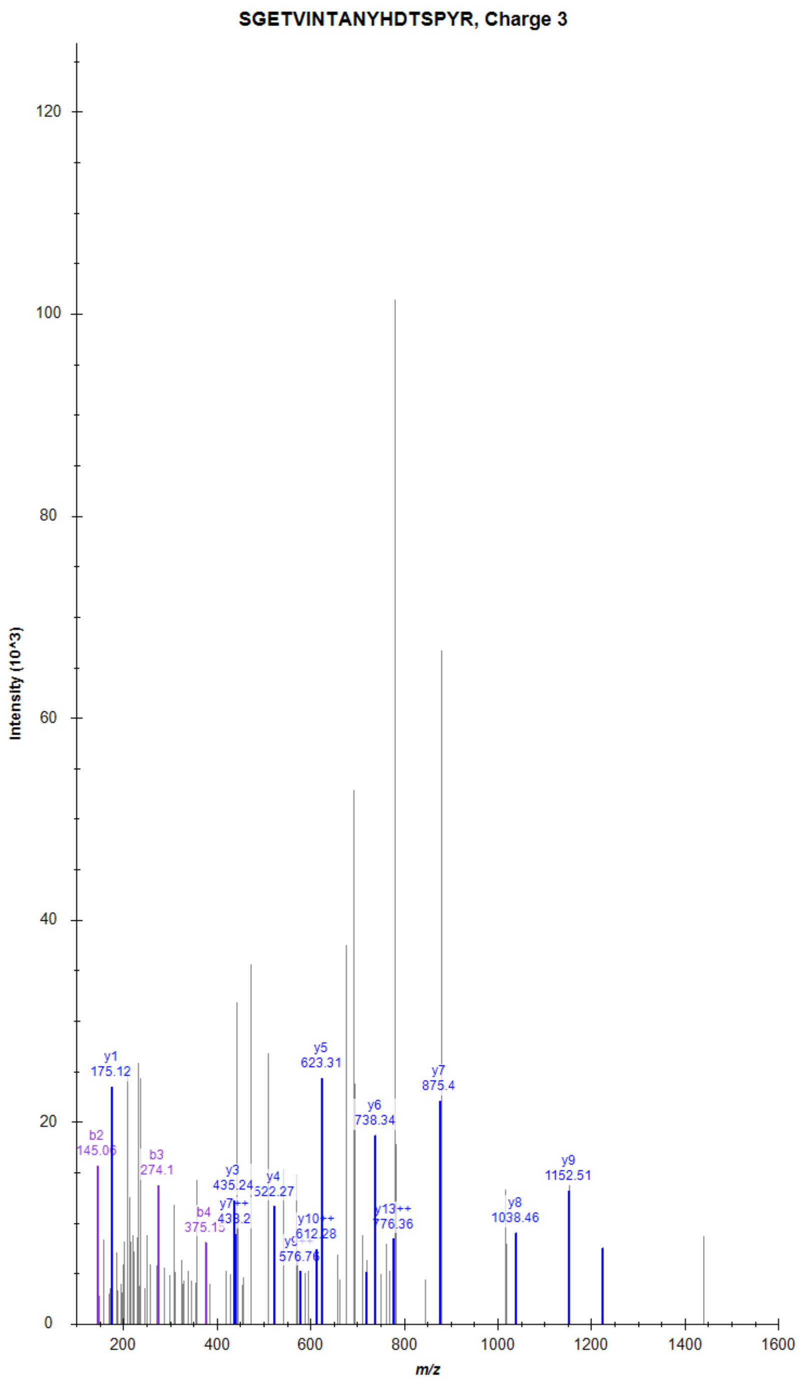

Adhesion G protein-coupled receptor L2 (ADGRL2)

AALPFGLVR, Charge 2, m/z = 472.2903

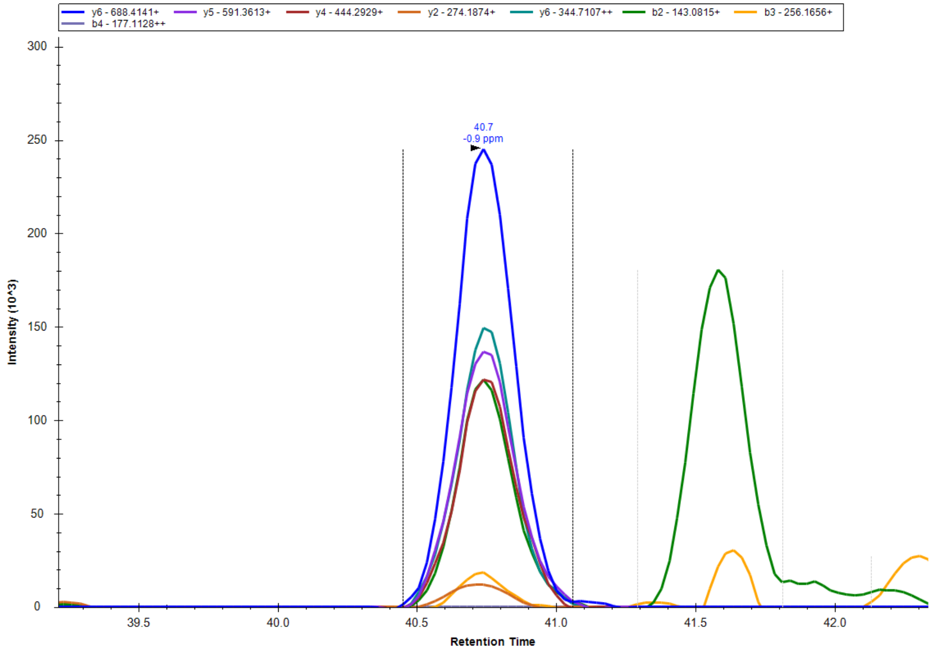

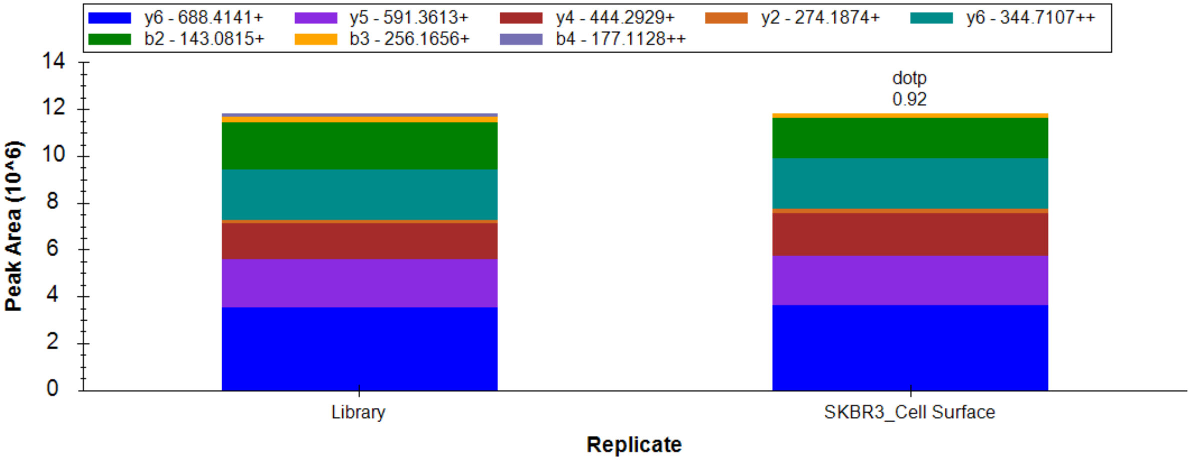

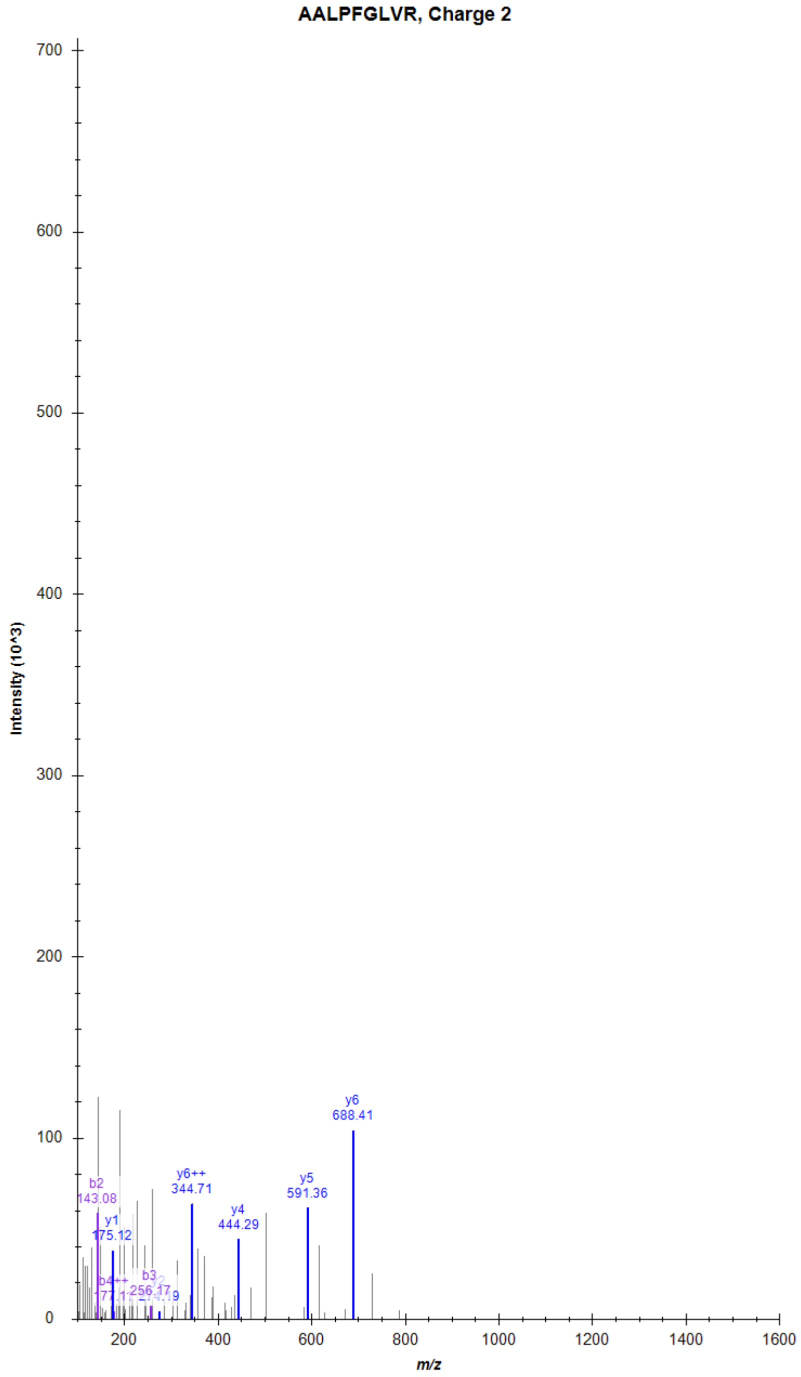

Adhesion G protein-coupled receptor L2 (ADGRL2)

SGEAIINYANYHDTSPYR, Charge 3, m/z = 690.9888

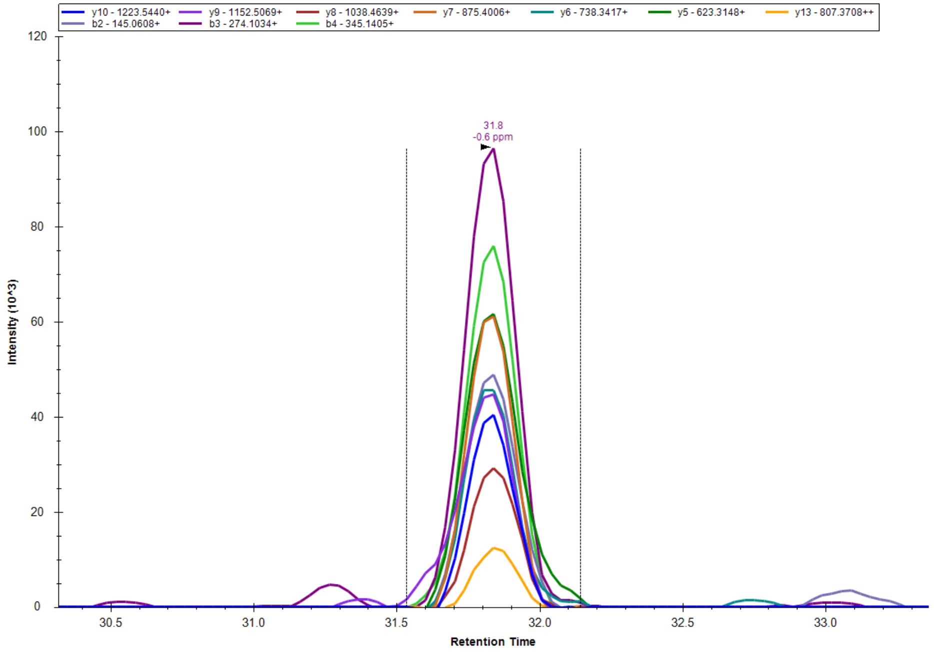

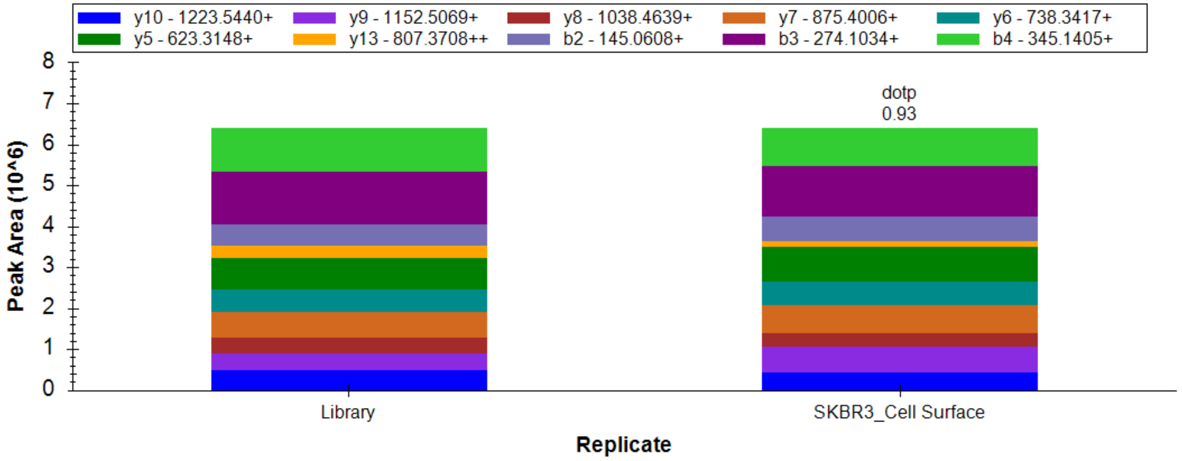

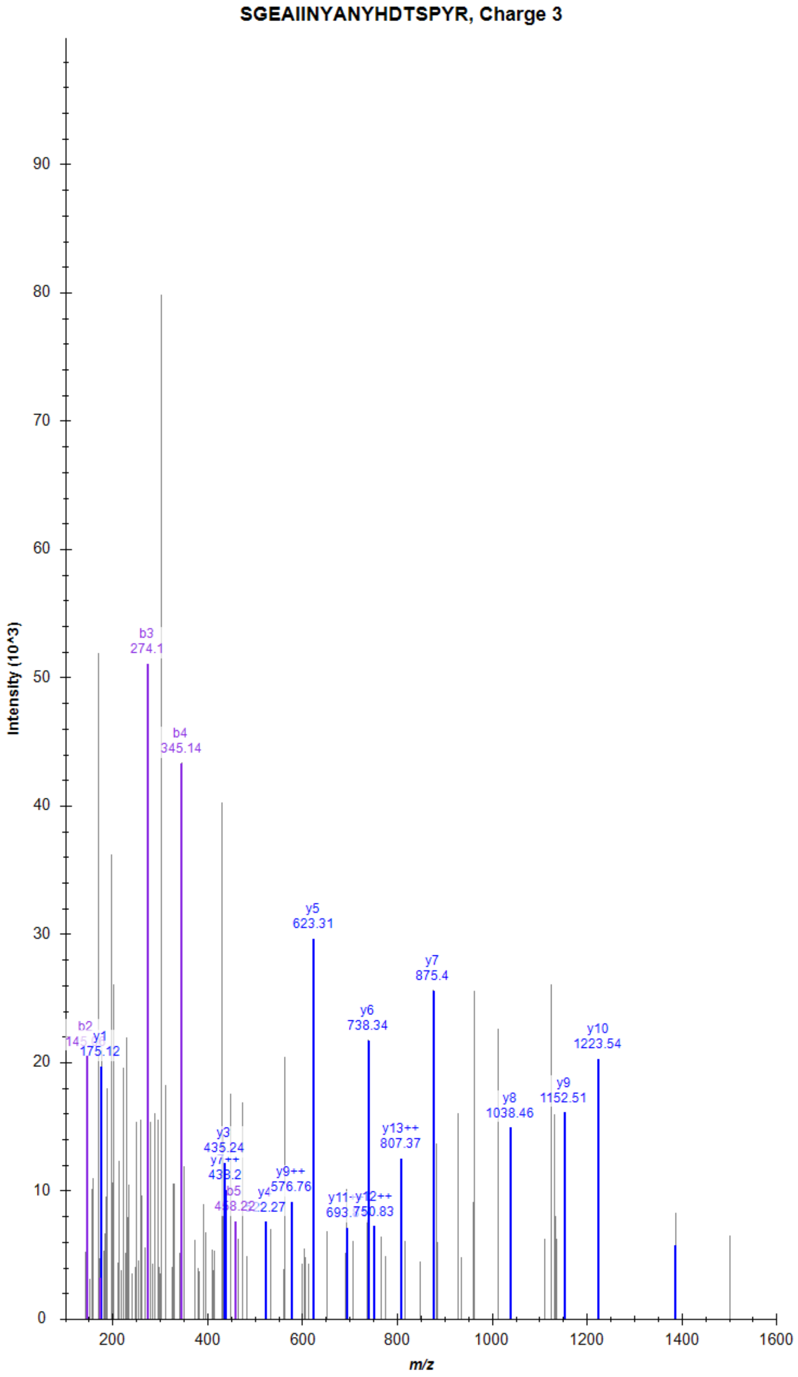

Vasoactive intestinal polypeptide receptor 1 (VIPR1)

AASLDEQQTMFYGSVK, Charge 2, m/z = 887.9209

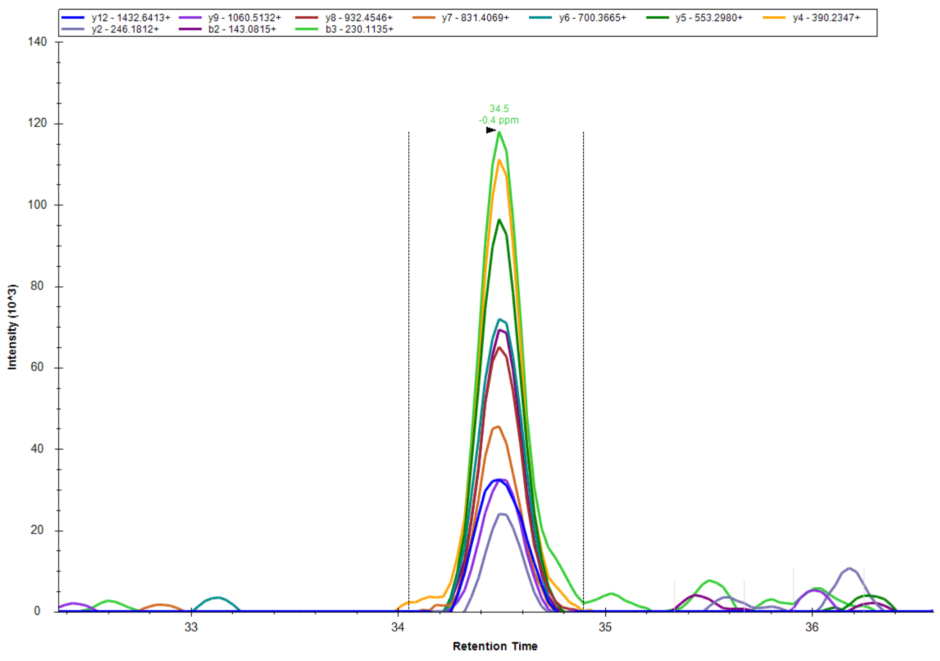

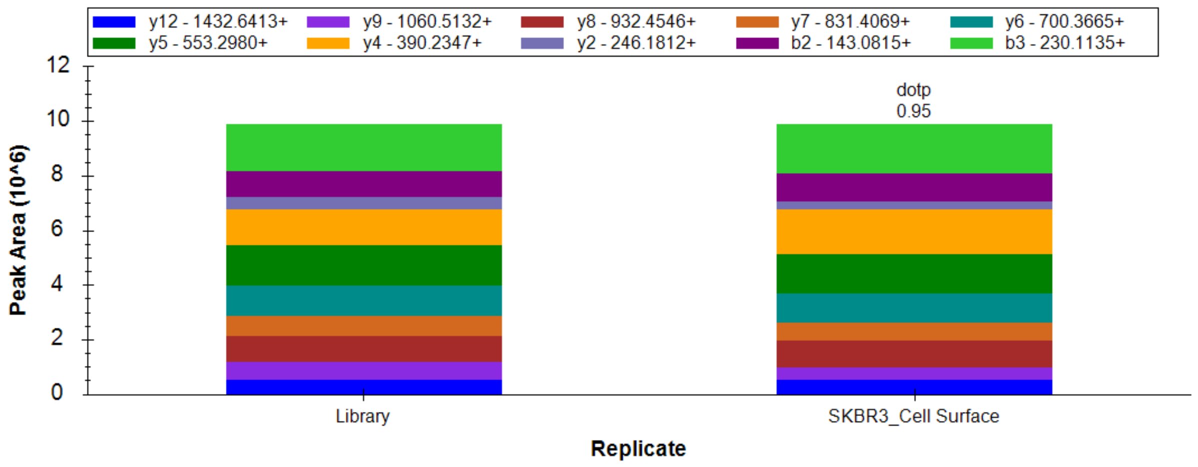

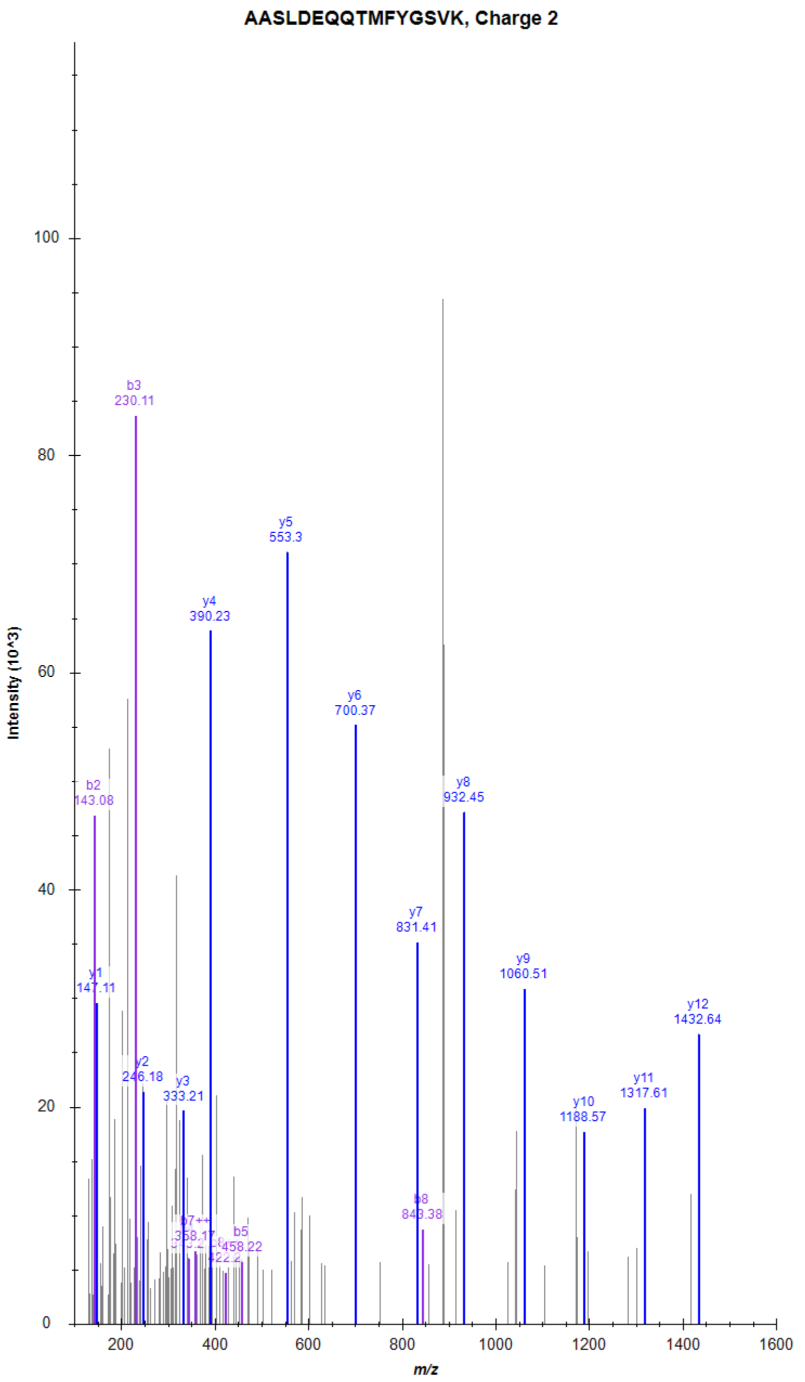

Vasoactive intestinal polypeptide receptor 1 (VIPR1)

LFSSIQGR, Charge 2, m/z = 454.2533

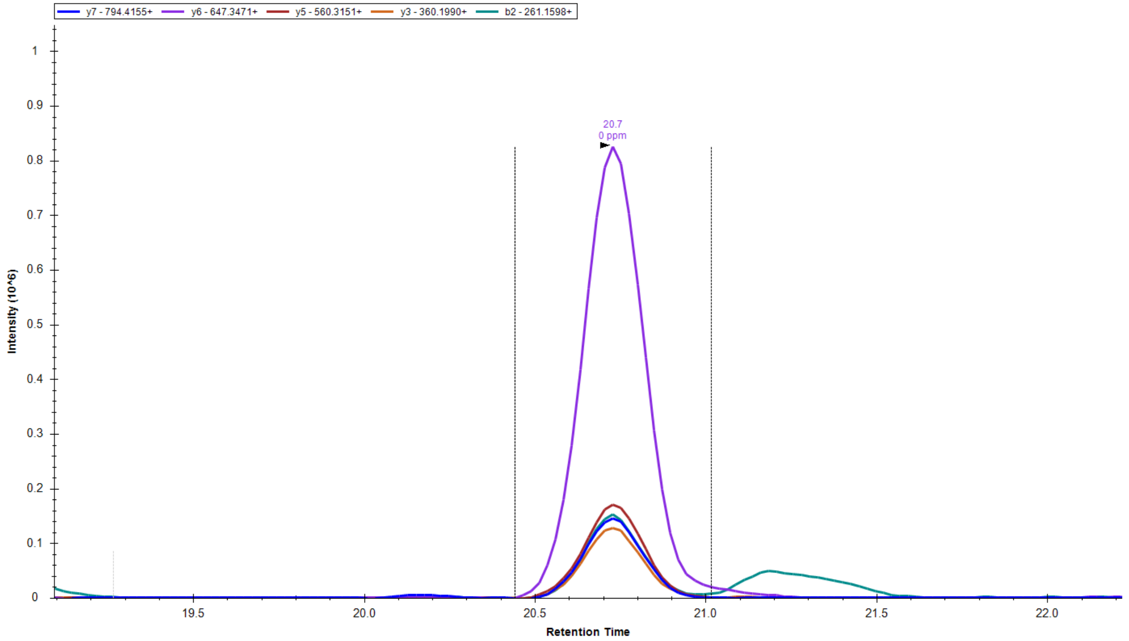

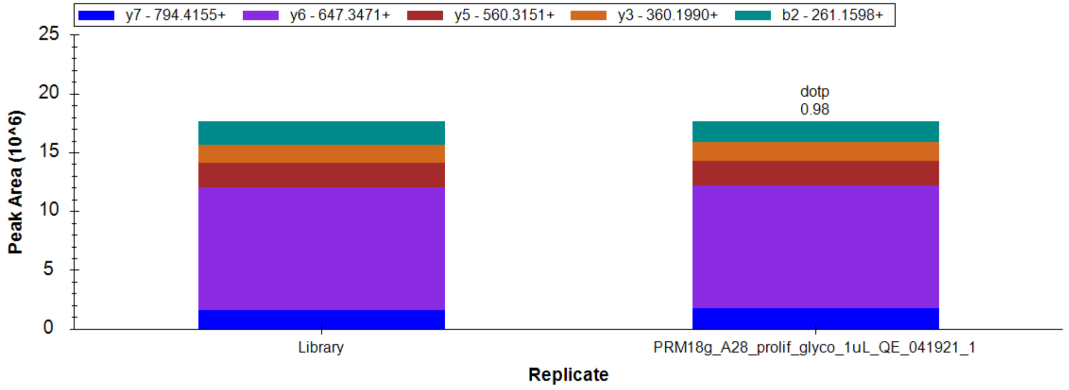

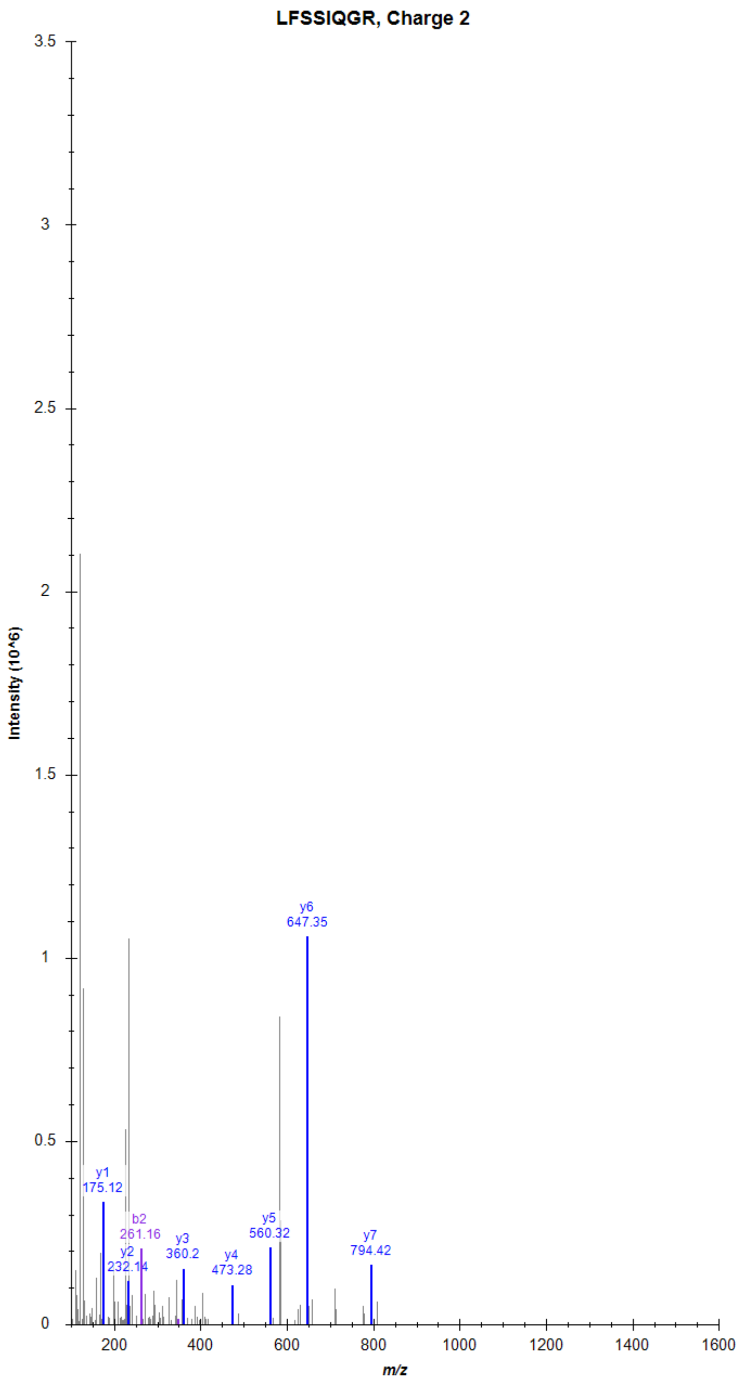

CD97 antigen (CD97)

TSSAEVTIQNVIK, Charge 2, m/z = 695.3828

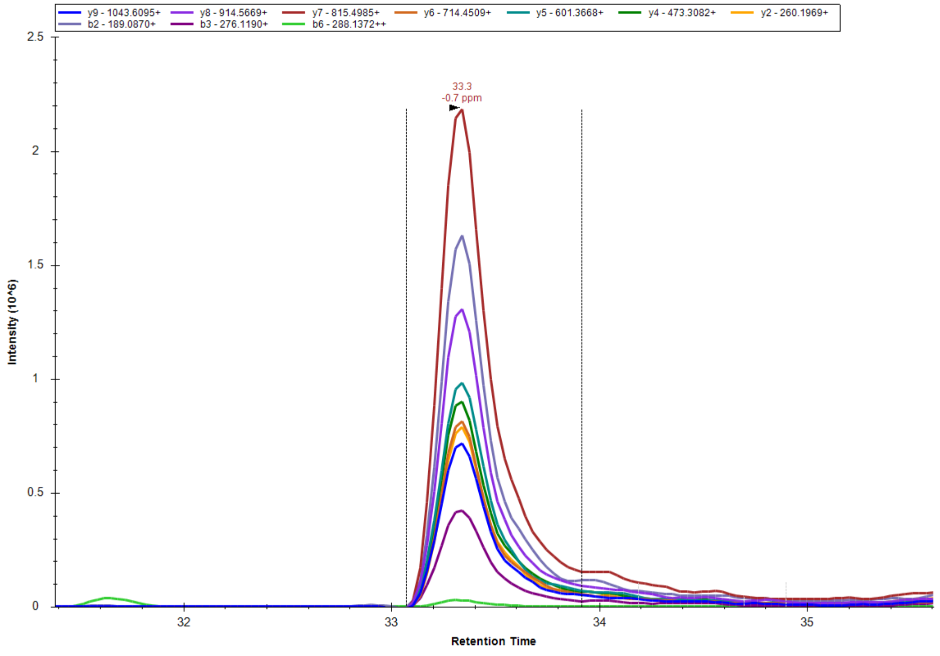

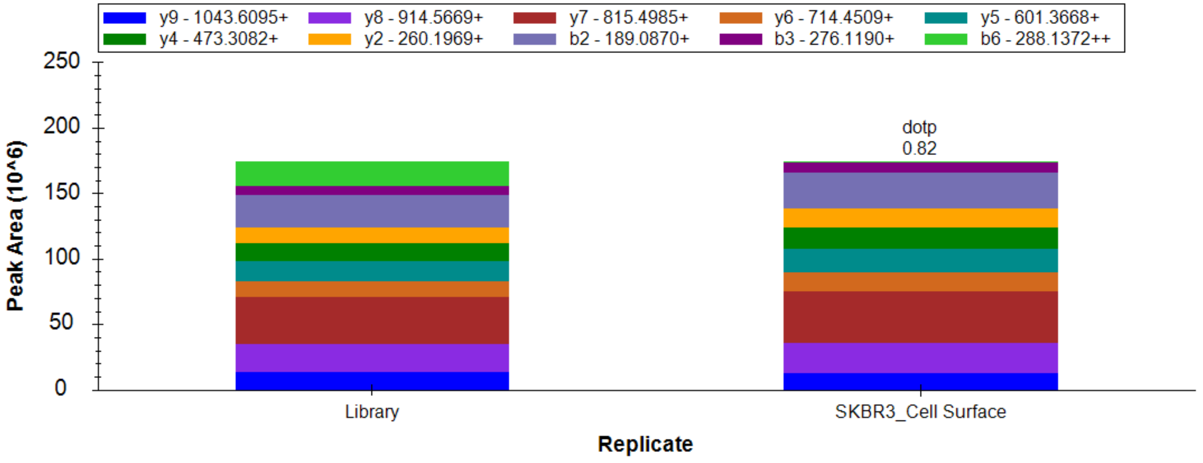

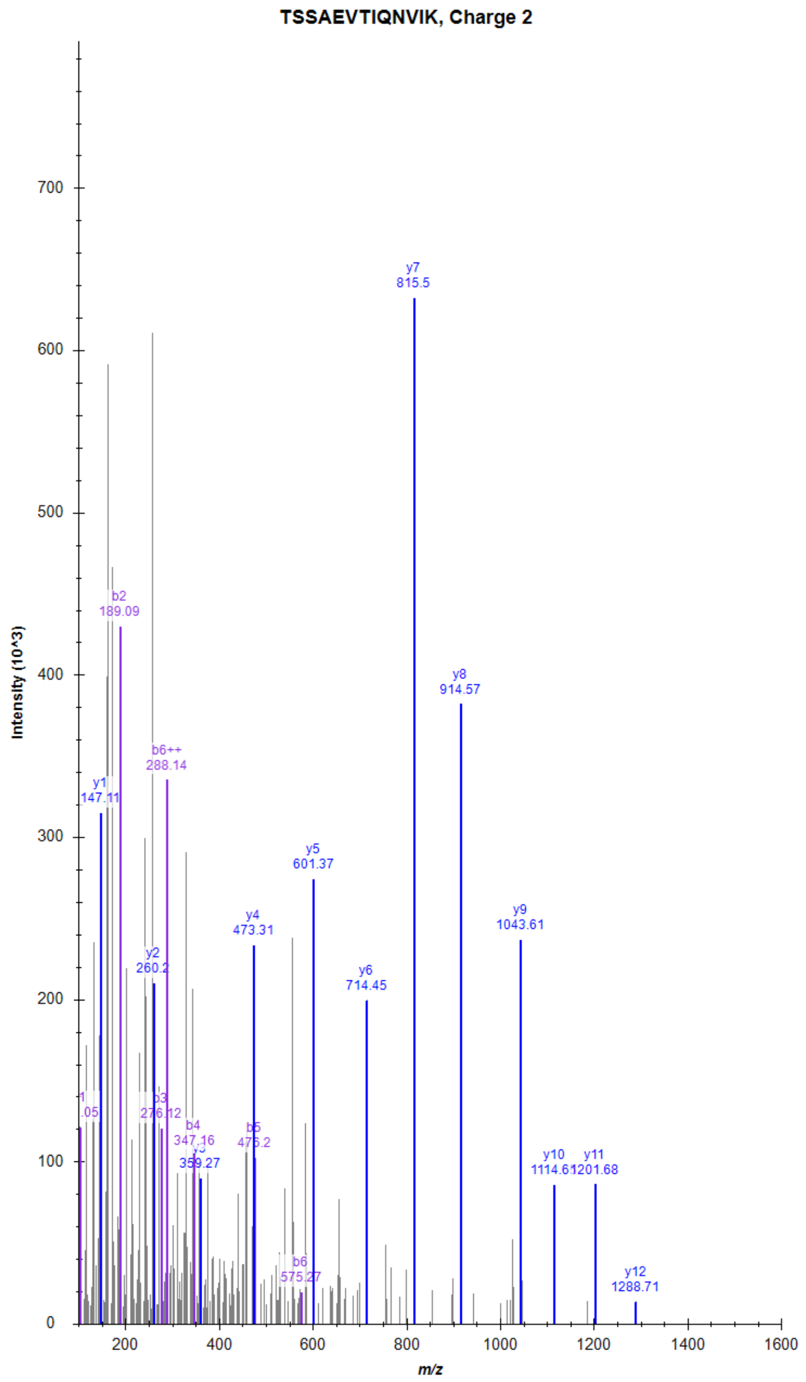

CD97 antigen (CD97)

KQAELEEIYESSIR, Charge 2, m/z = 847.9334

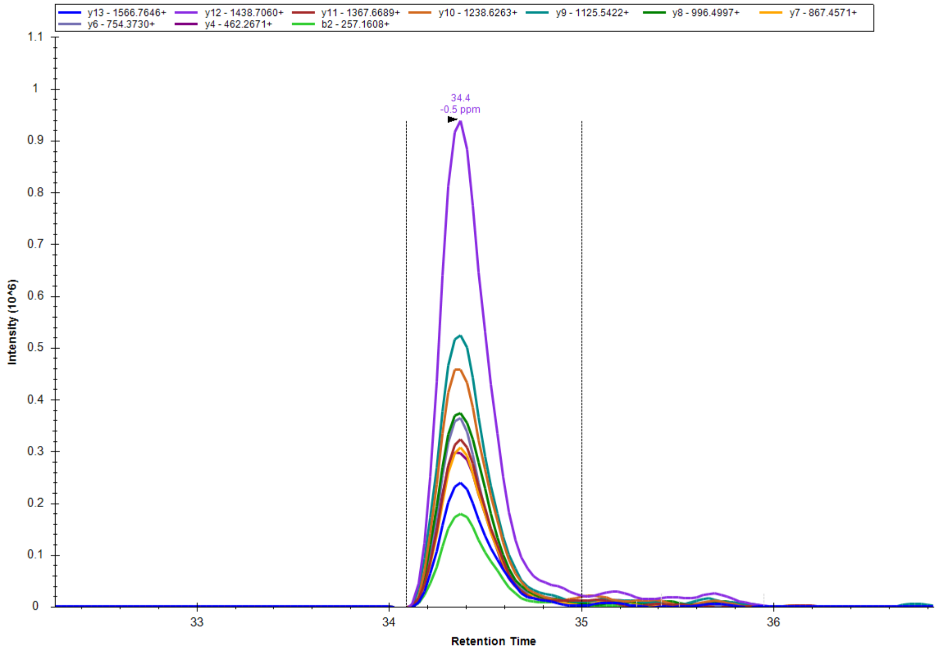

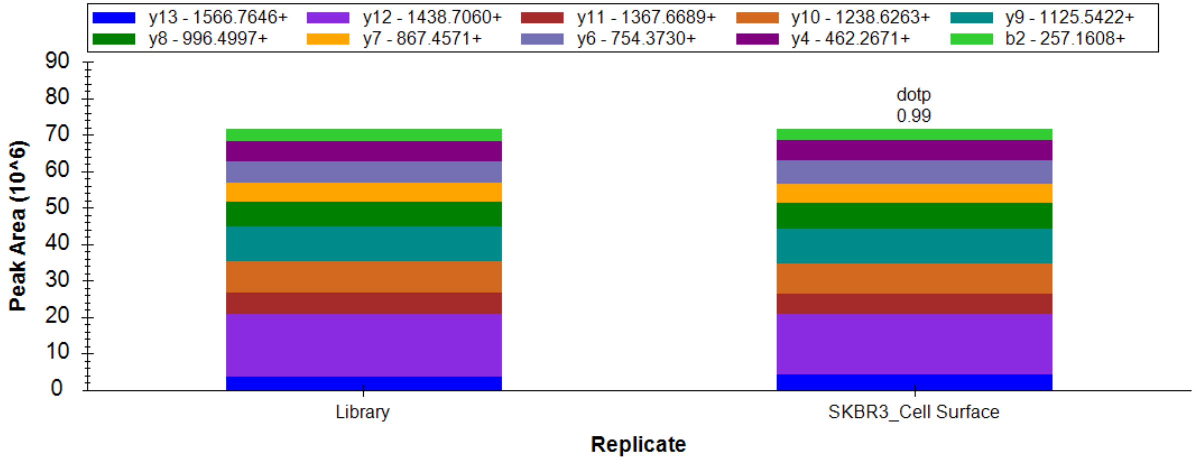

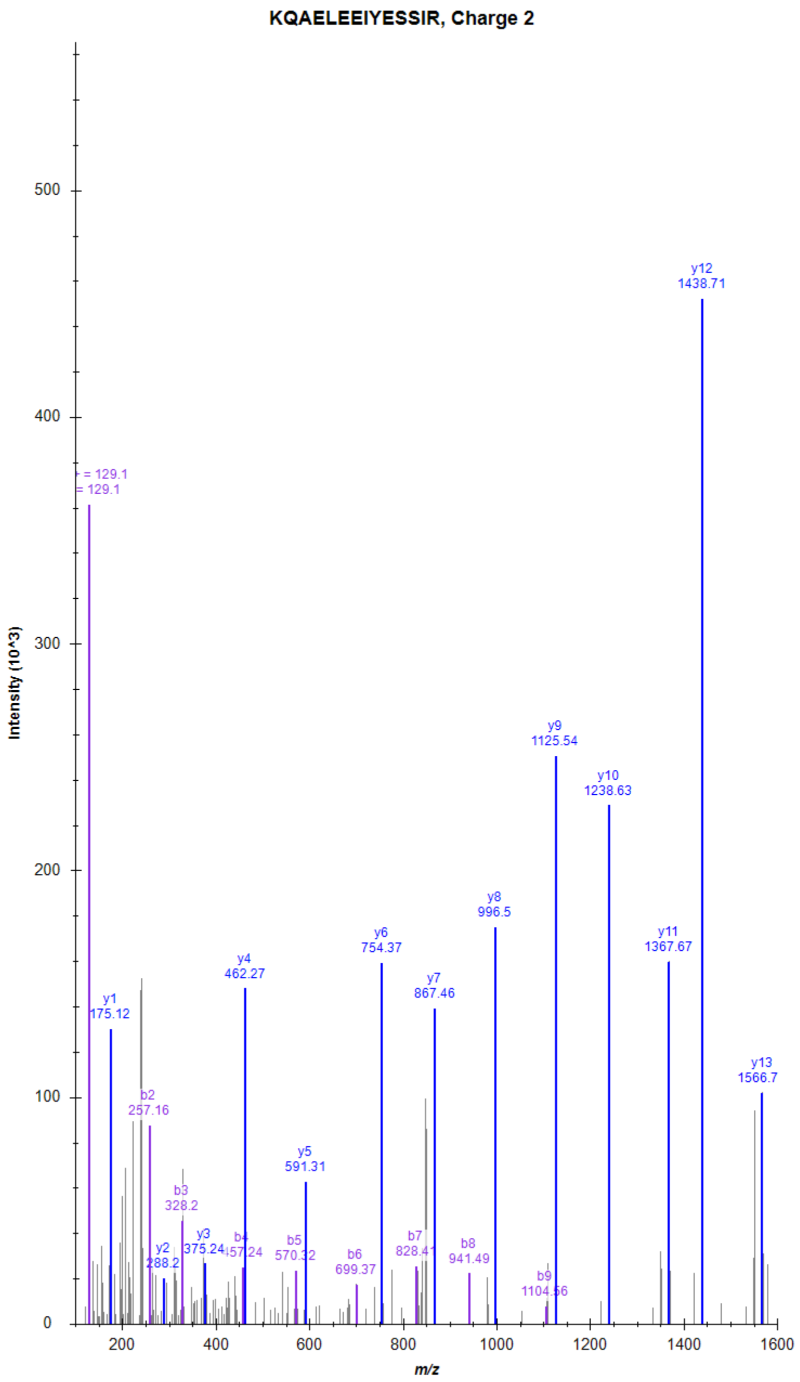

Adhesion G-protein coupled receptor G6 (ADGRG6)

TGLFQDVGPQR, Charge 2, m/z = 609.3173

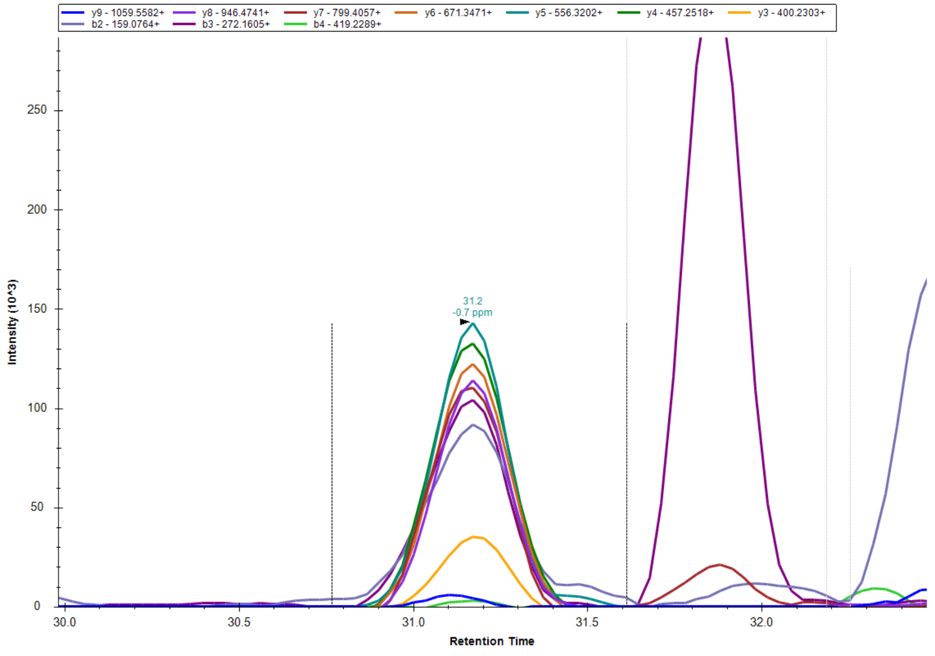

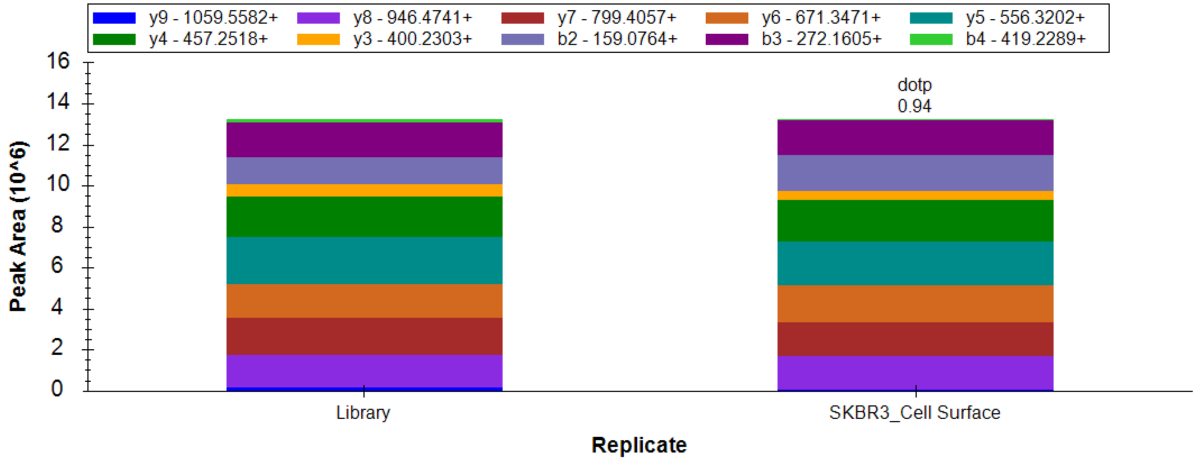

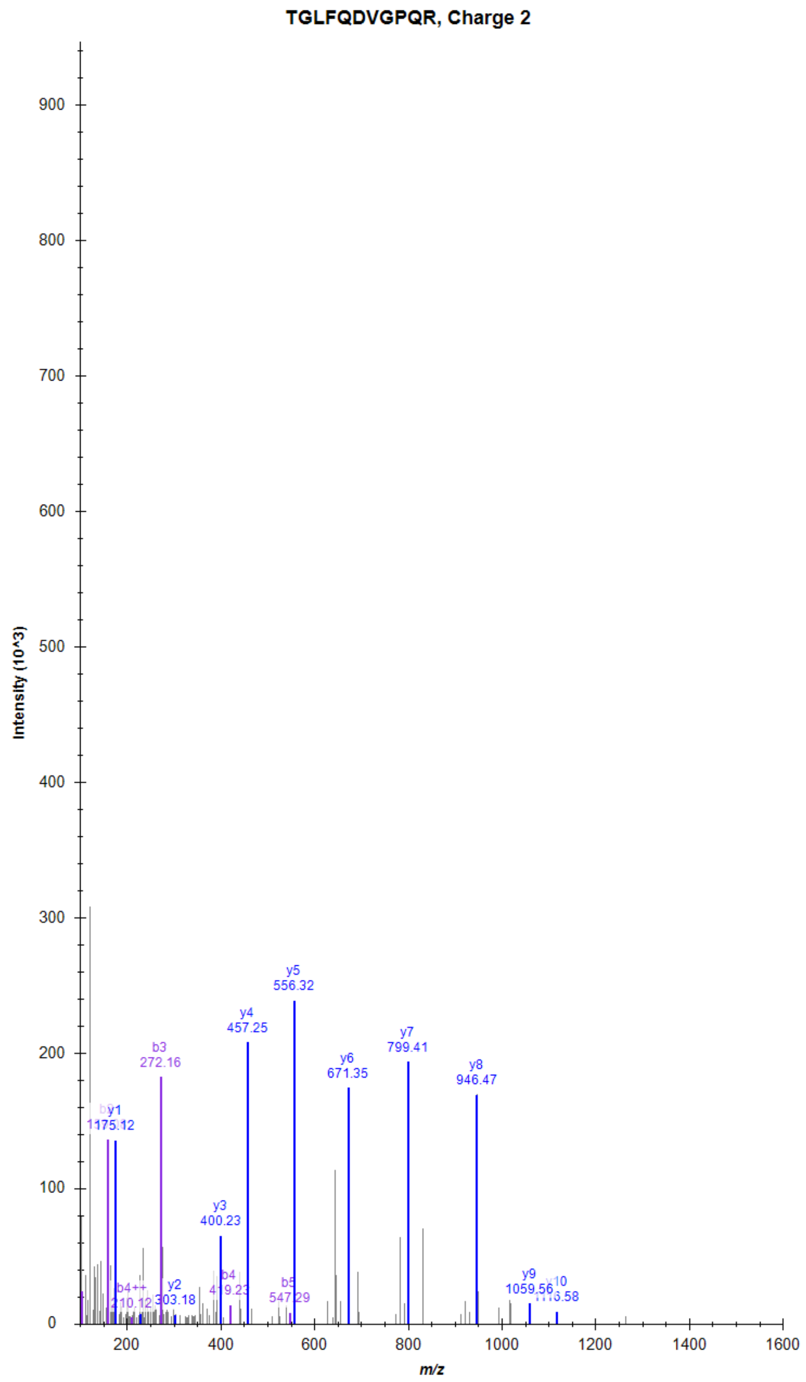

Adhesion G-protein coupled receptor G6 (ADGRG6)

VILPQTSDAYQVSVAK, Charge 2, m/z = 859.9717

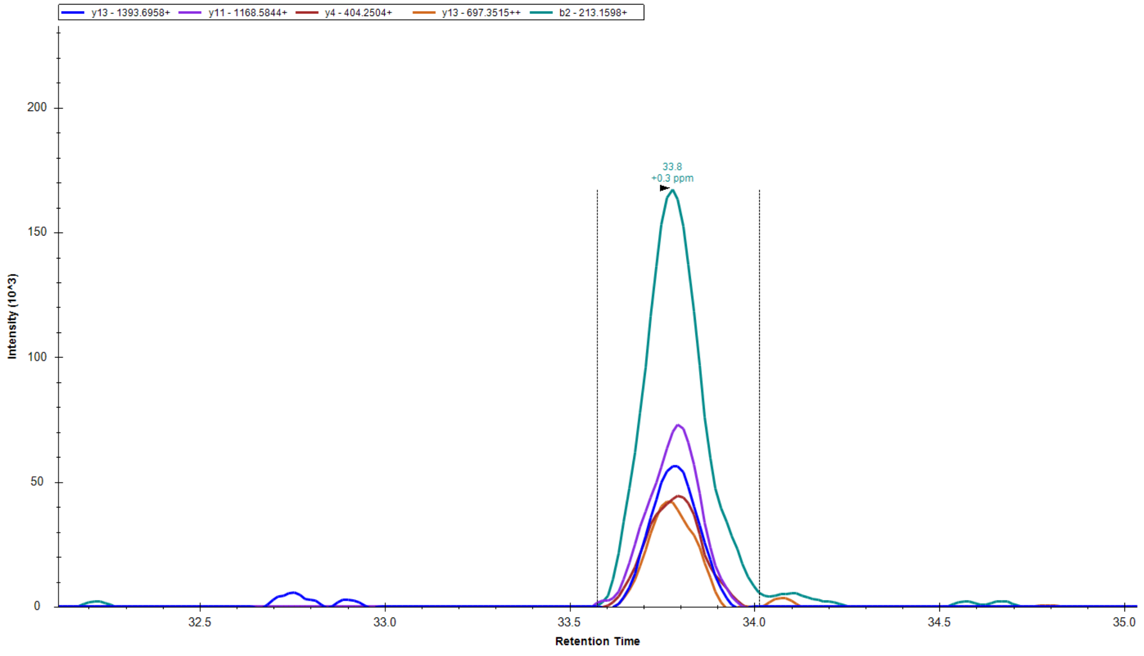

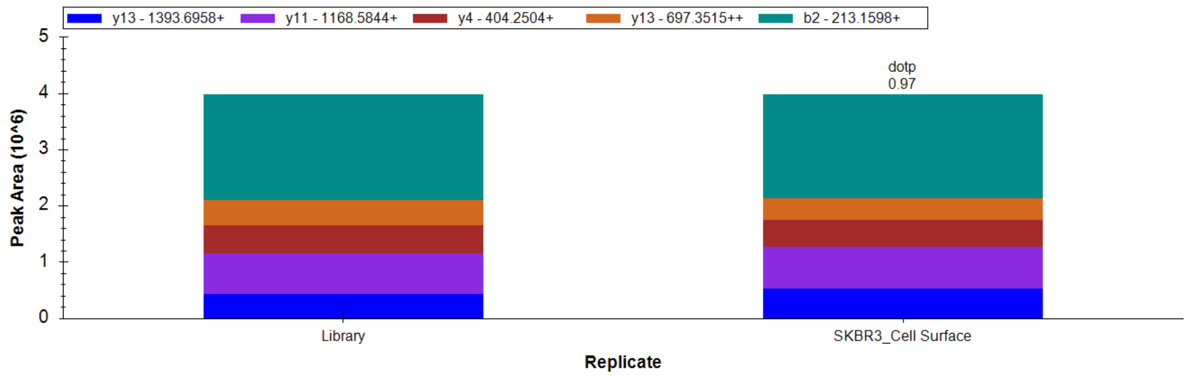

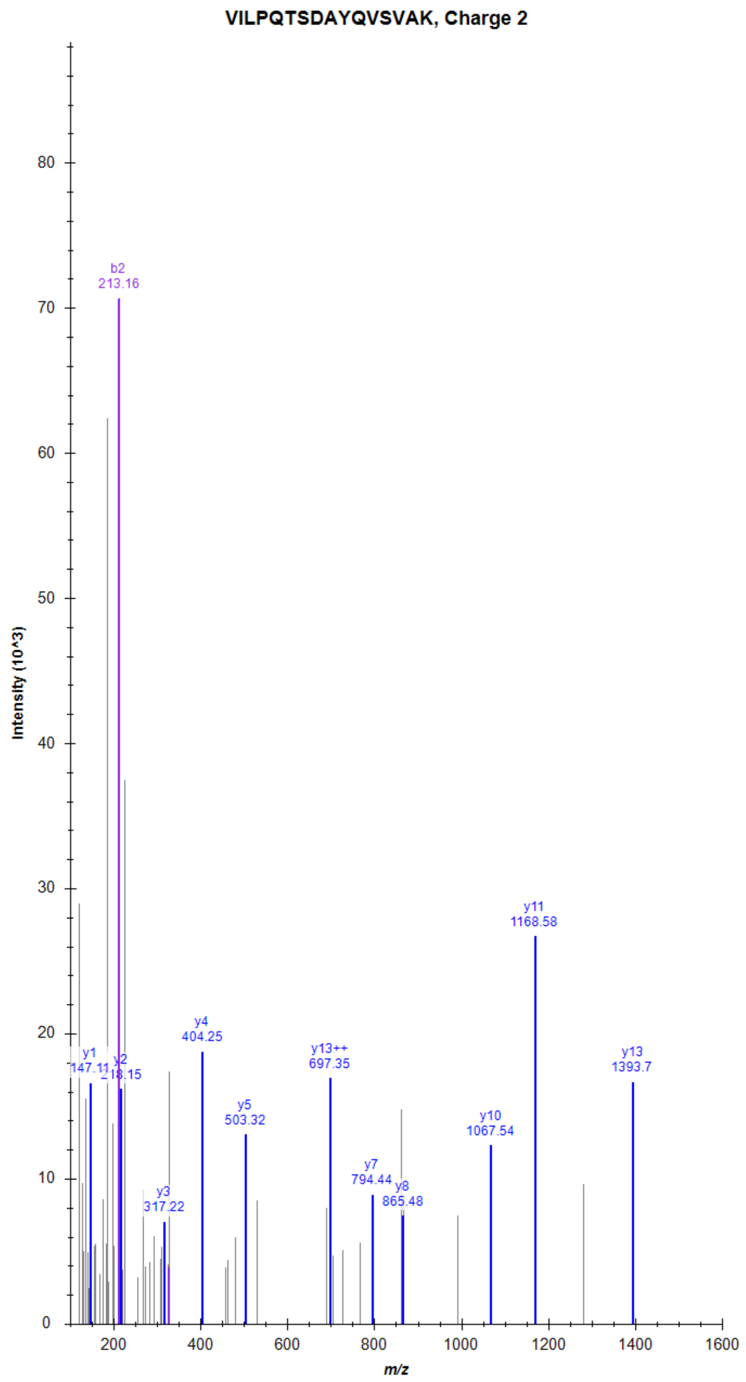

Leucine-rich repeat-containing G-protein coupled receptor 4 (LGR4)

TLDLSYNNIR, Charge 2, m/z = 604.8171

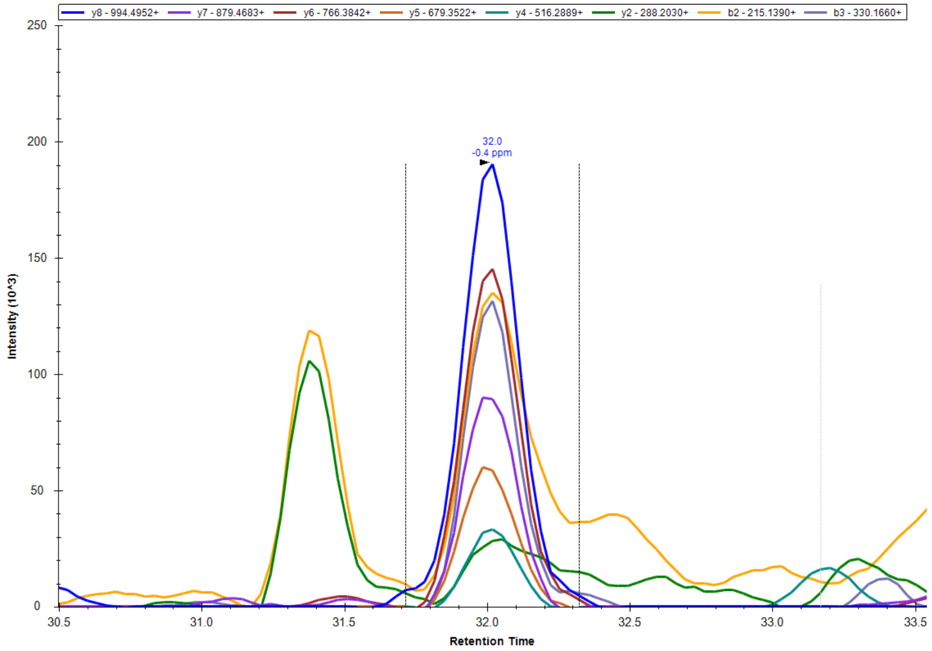

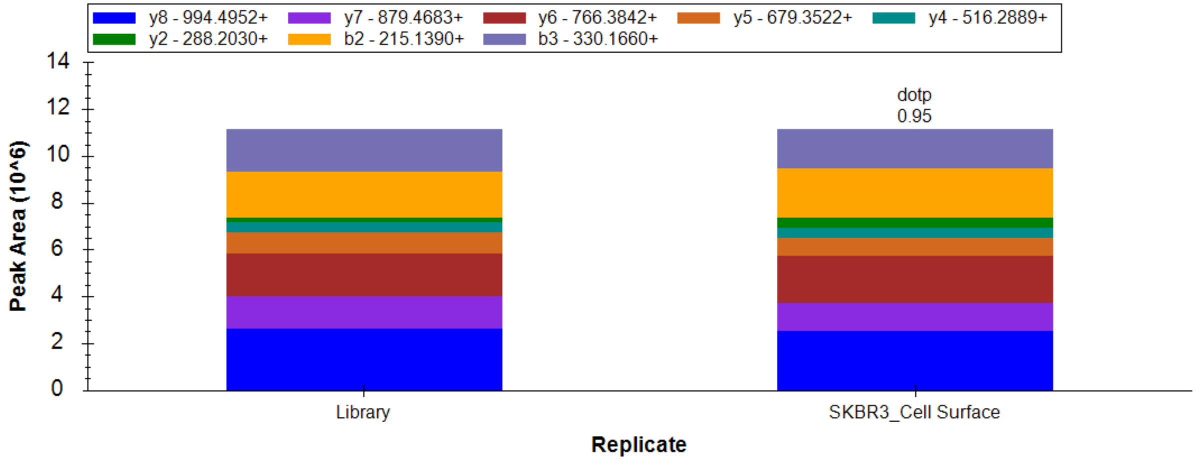

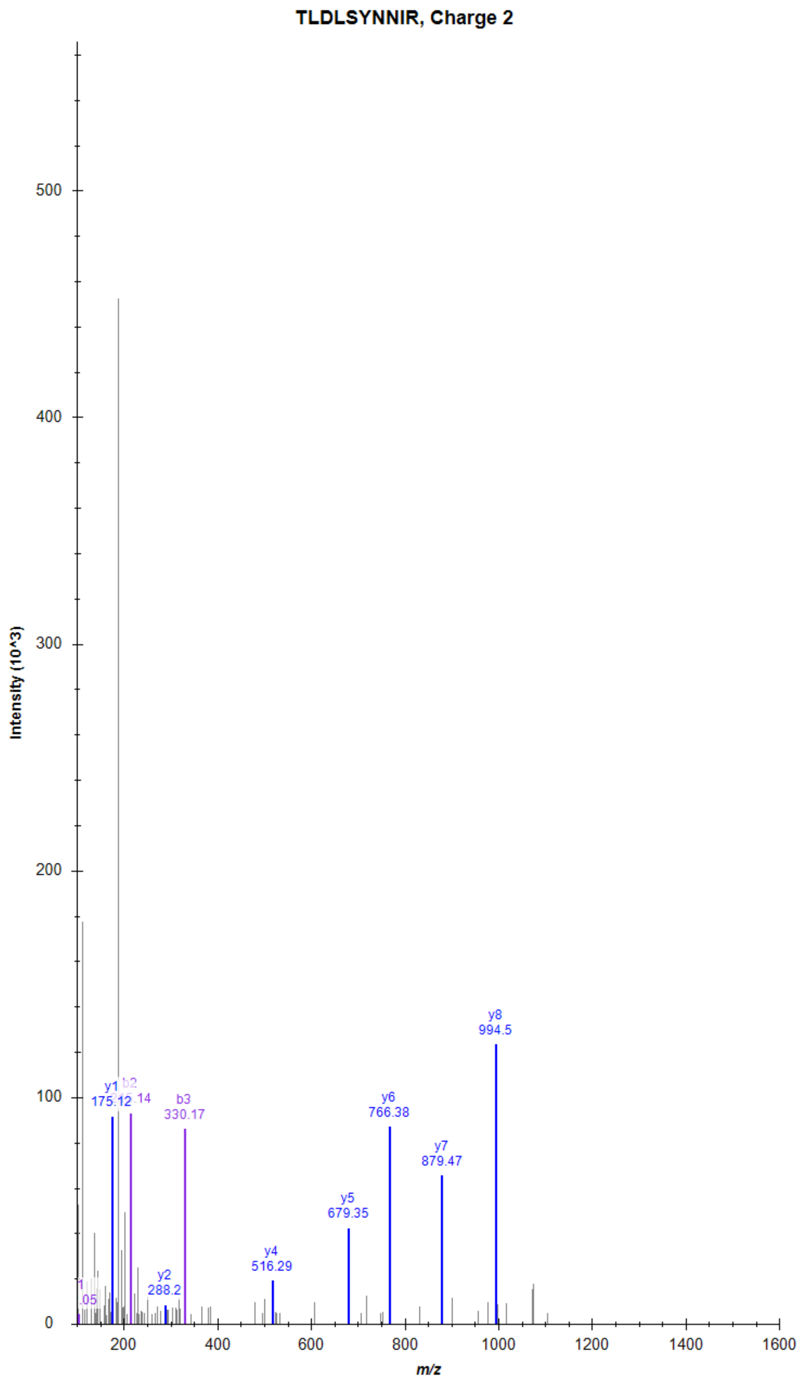

Leucine-rich repeat-containing G-protein coupled receptor 4 (LGR4)

VLTLQNNQLK, Charge 2, m/z = 585.8464

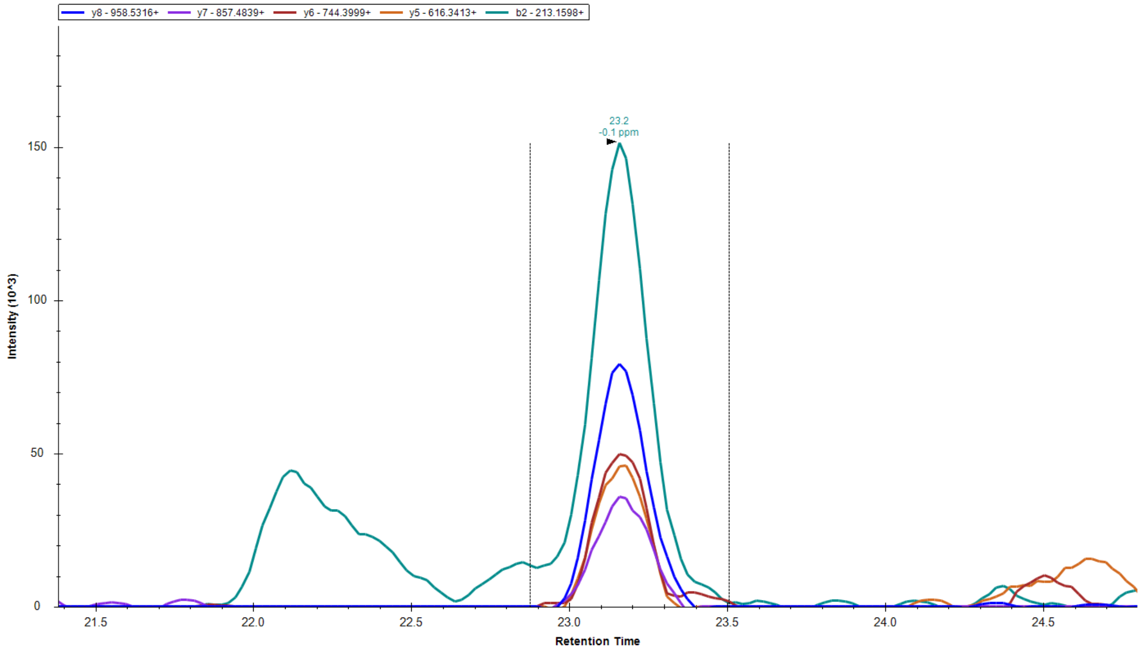

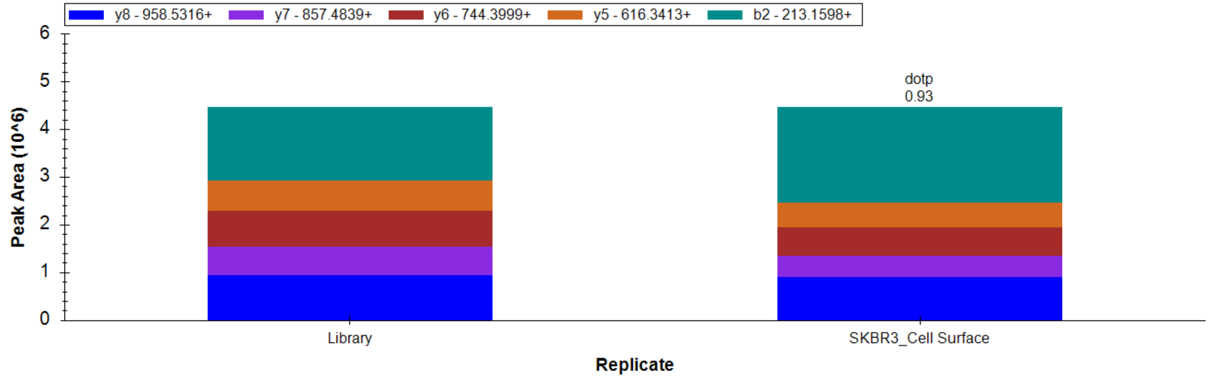

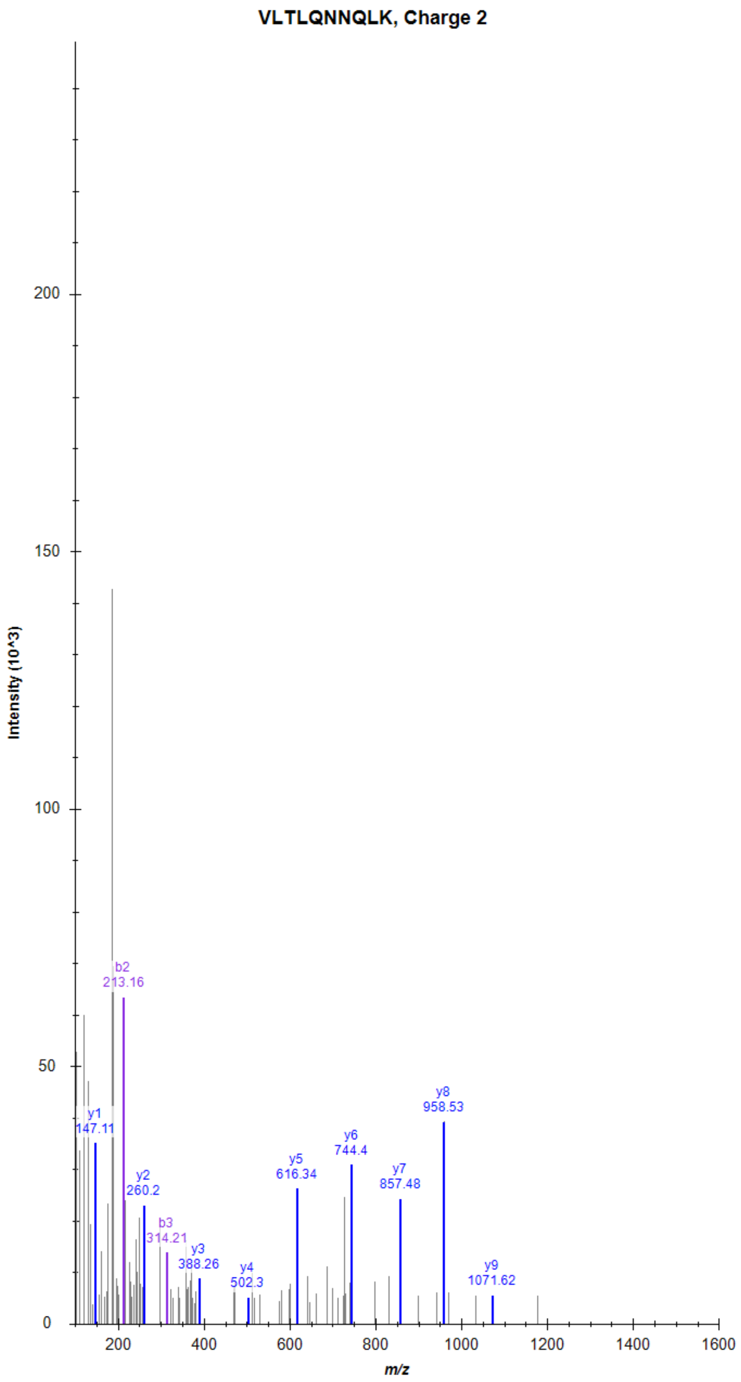

Cadherin EGF LAG seven-pass G-type receptor 1 (CELSR1)

LVDTASTFLGGGSAGPK, Charge 2, m/z = 789.4125

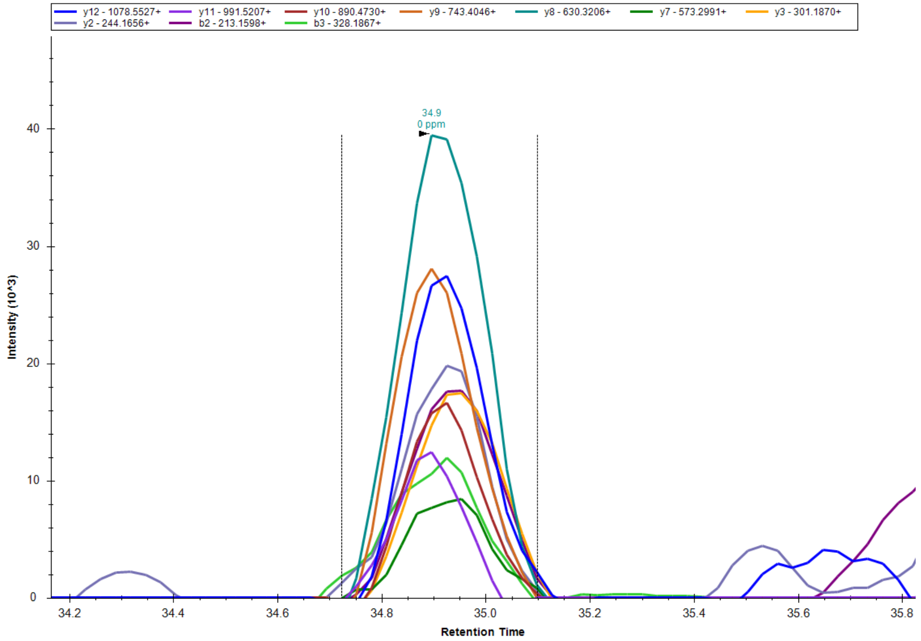

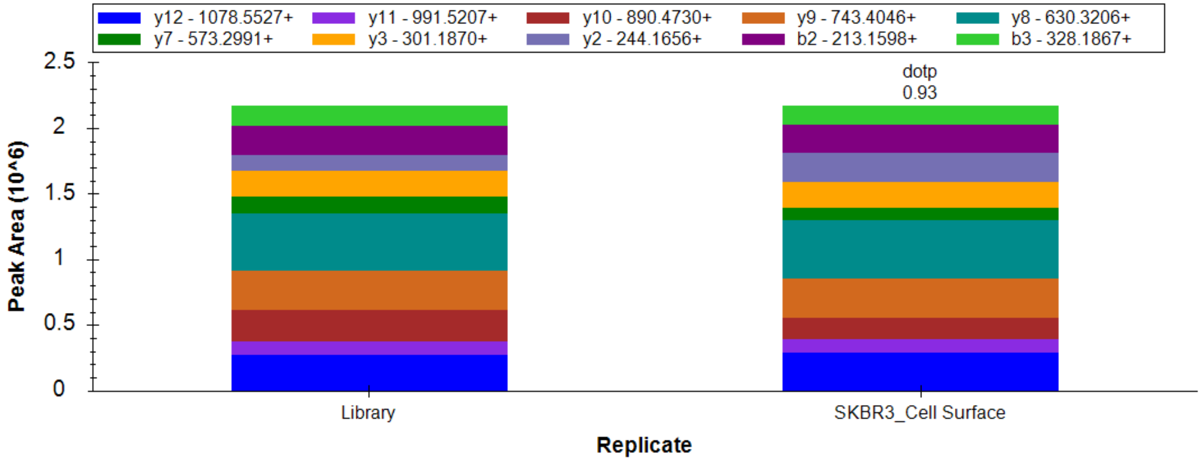

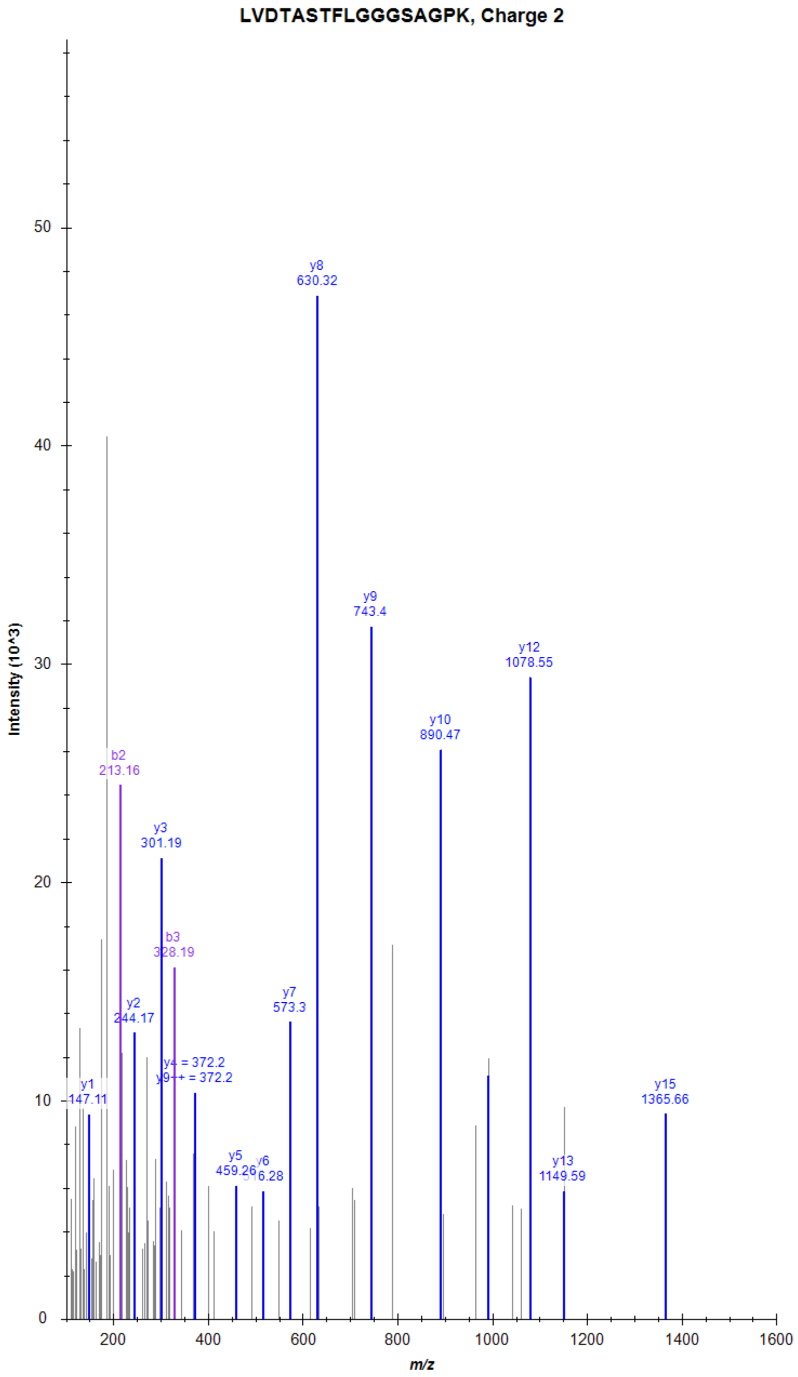

Cadherin EGF LAG seven-pass G-type receptor 1 (CELSR1)

LLLLDPATGELQLSR, Charge 2, m/z = 819.9744

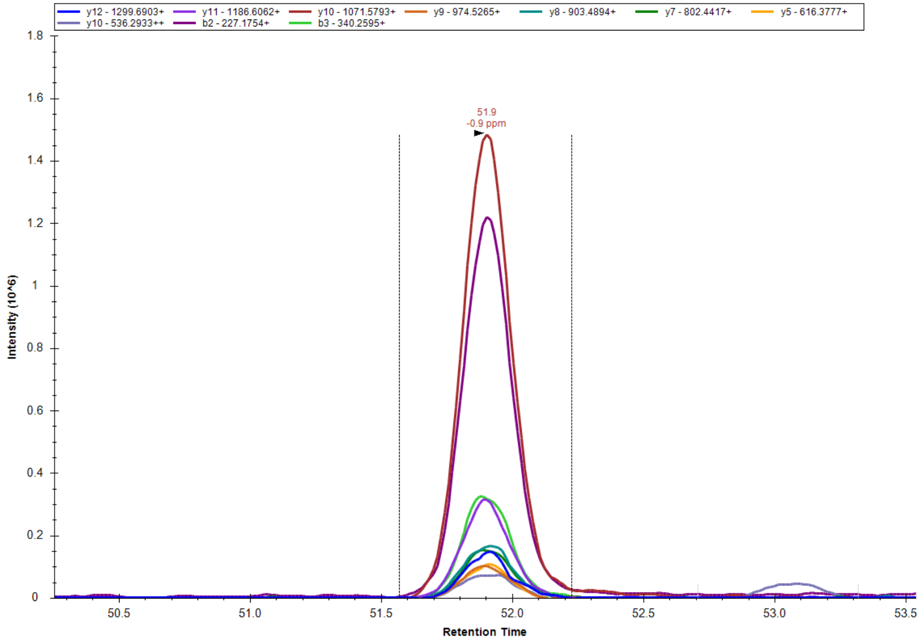

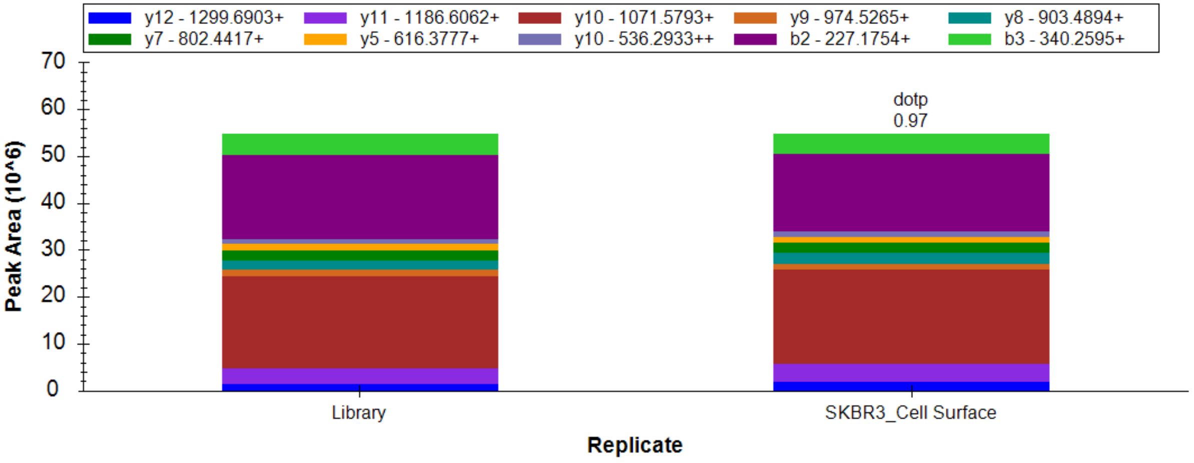

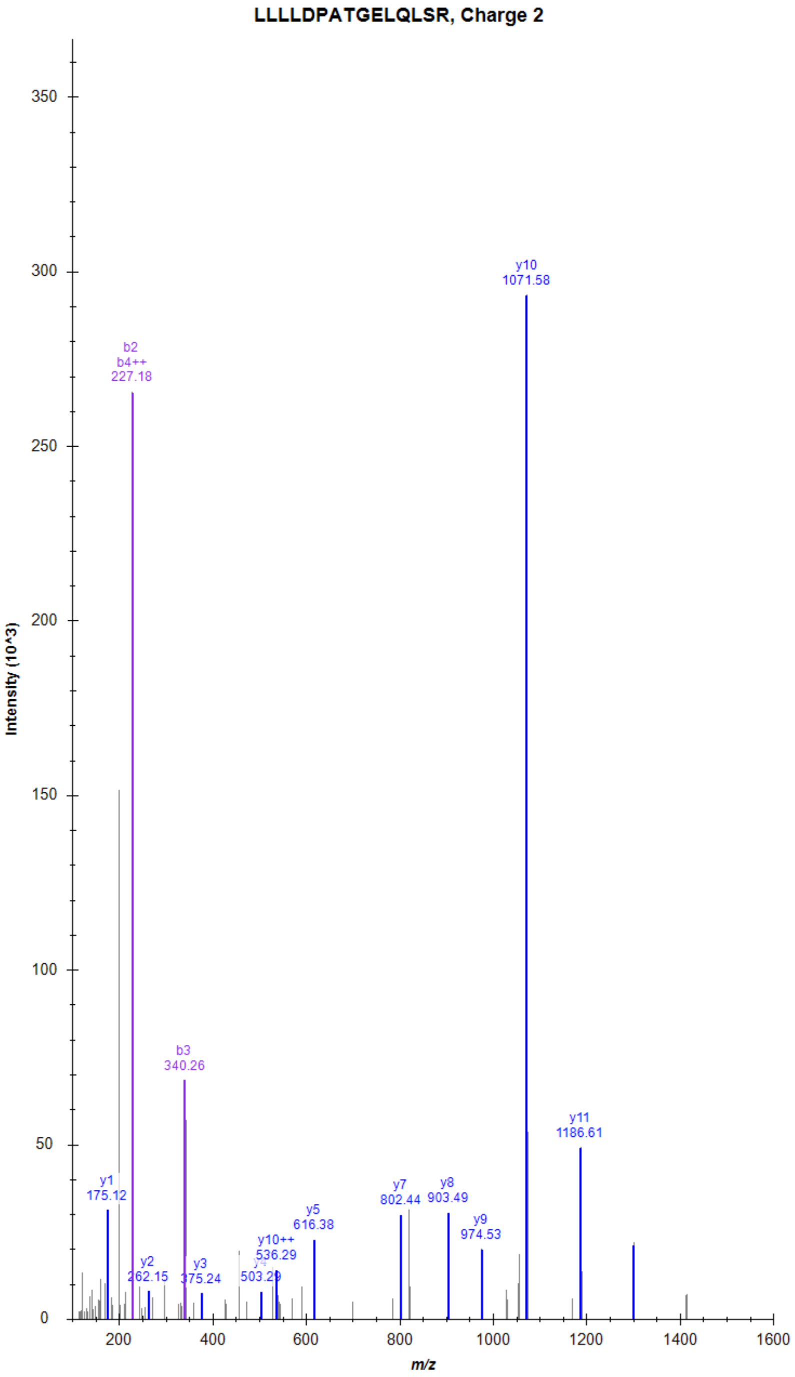

Adhesion G-protein coupled receptor G1 (ADGRG1)

LQPTAGLQDLHIHSR, Charge 3, m/z = 562.6406

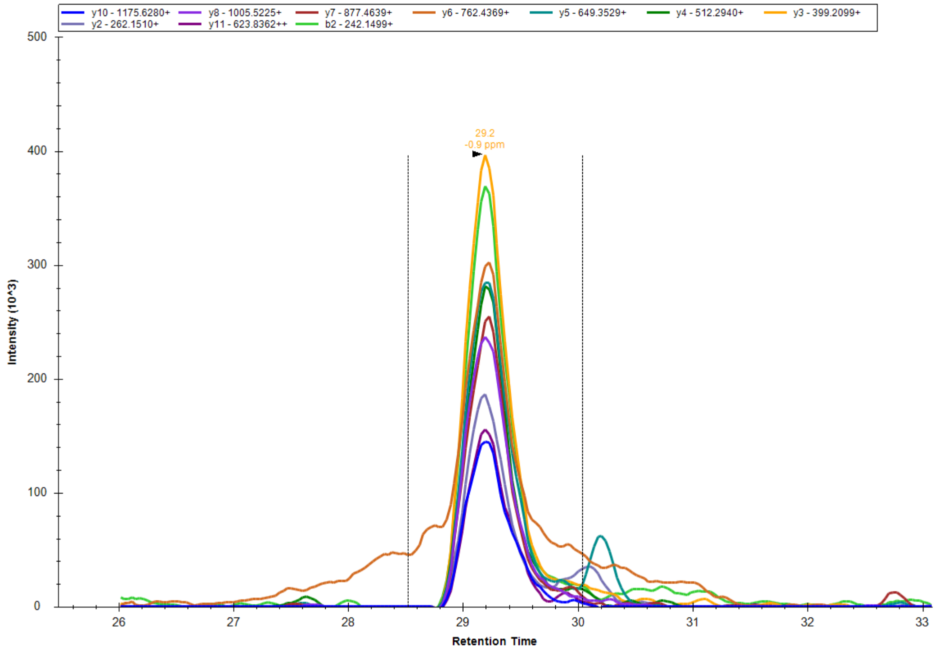

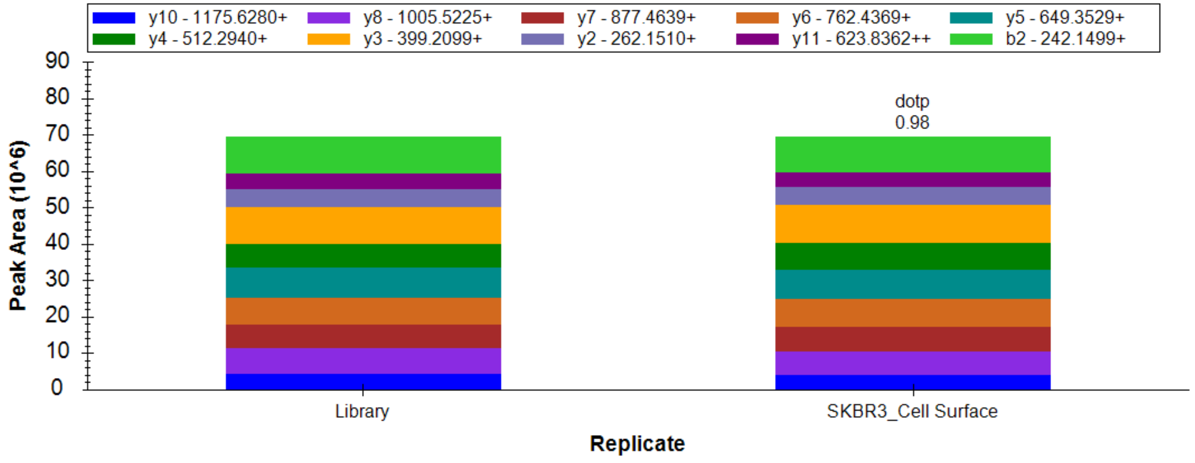

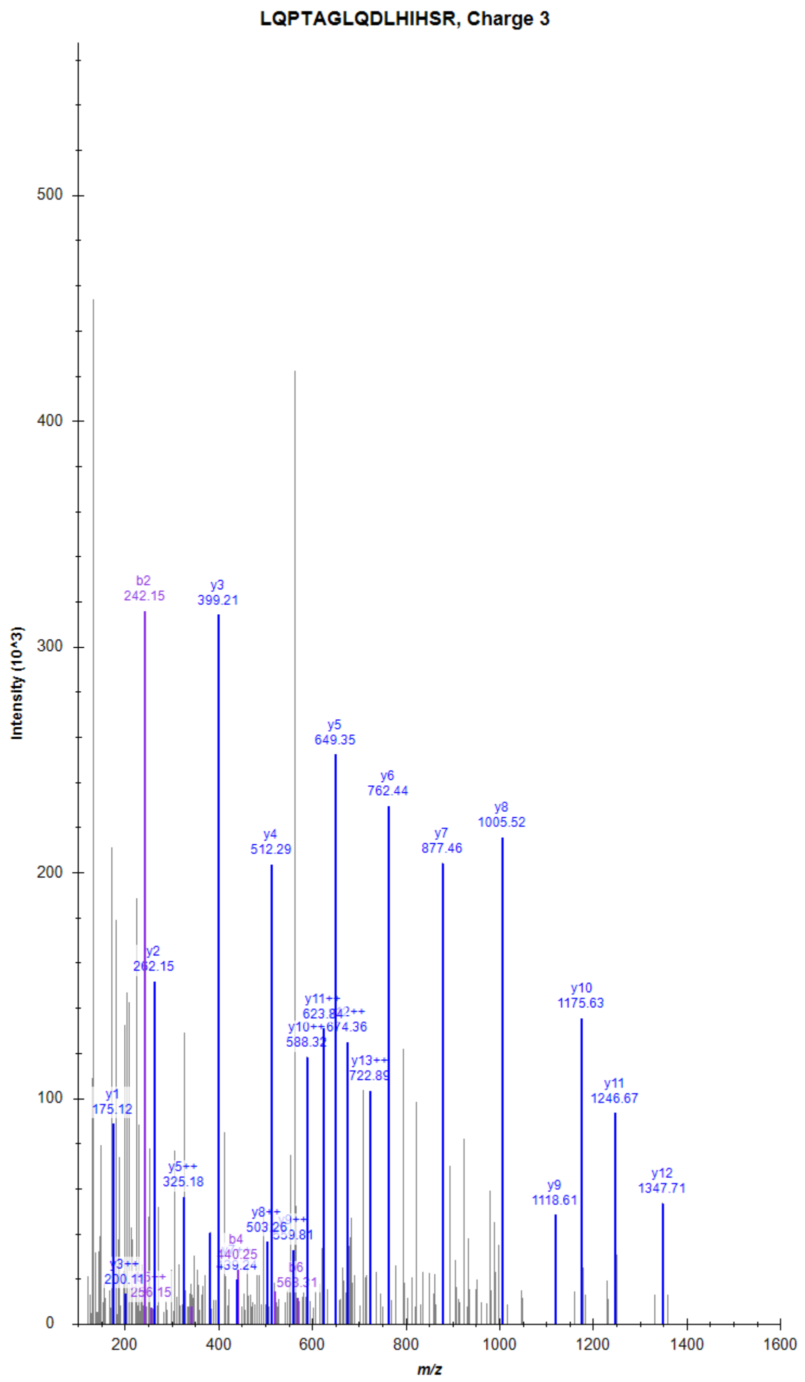

Adhesion G-protein coupled receptor G1 (ADGRG1)

QEEEQSEIMEYSVLLPR, Charge 2, m/z = 1040.4957

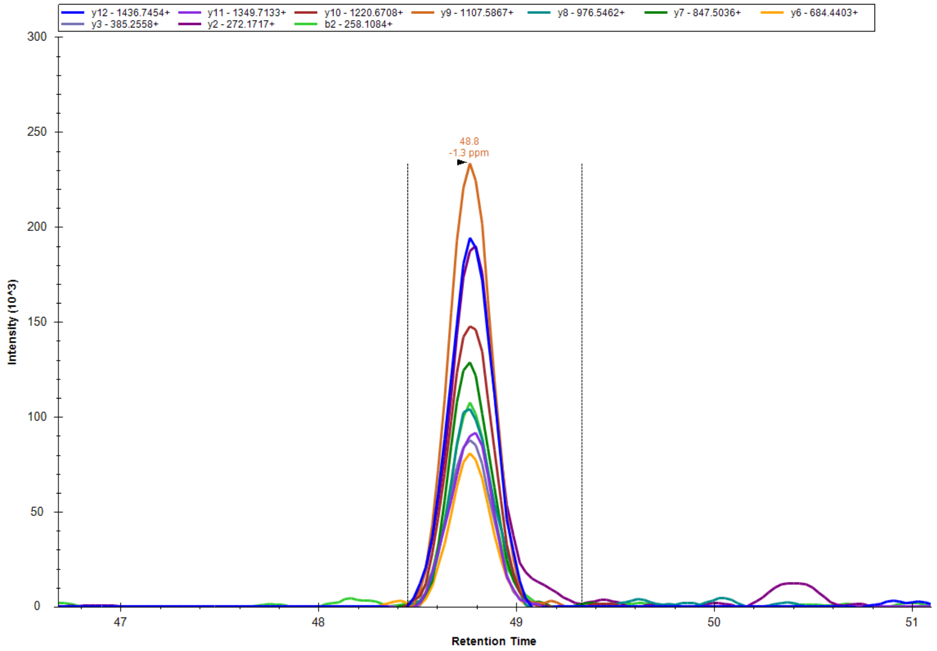

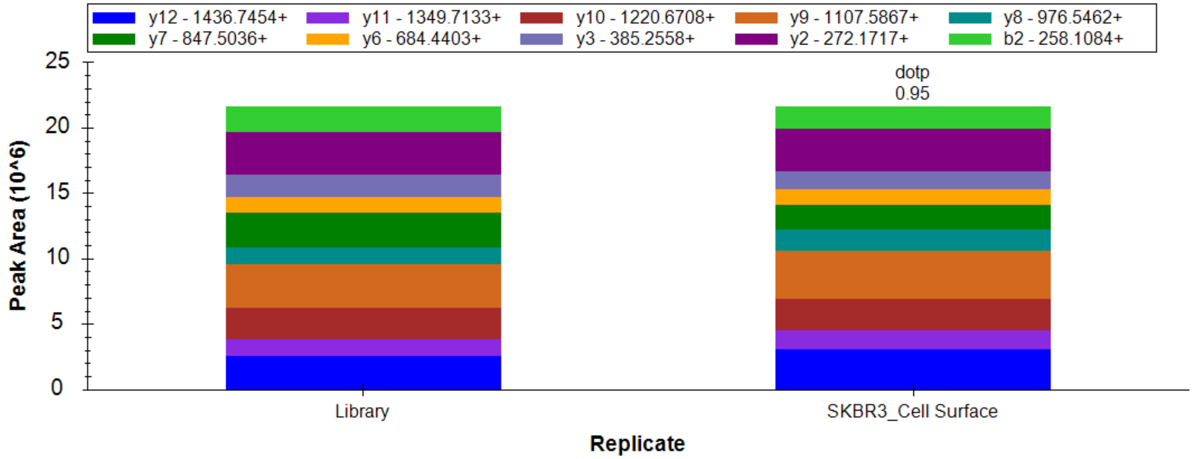

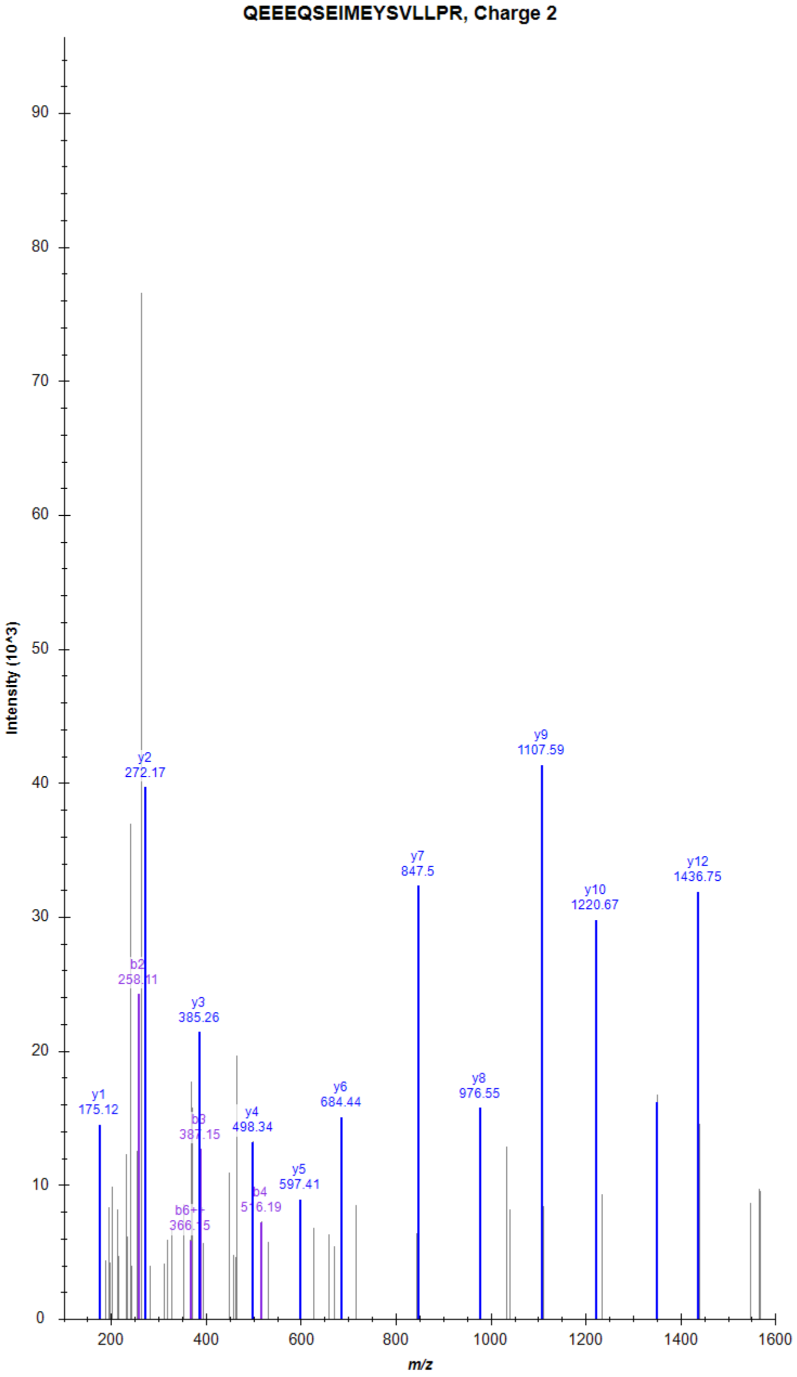

Adhesion G-protein coupled receptor E2 (ADGRE2)

SGDPGPSVVGLVSIPGMGK, Charge 2, m/z = 877.4613

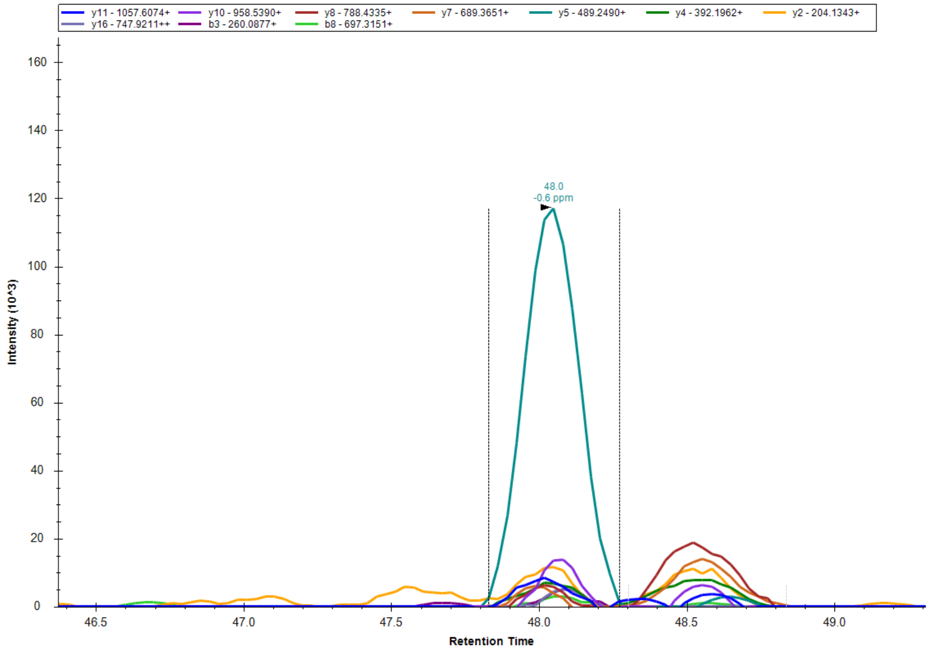

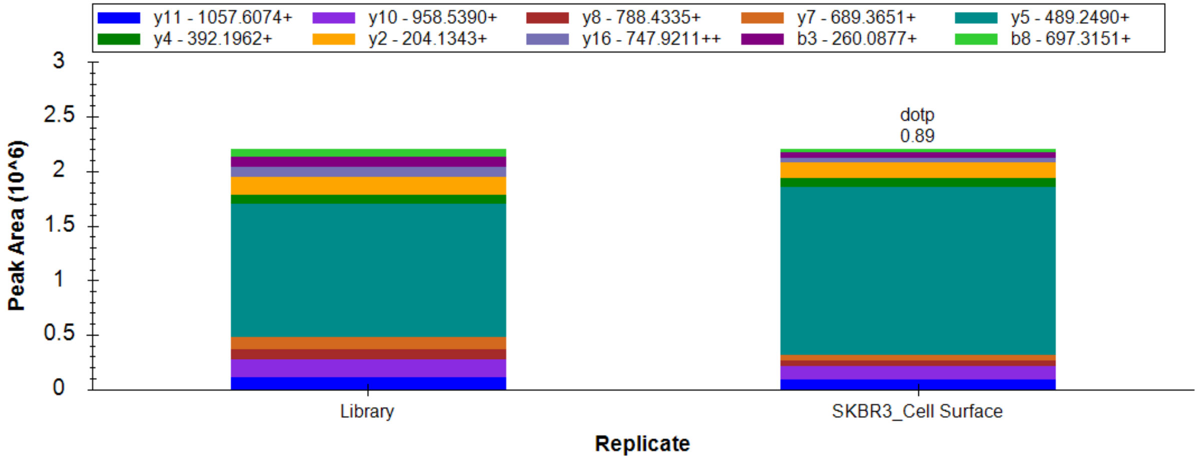

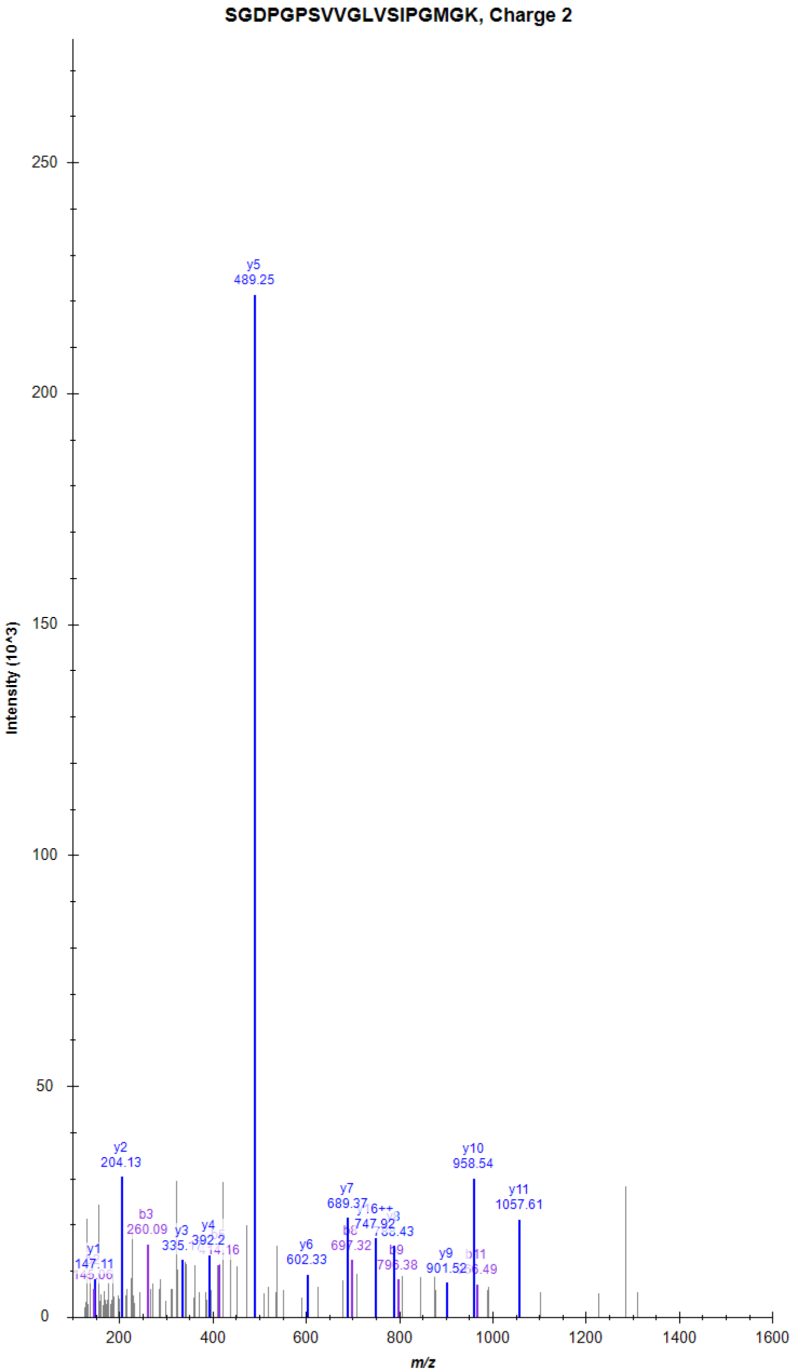

Adhesion G-protein coupled receptor E2 (ADGRE2)

LLAEAPLVLEPEK, Charge 2, m/z = 711.4155

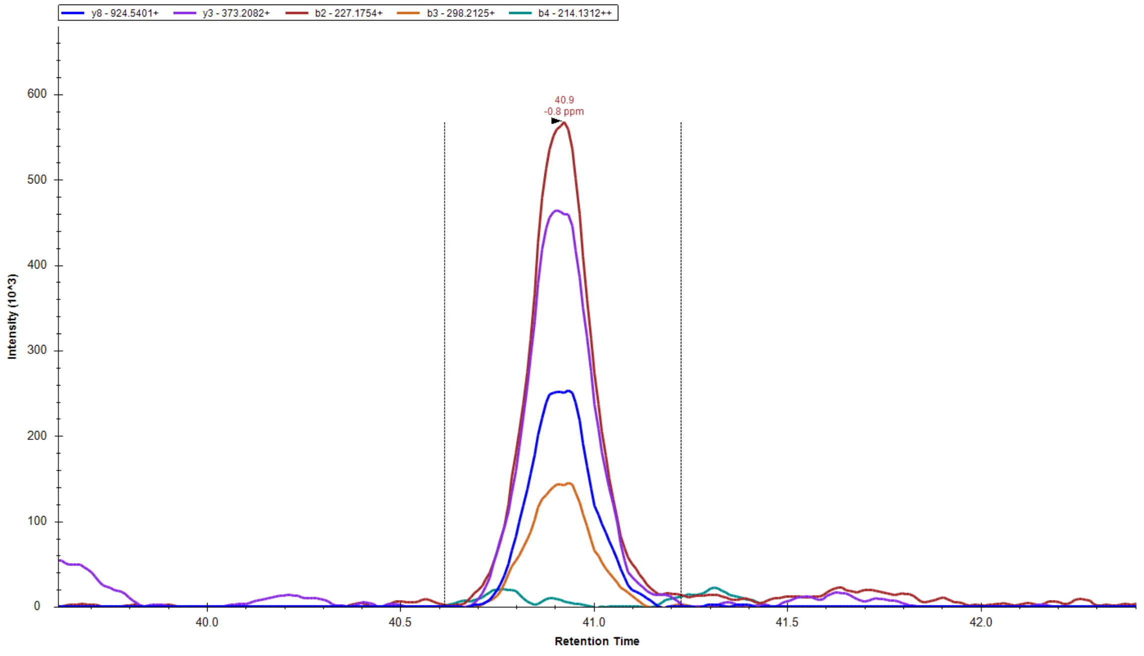

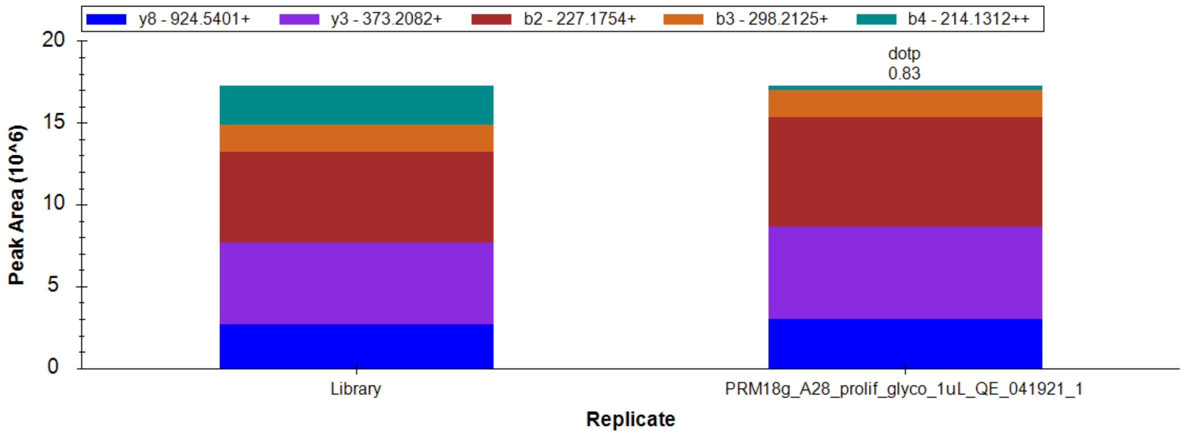

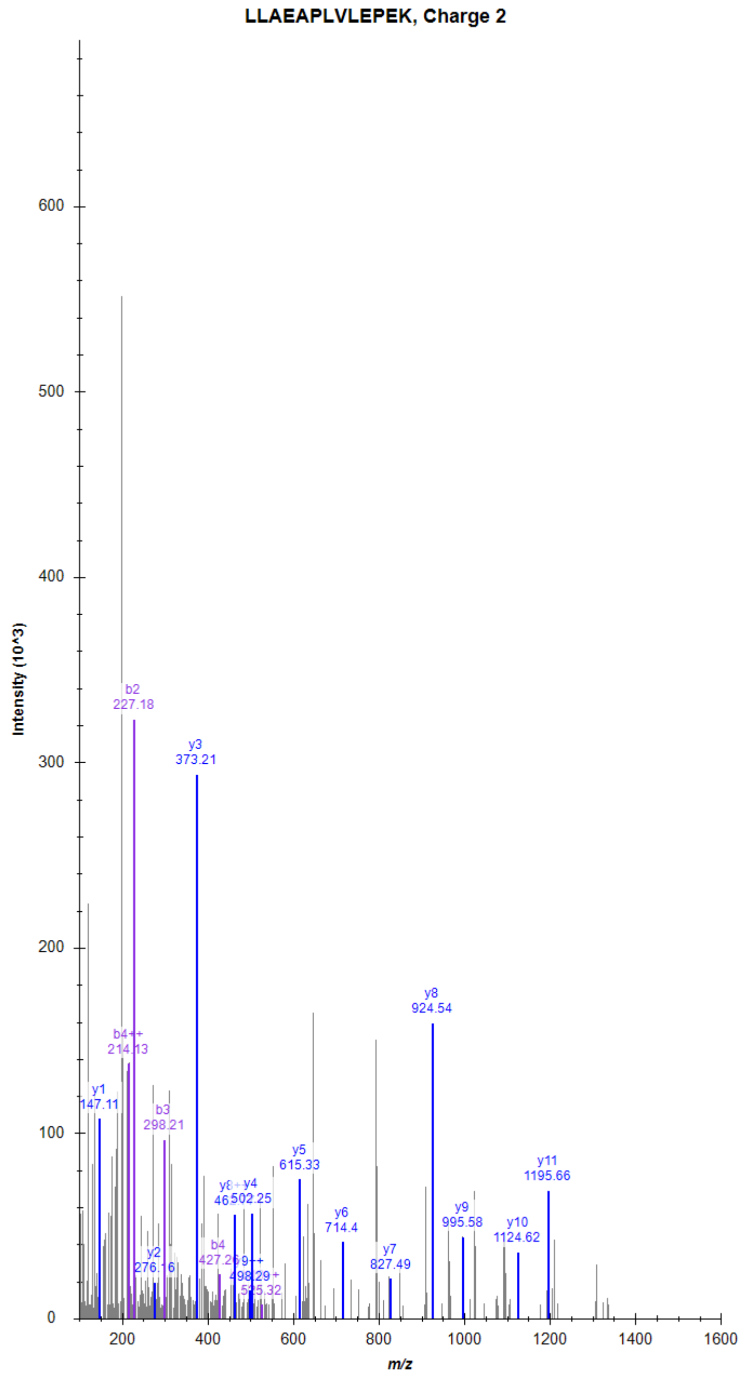

Muscarinic acetylcholine receptor M1 (CHRM1)

MPMVDPEAQAPTK, Charge 2, m/z = 707.8388

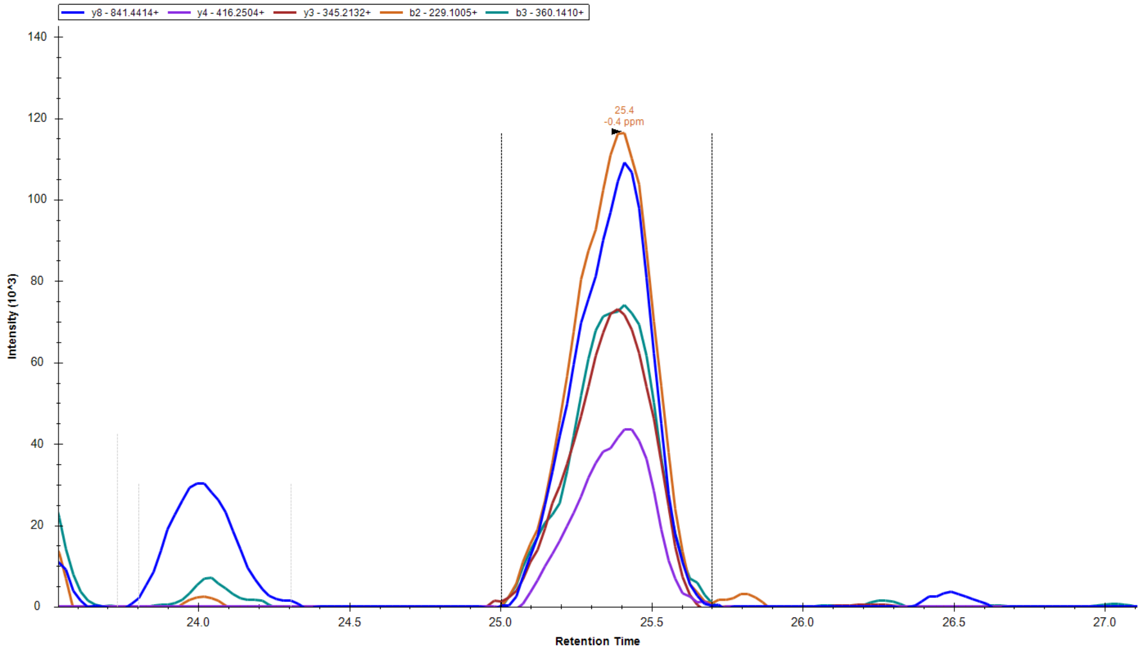

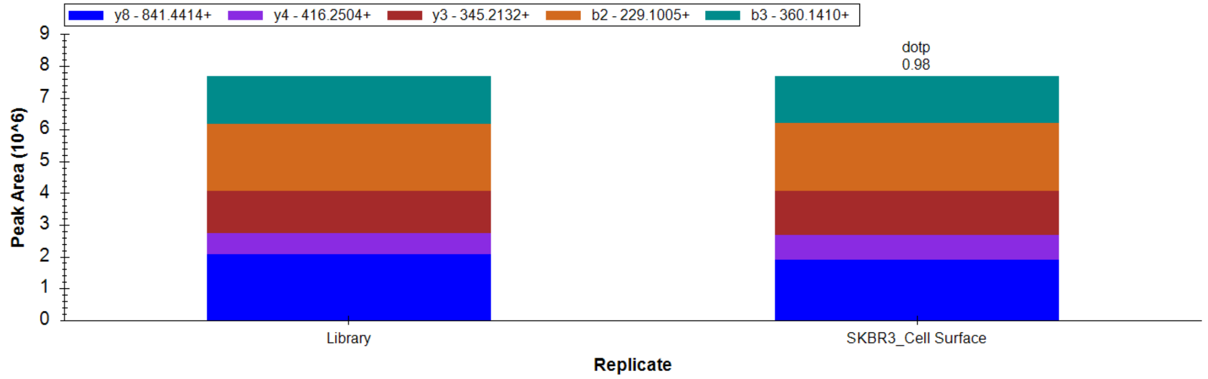

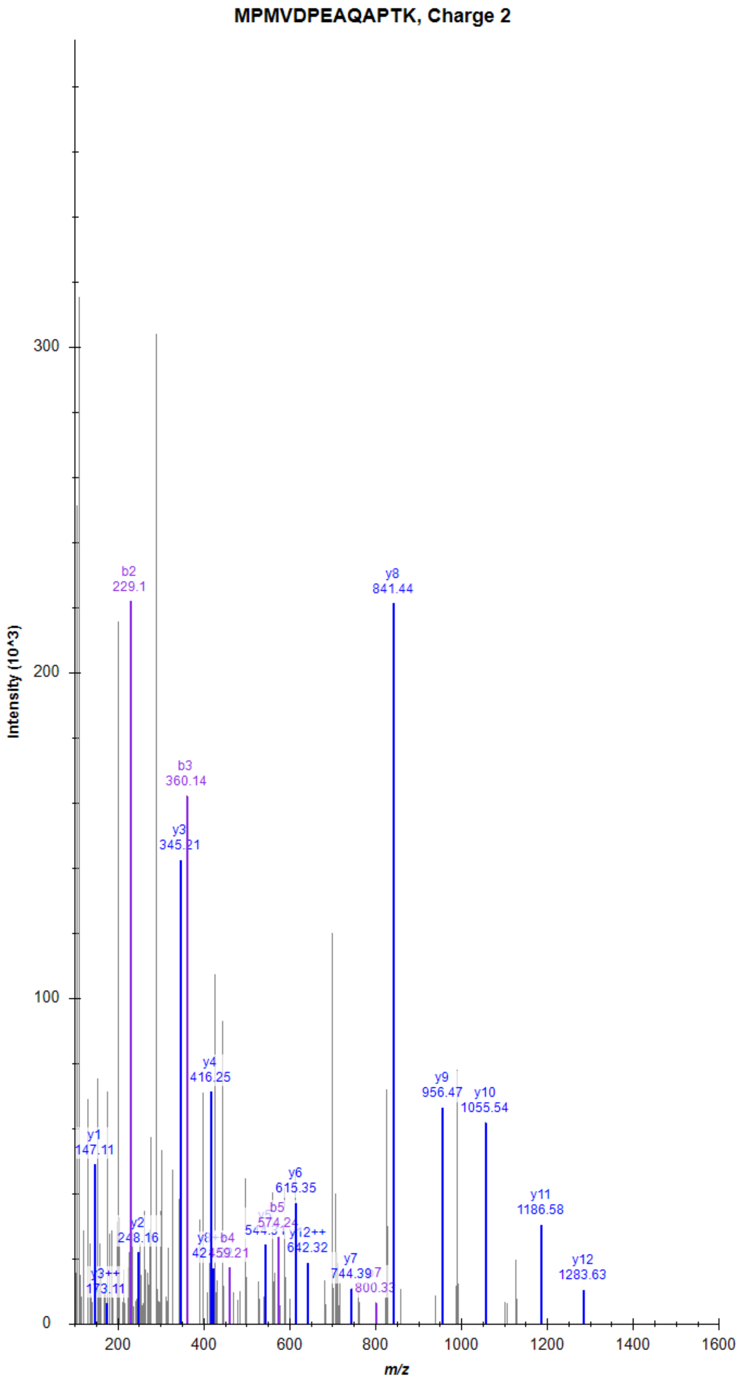

Muscarinic acetylcholine receptor M1 (CHRM1)

ELAALQGSETPGK, Charge 2, m/z = 650.8407

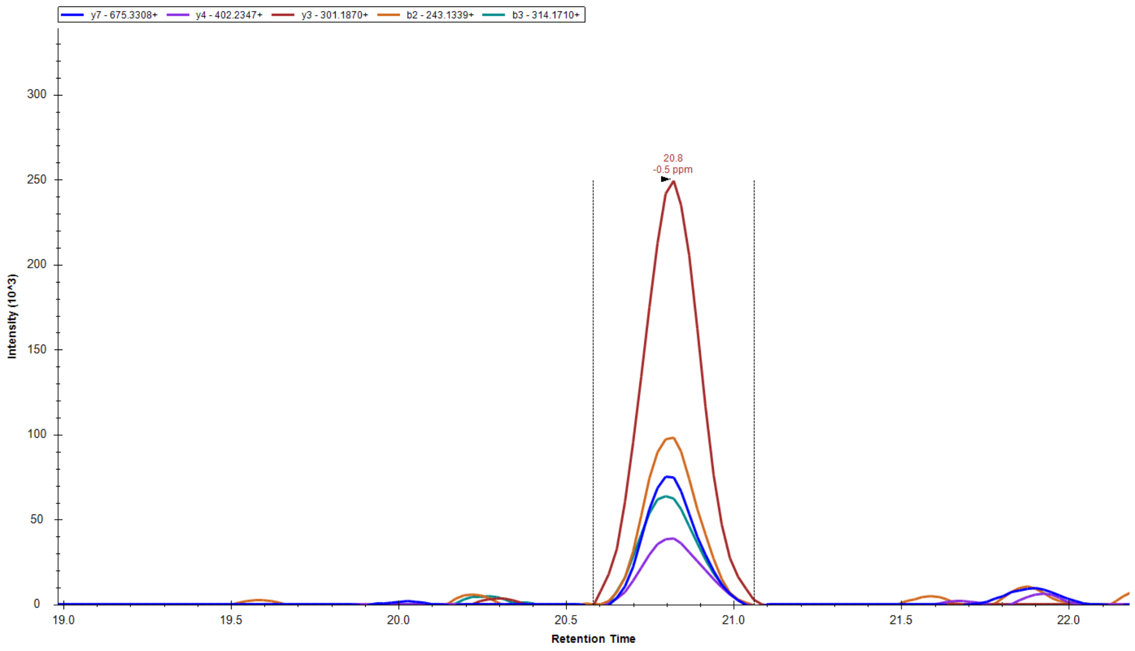

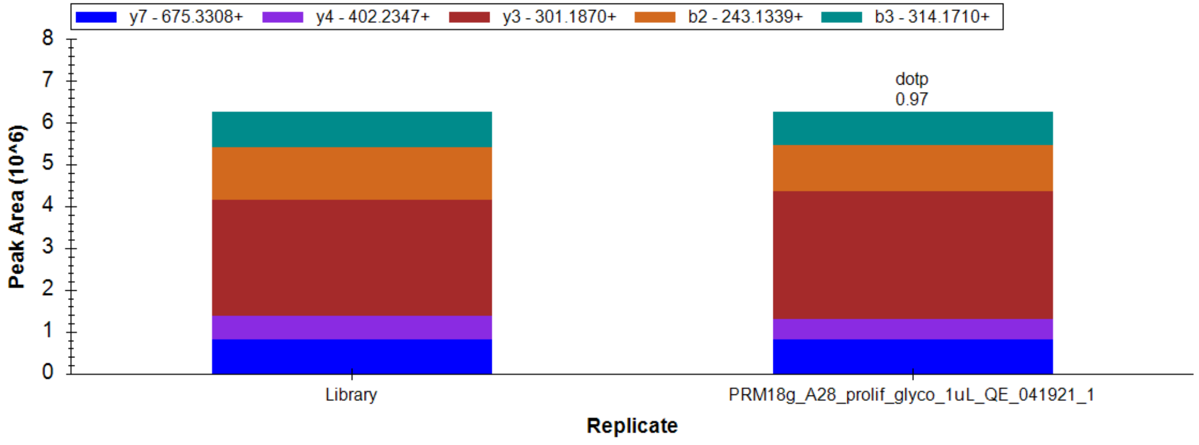

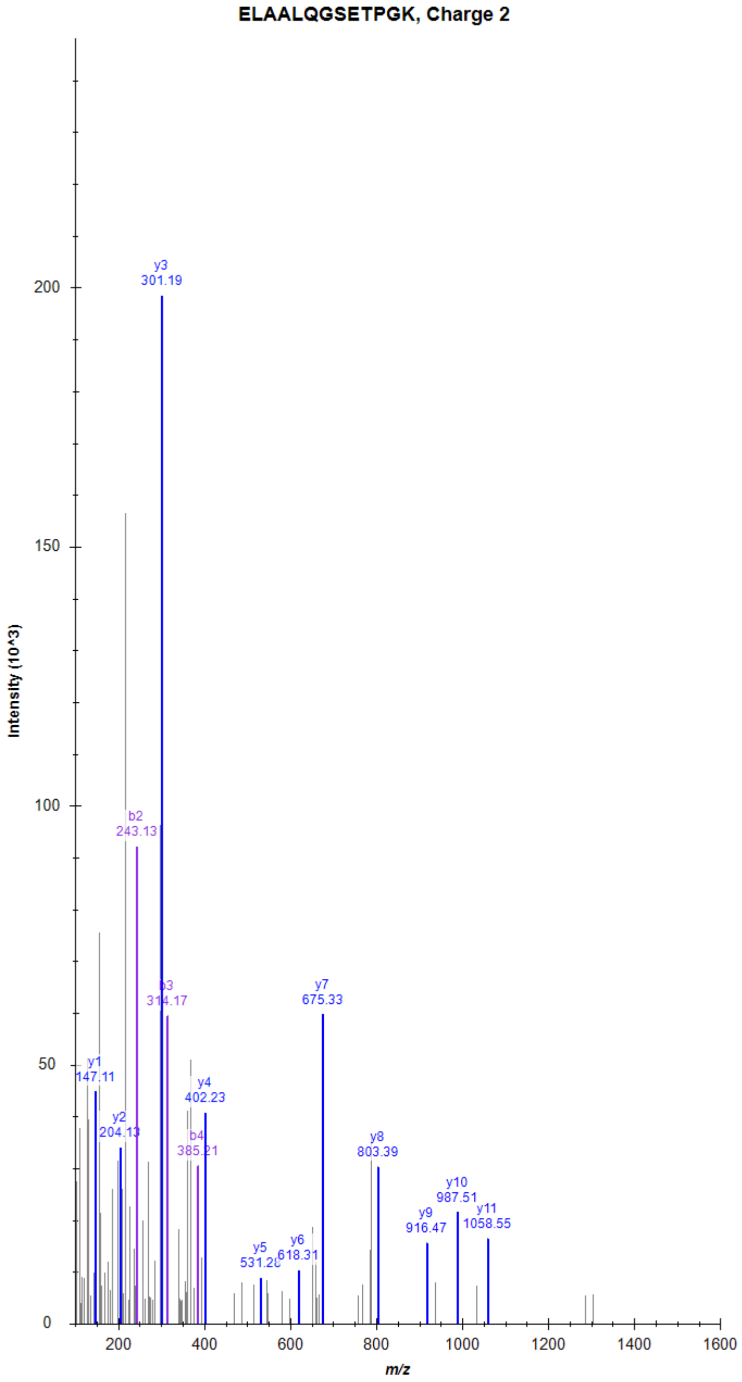

P2Y purinoceptor 2 (P2RY2)

LLKPAYGTSGGLPR, Charge 3, m/z = 477.2769

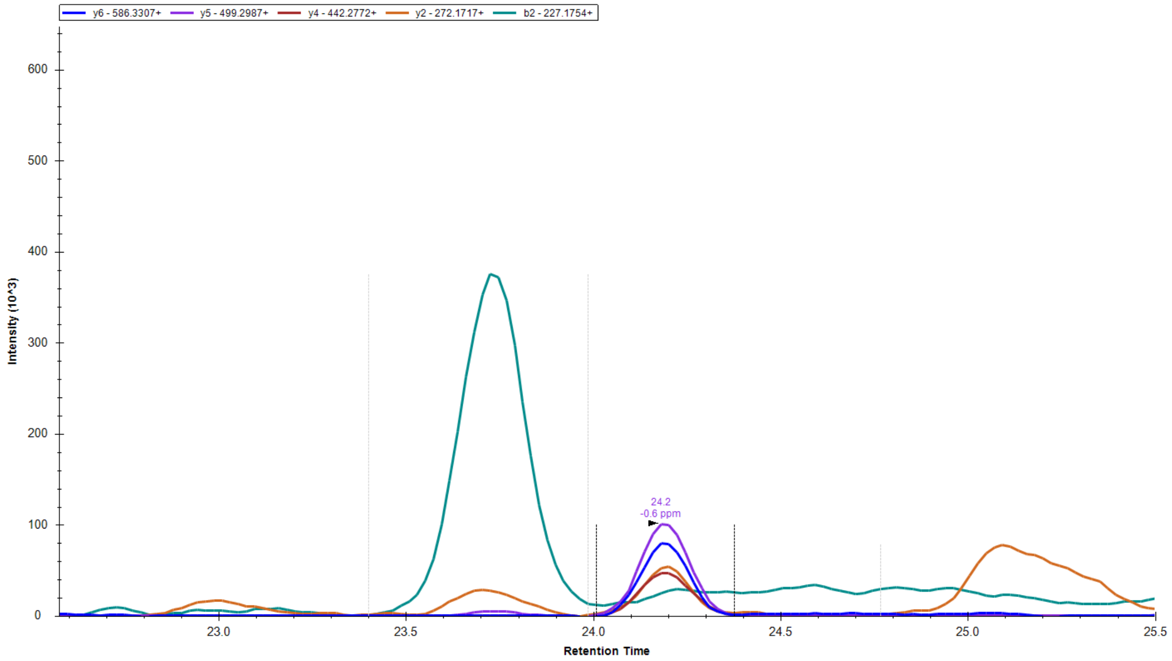

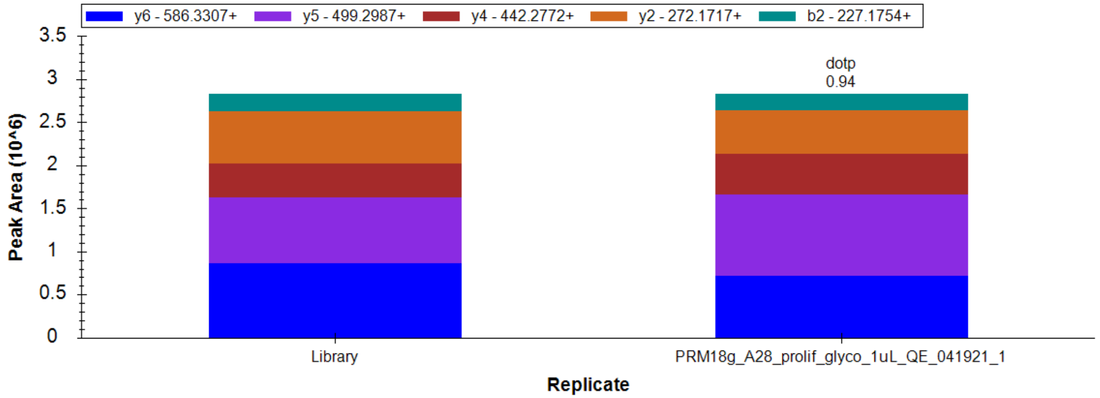

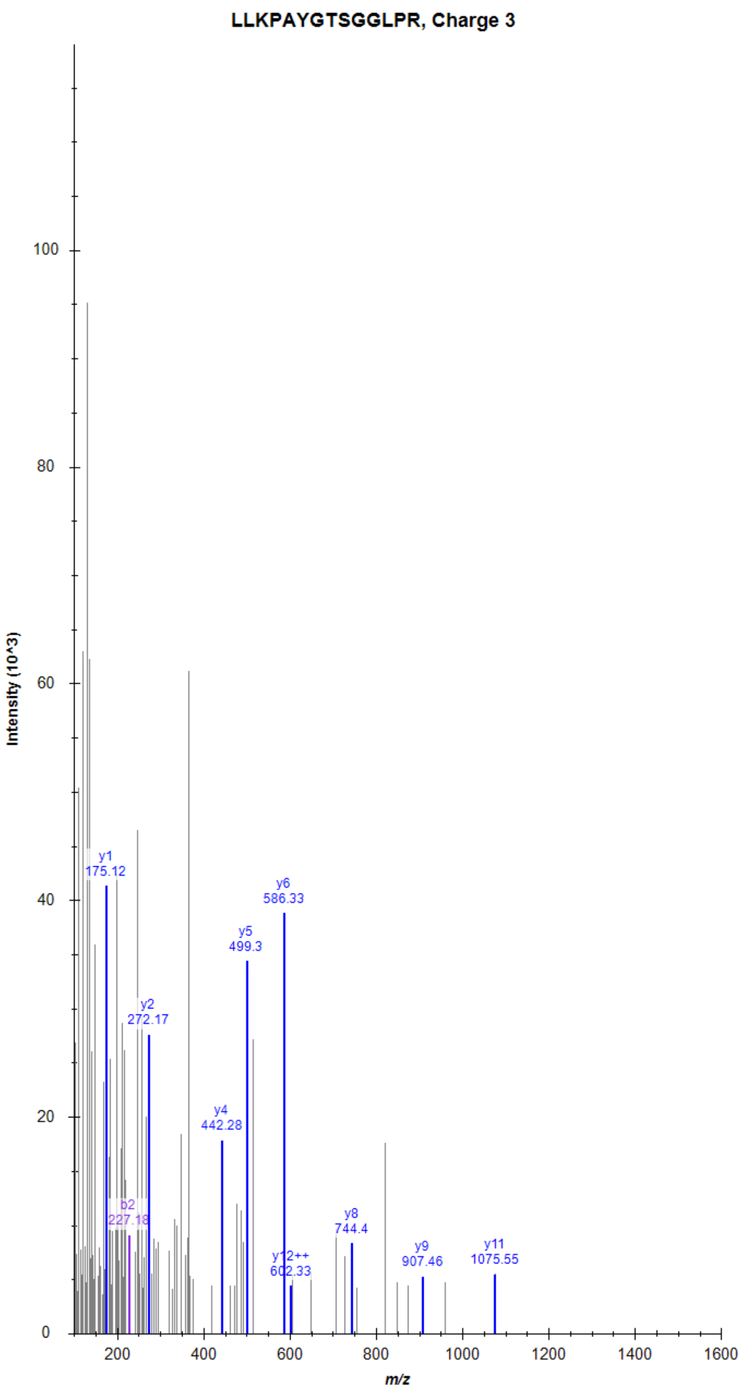

P2Y purinoceptor 2 (P2RY2)

IEDVLGSSEDSR, Charge 2, m/z = 653.8104

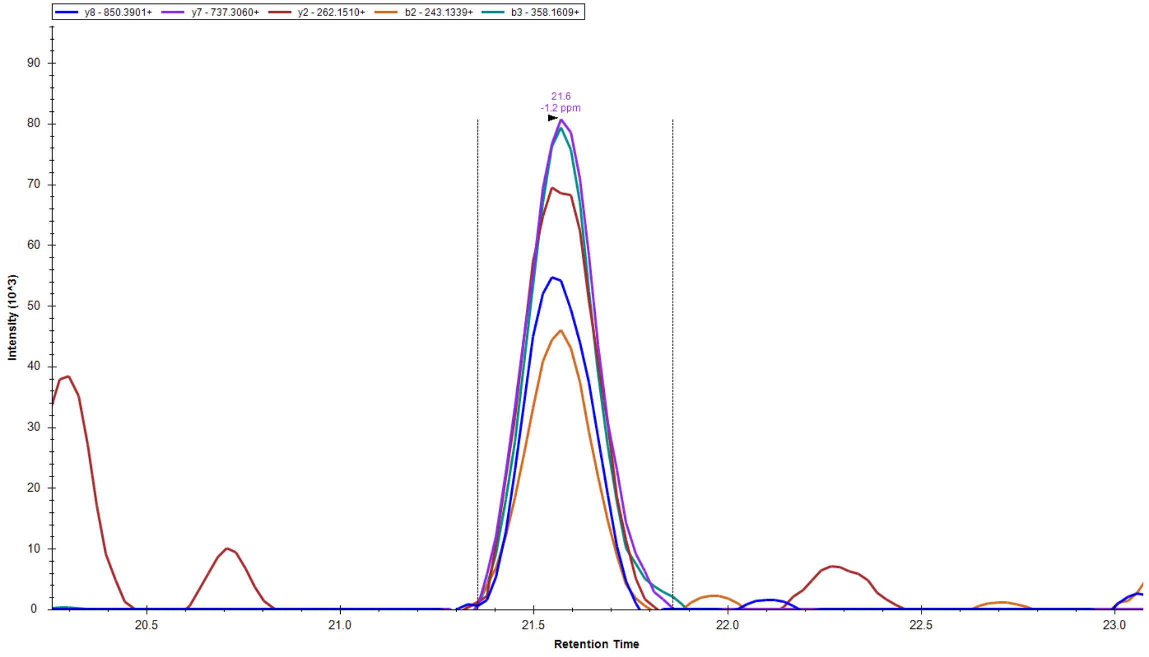

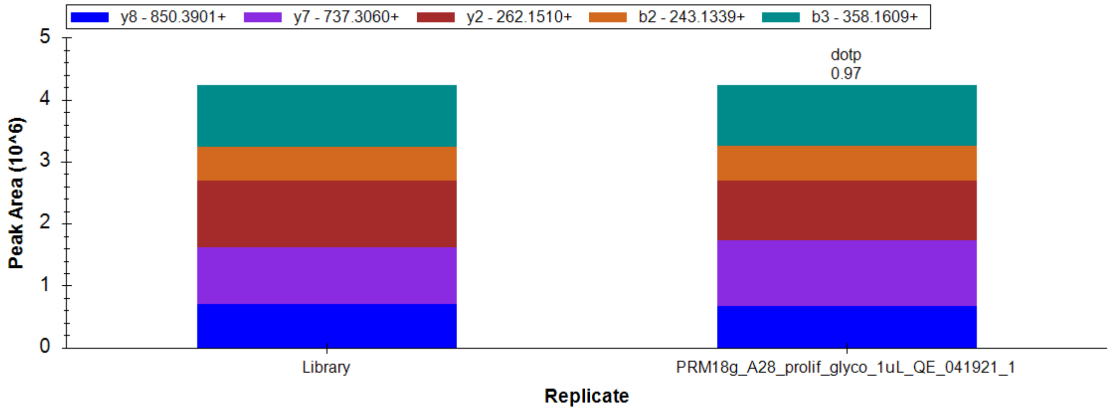

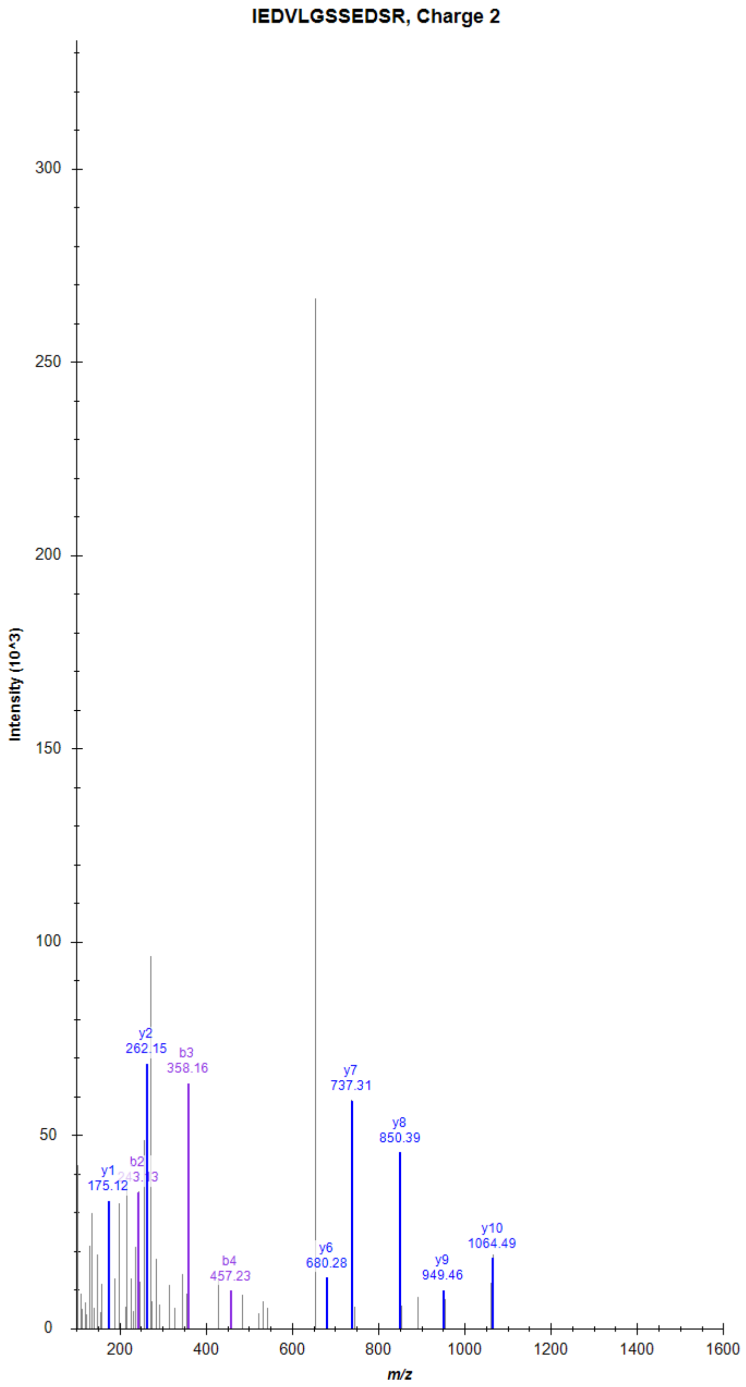

B2 bradykinin receptor (BDKRB2)

SEPIQMENSMGTLR, Charge 2, m/z = 796.8734

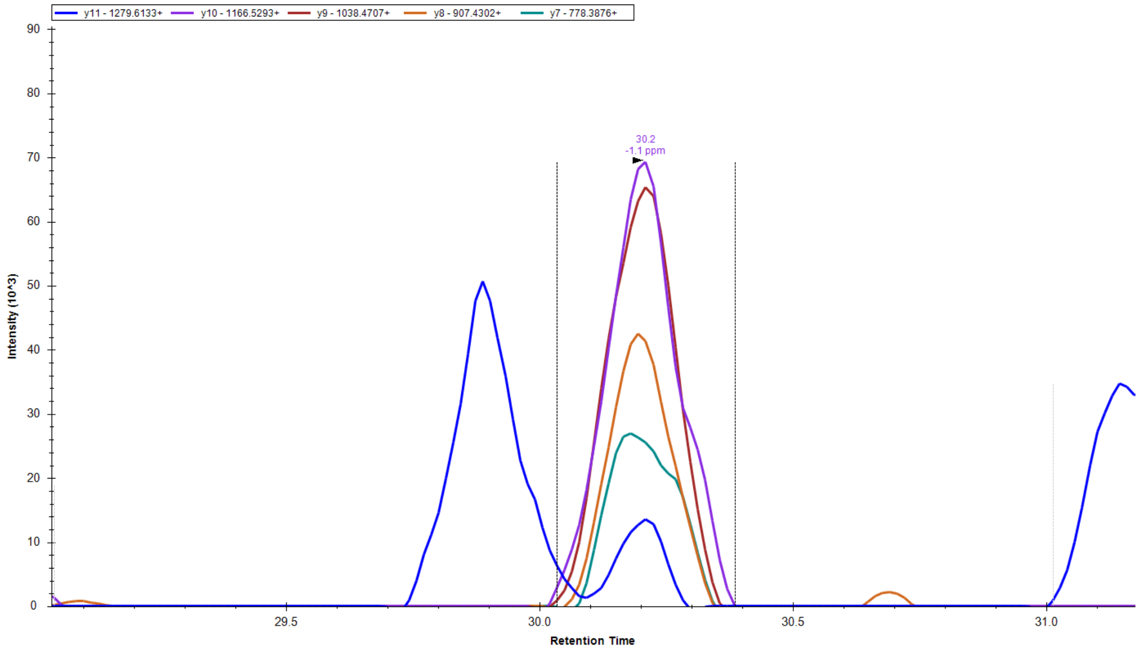

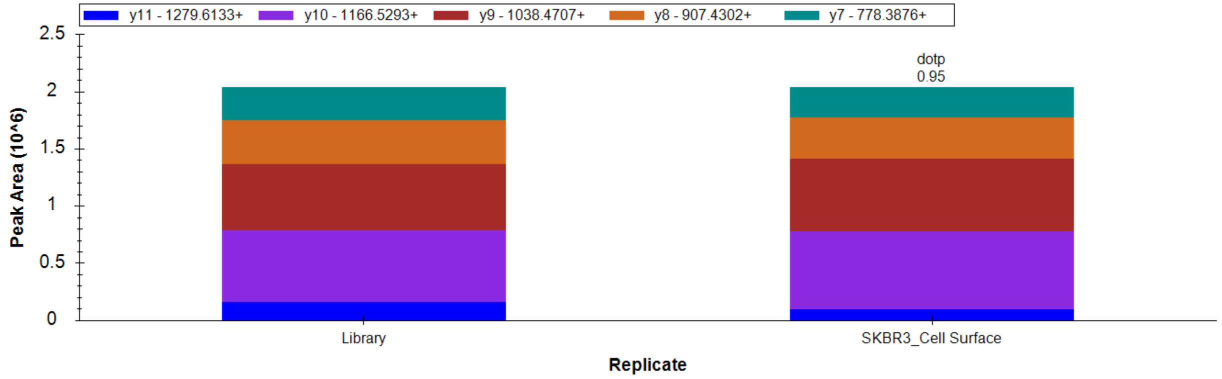

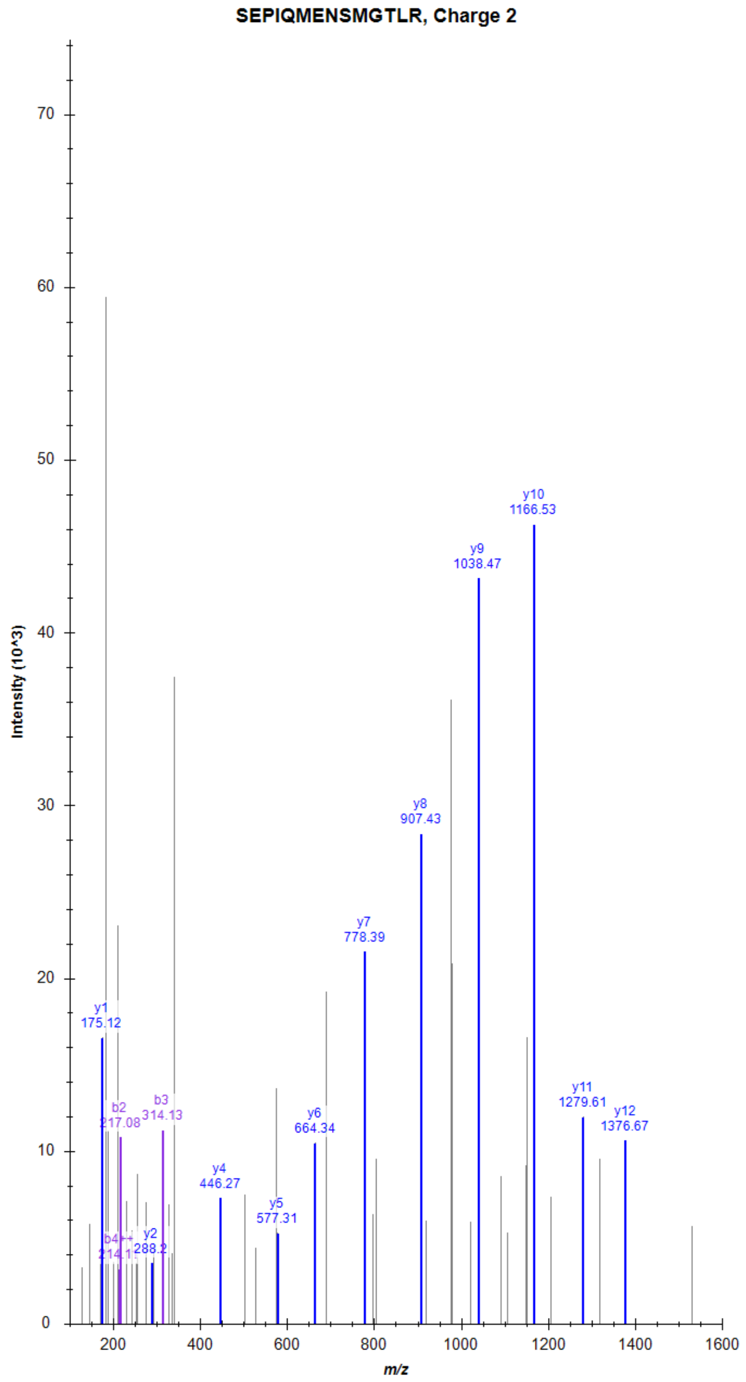

B2 bradykinin receptor (BDKRB2)

LQDWAGSR, Charge 2, m/z = 466.7322

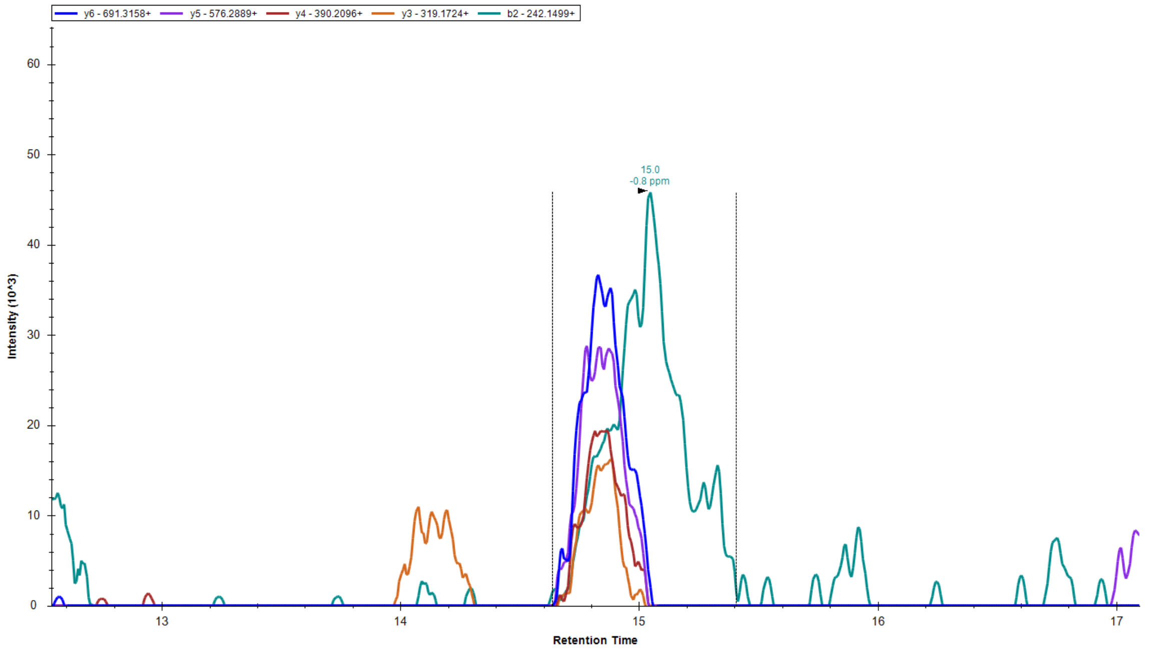

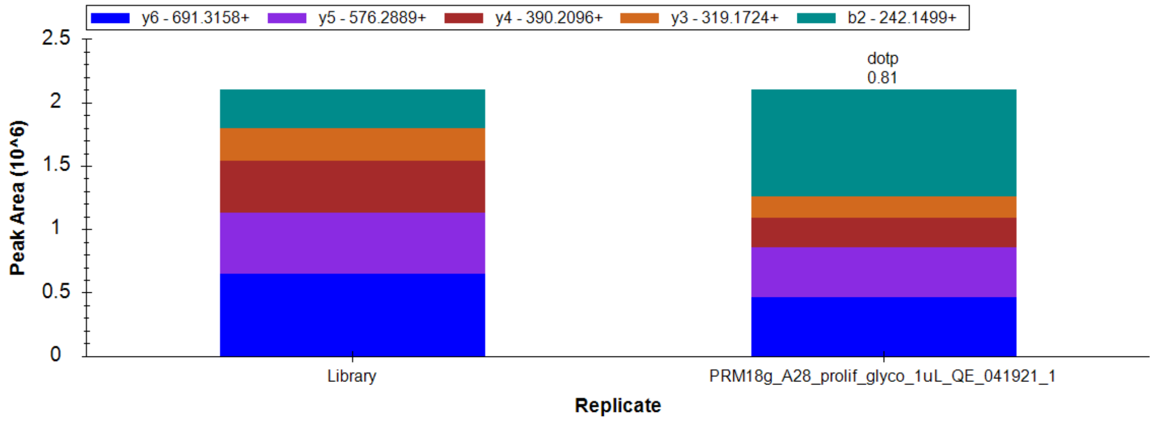

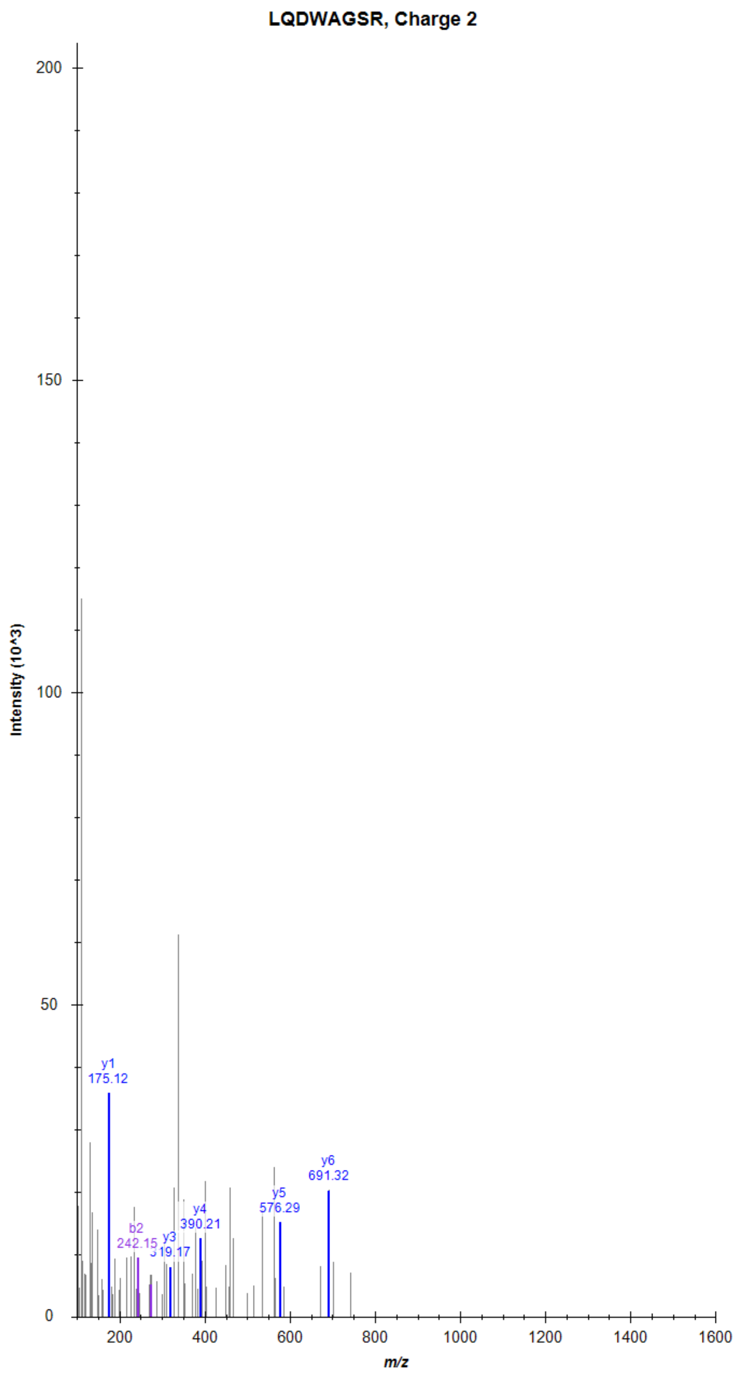

Frizzled-1 (FZD1)

VYGLMYFGPEELR, Charge 2, m/z = 787.3892

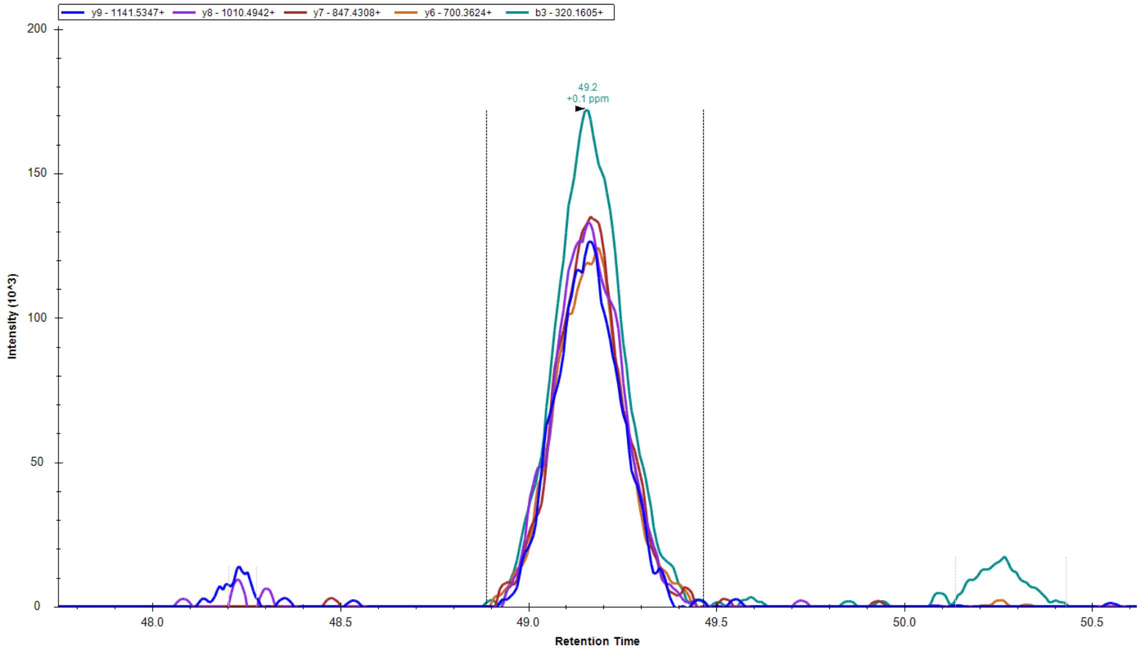

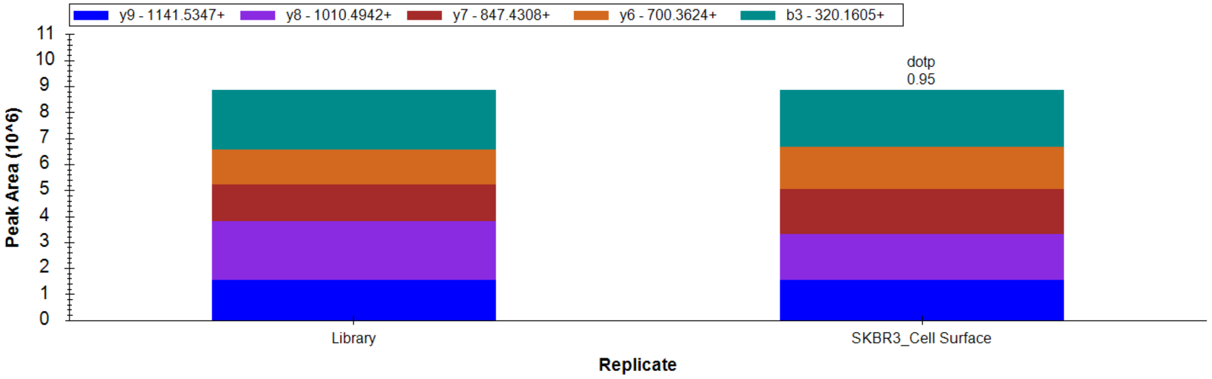

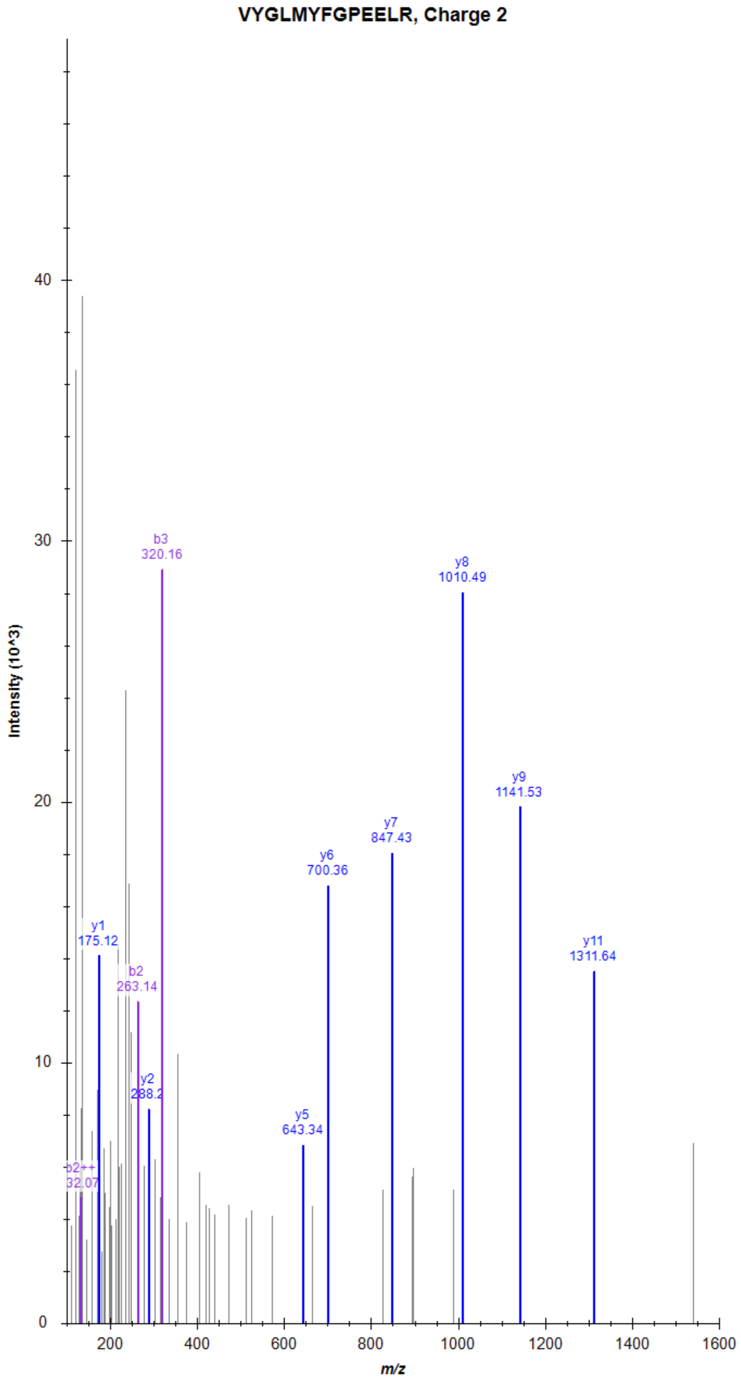

Cadherin EGF LAG seven-pass G-type receptor 2 (CELSR2)

SLDLTGPLLLGGVPDLPESFPVR, Charge 2, m/z = 1196.663

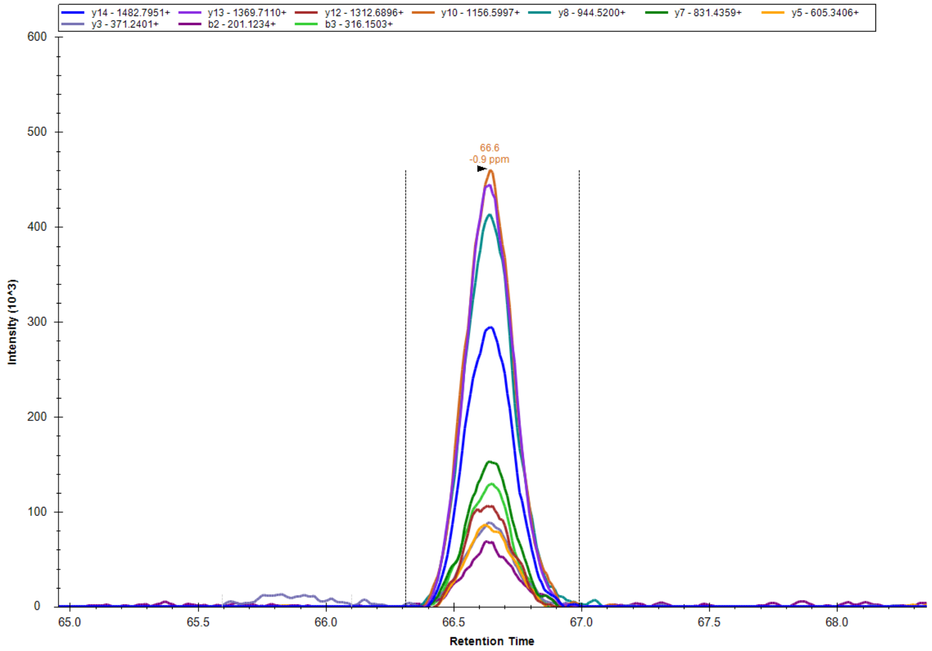

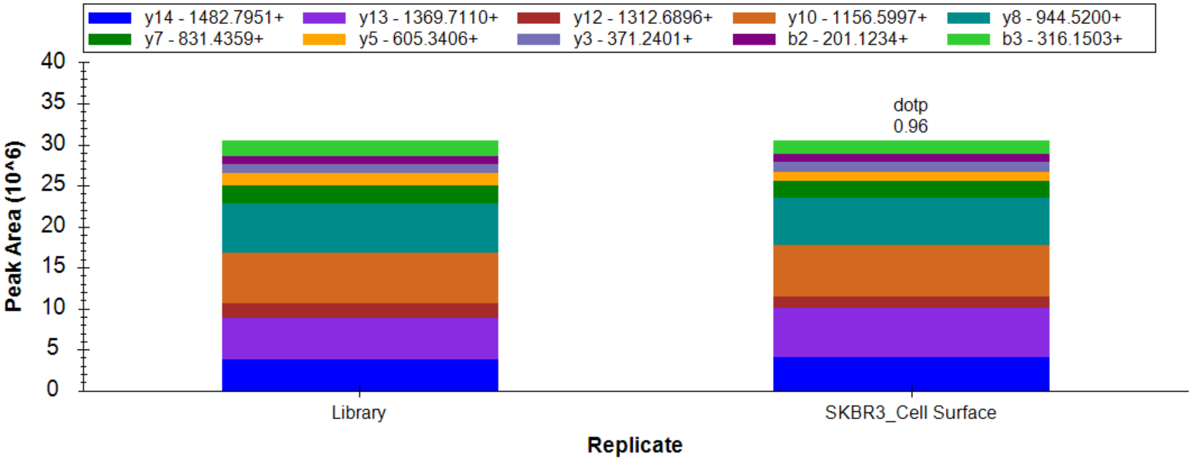

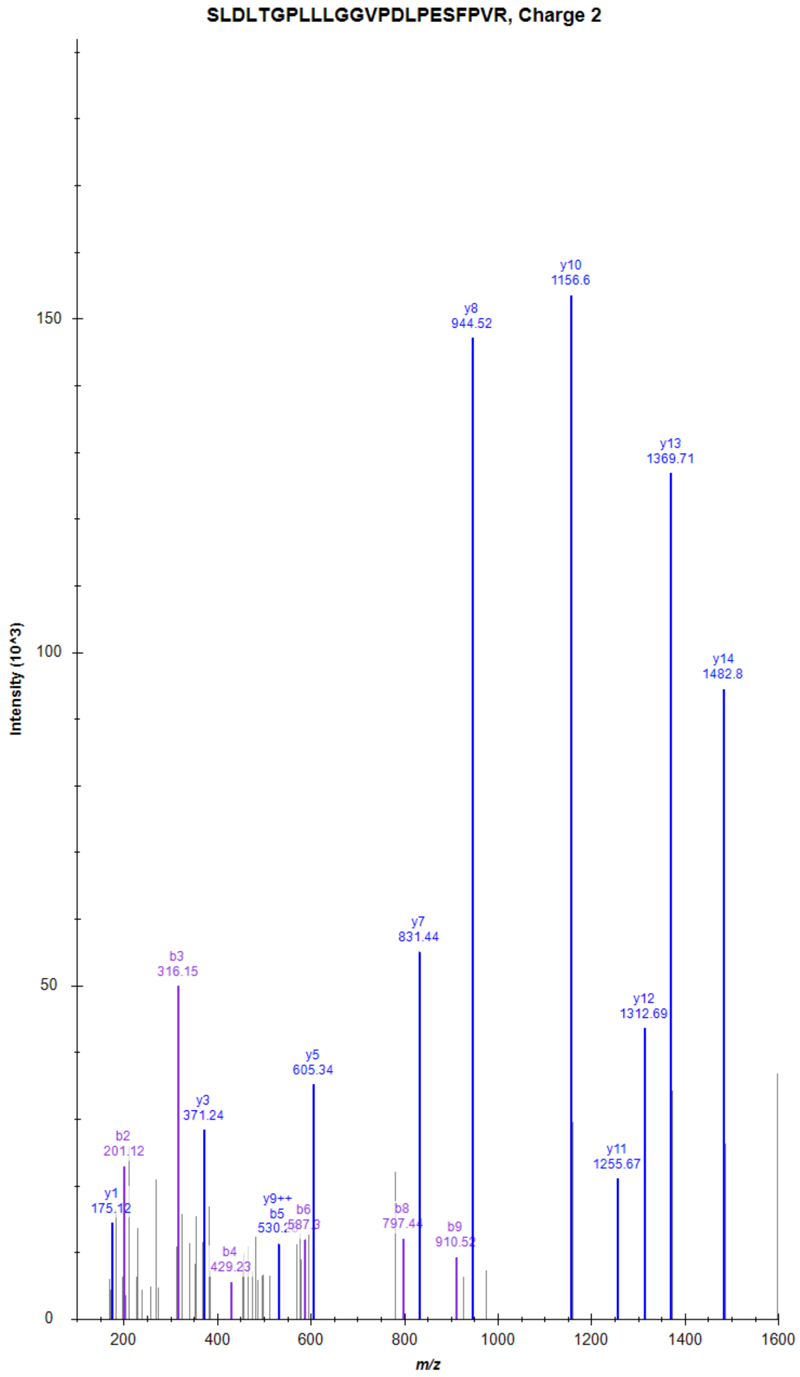

G-protein coupled receptor 39 (GPR39)

IFLSTFQSEAEPQSK, Charge 2, m/z = 856.4298

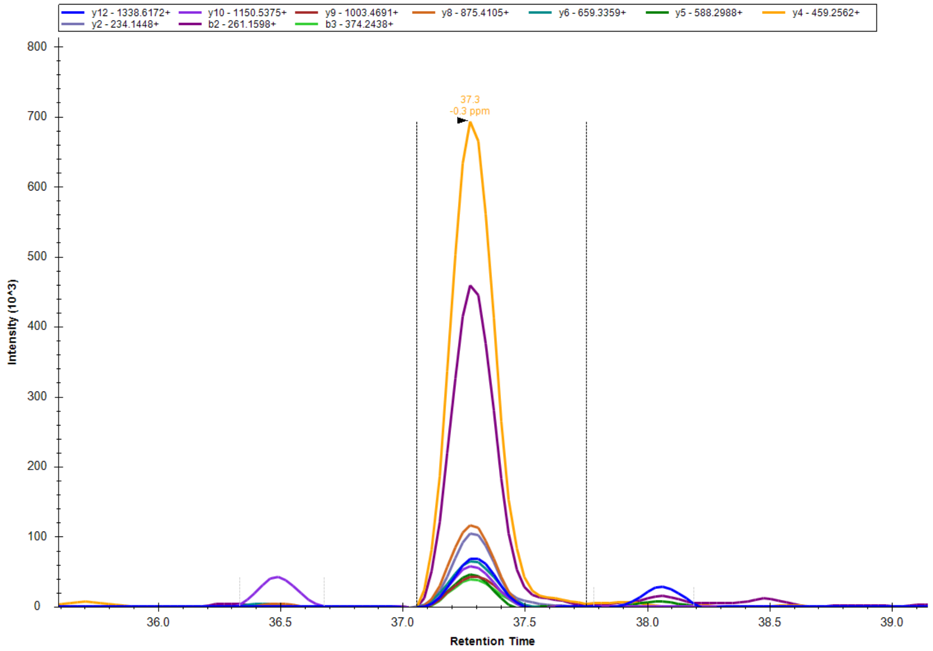

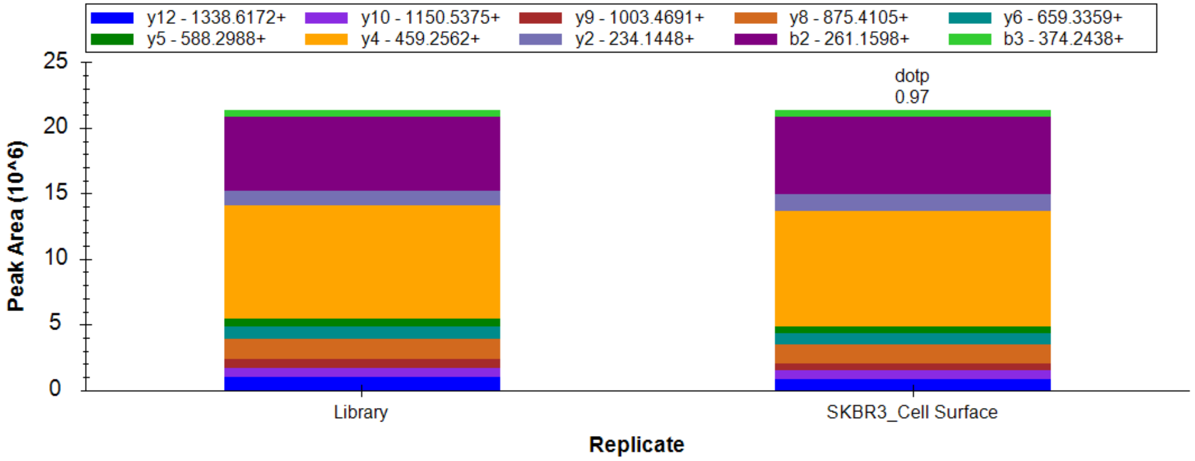

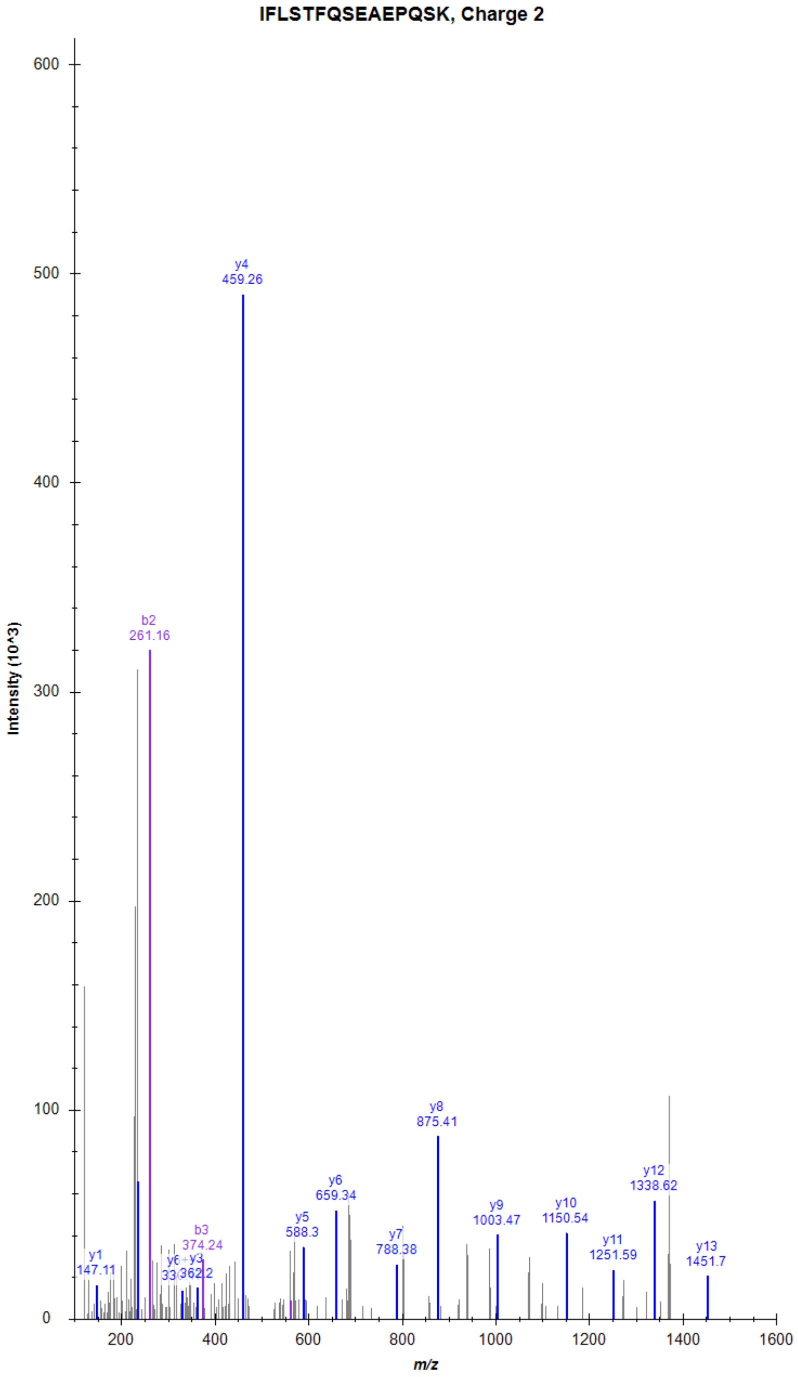

Supplement: Supplementary file 4 — Supplementary Information 4. [file 41598_2022_14418_MOESM4_ESM.pdf]
