# Supplementary material for: The SKBR3 cell-membrane proteome reveals telltales of aberrant cancer cell proliferation and targets for precision medicine applications
Source: Sci Rep. 2022 Jun 27;12:10847. doi: 10.1038/s41598-022-14418-0 (PMC9237123; doi:10.1038/s41598-022-14418-0)

The SKBR3 Cell-Membrane Proteome Reveals Telltales of Aberrant Cancer Cell Proliferation and Targets for Precision Medicine Applications

Arba Karcini and Iulia M. Lazar*

Virginia Tech, Blacksburg, VA

Parallel Reaction Monitoring (PRM) of selected peptides in cell-membrane cellular subfractions detected in SKBR3 by the glycoprotein enrichment method

Elevated in the serum-free (SF) cultured cells

ATP synthase subunit alpha (ATP5F1A)

TGAIVDVPVGEELLGR, Charge: +2, m/z = 812.94983 Da

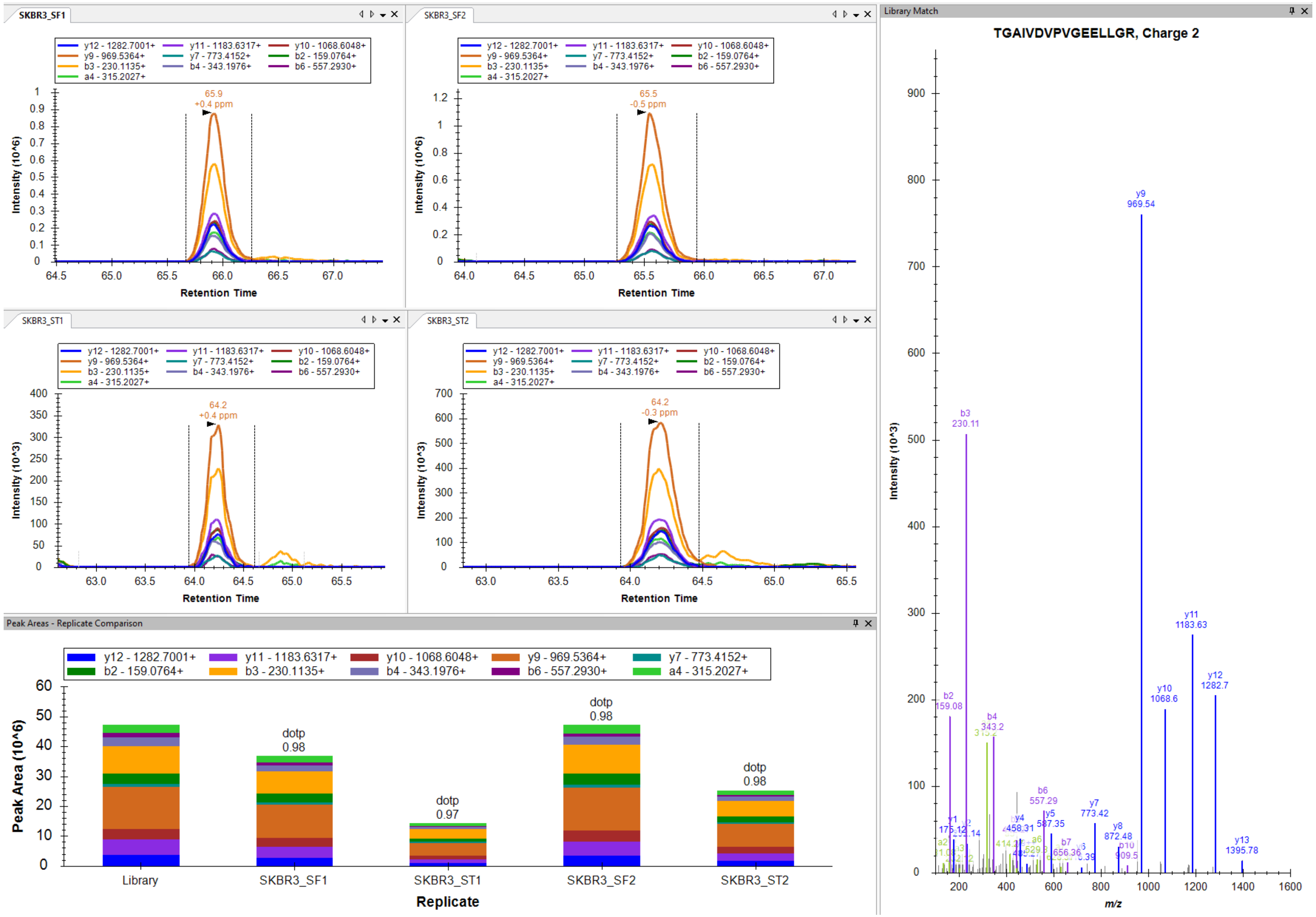

ATP synthase subunit alpha (ATP5F1A)

EAYPGDVFYLHSR, Charge: +2, m/z = 777.37512 Da

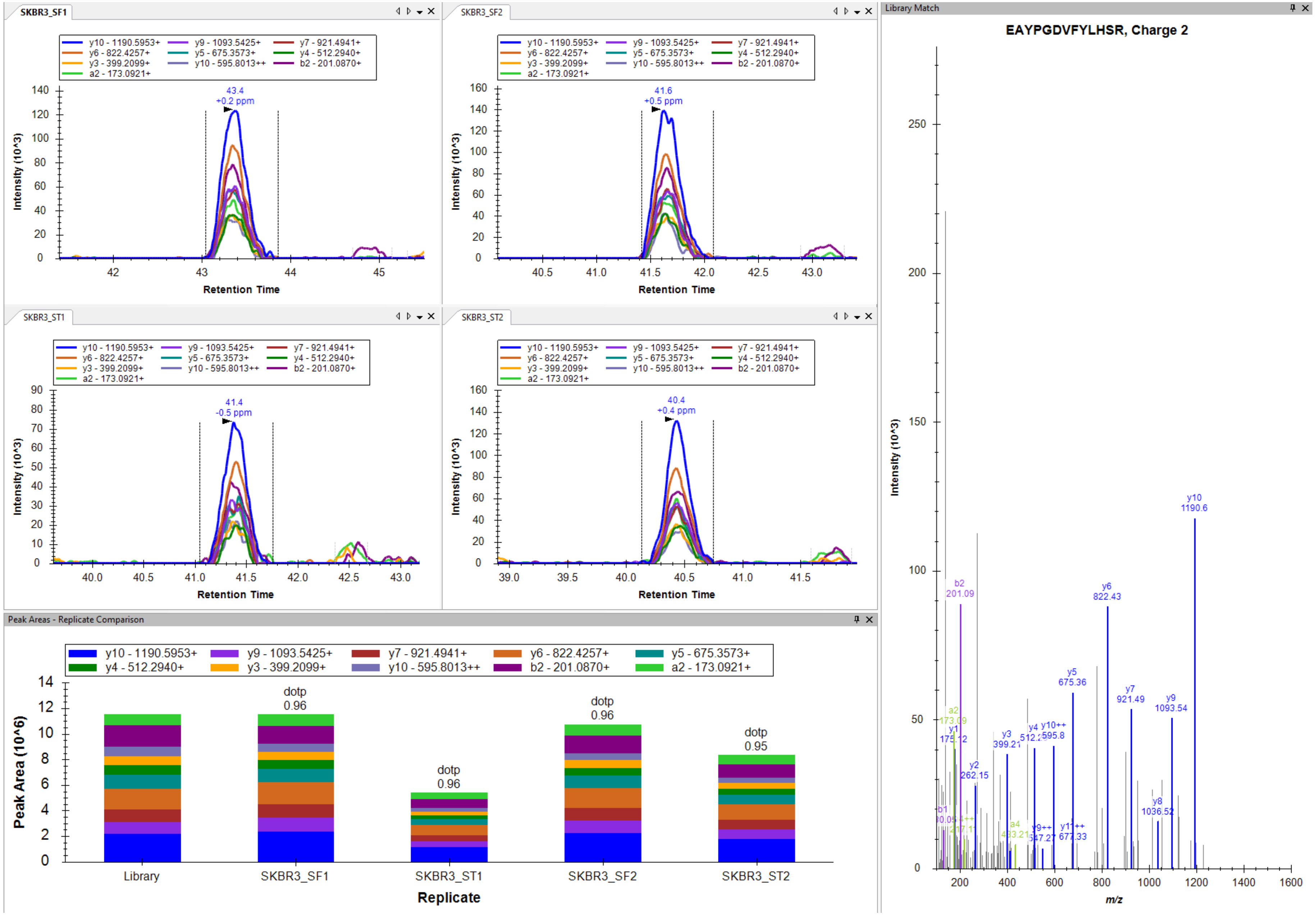

ATP synthase subunit beta (ATP5F1B)

DQEGQDVLLFIDNIFR, Charge: +3, m/z = 641.32819 Da

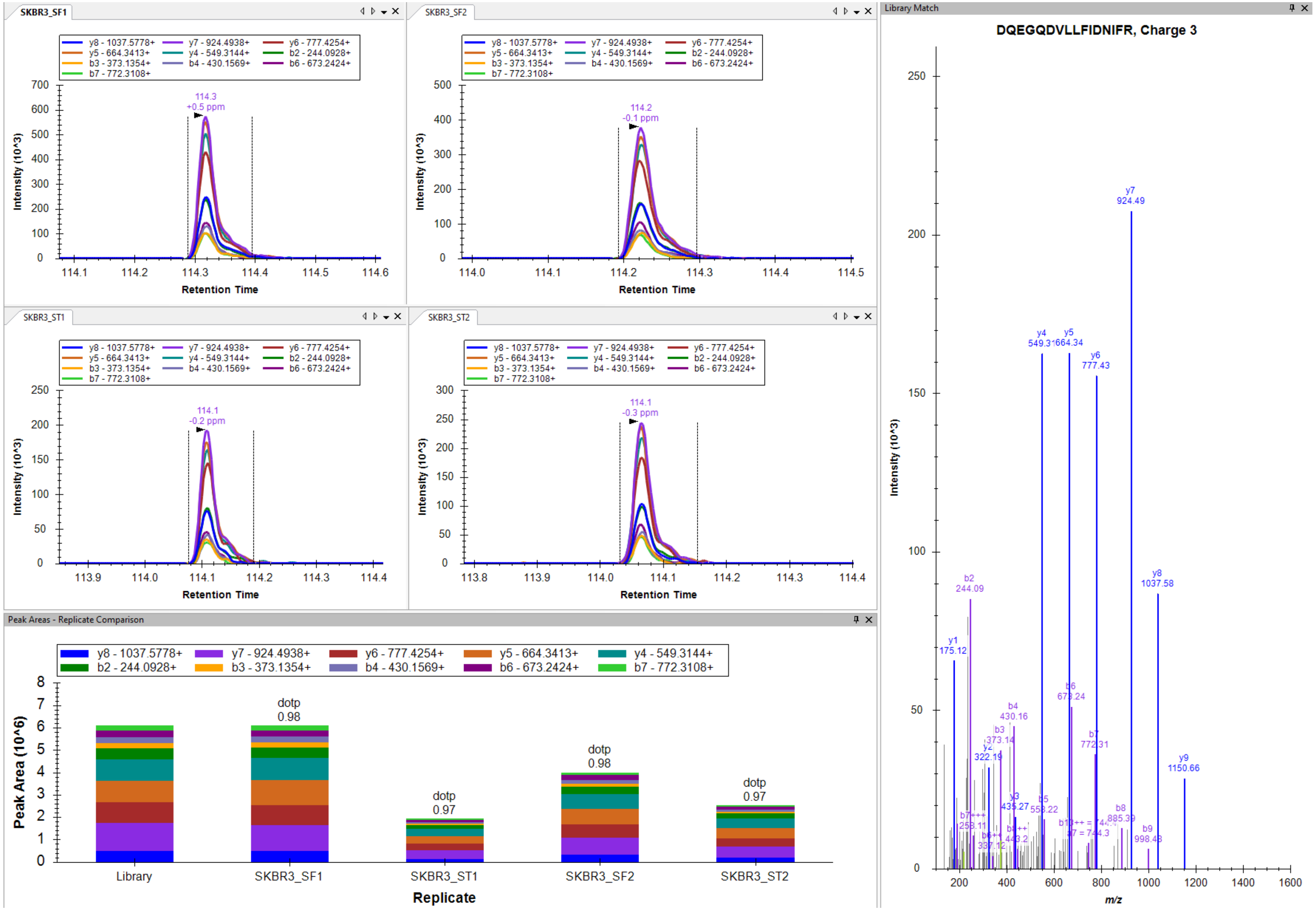

ATP synthase subunit beta (ATP5F1B)

AIAELGIYPAVDPLDSTSR, Charge: +2, m/z = 994.52026 Da

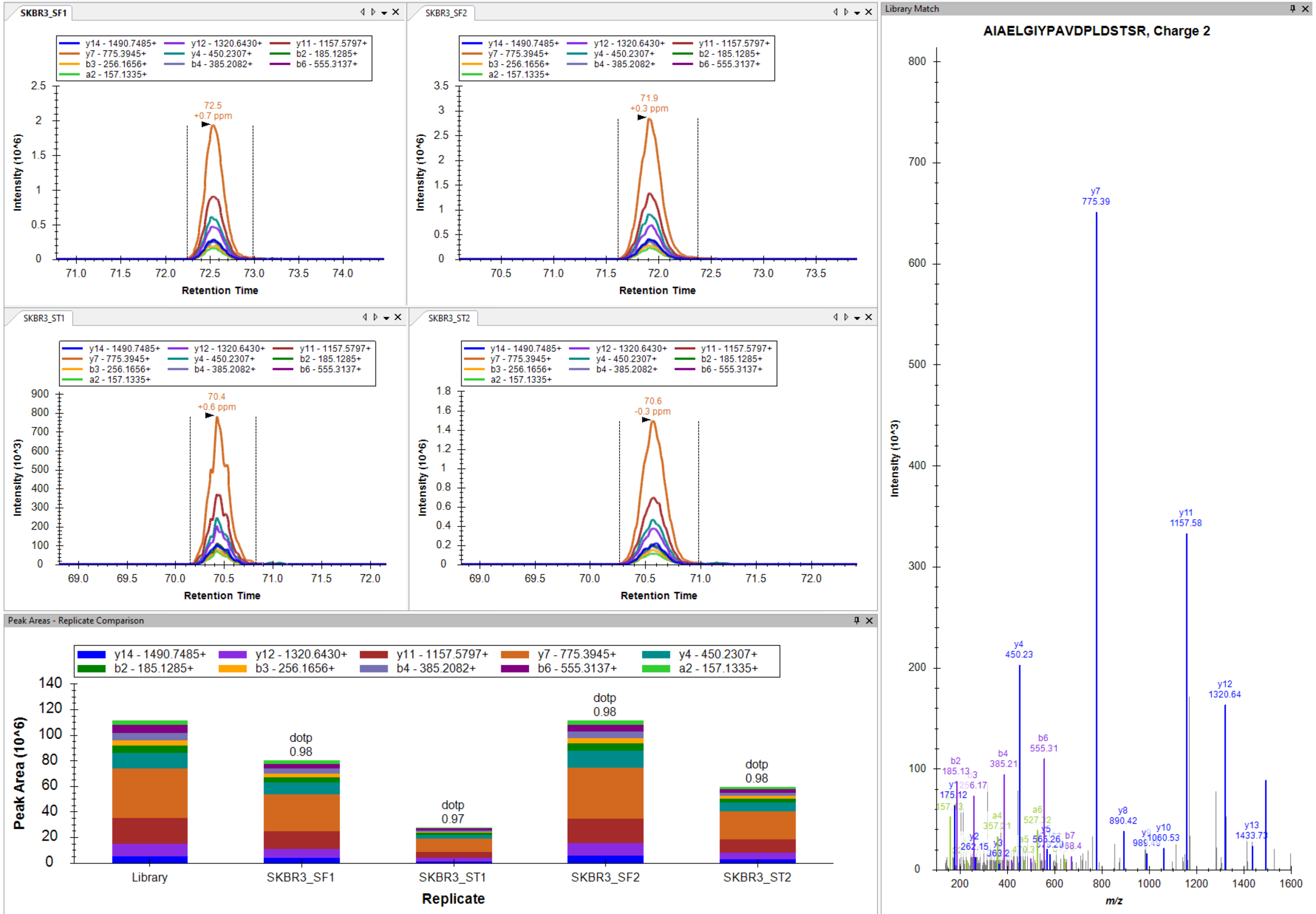

Cytochrome c oxidase subunit 5A (COX5A)

LNDFASTVR, Charge: +2, m/z = 511.76736 Da

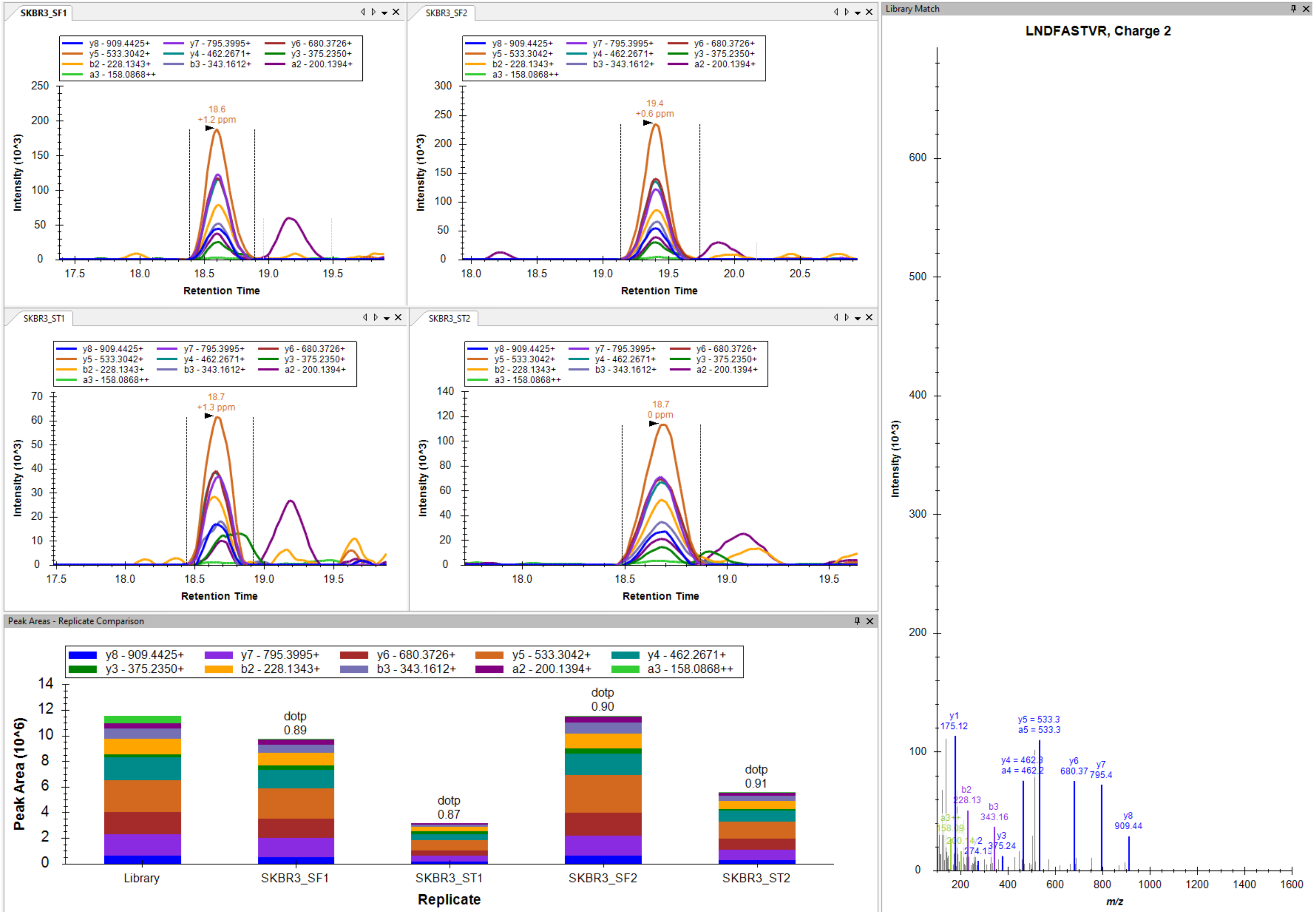

Cytochrome b-c1 complex subunit 1 (UQCRC1)

IAEVDASVVR, Charge: +2, m/z = 529.79755 Da

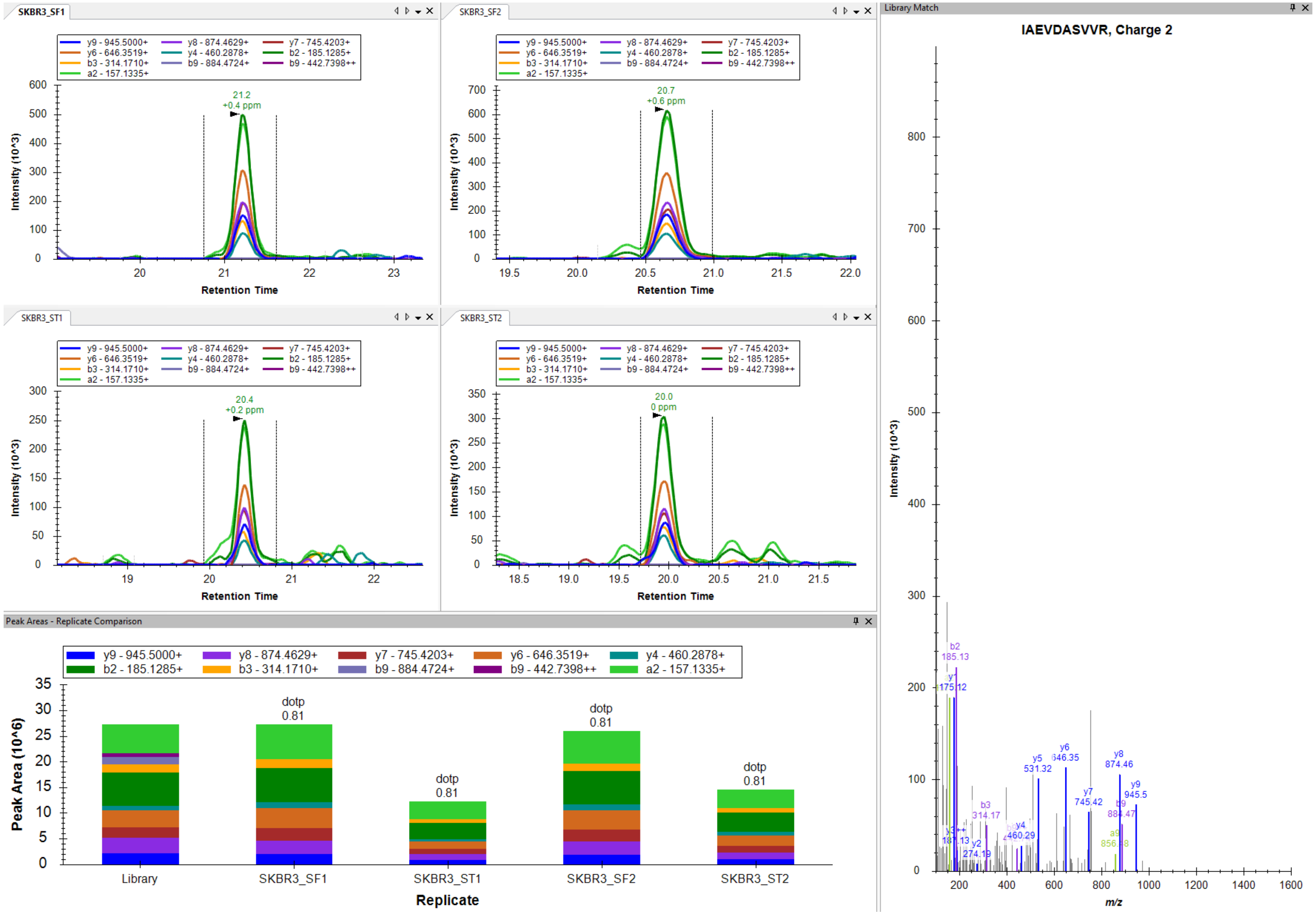

Cytochrome b-c1 complex subunit 2 (UQCRC2)

LPNGLVIASLENYSPVSR, Charge: +2, m/z = 965.02606 Da

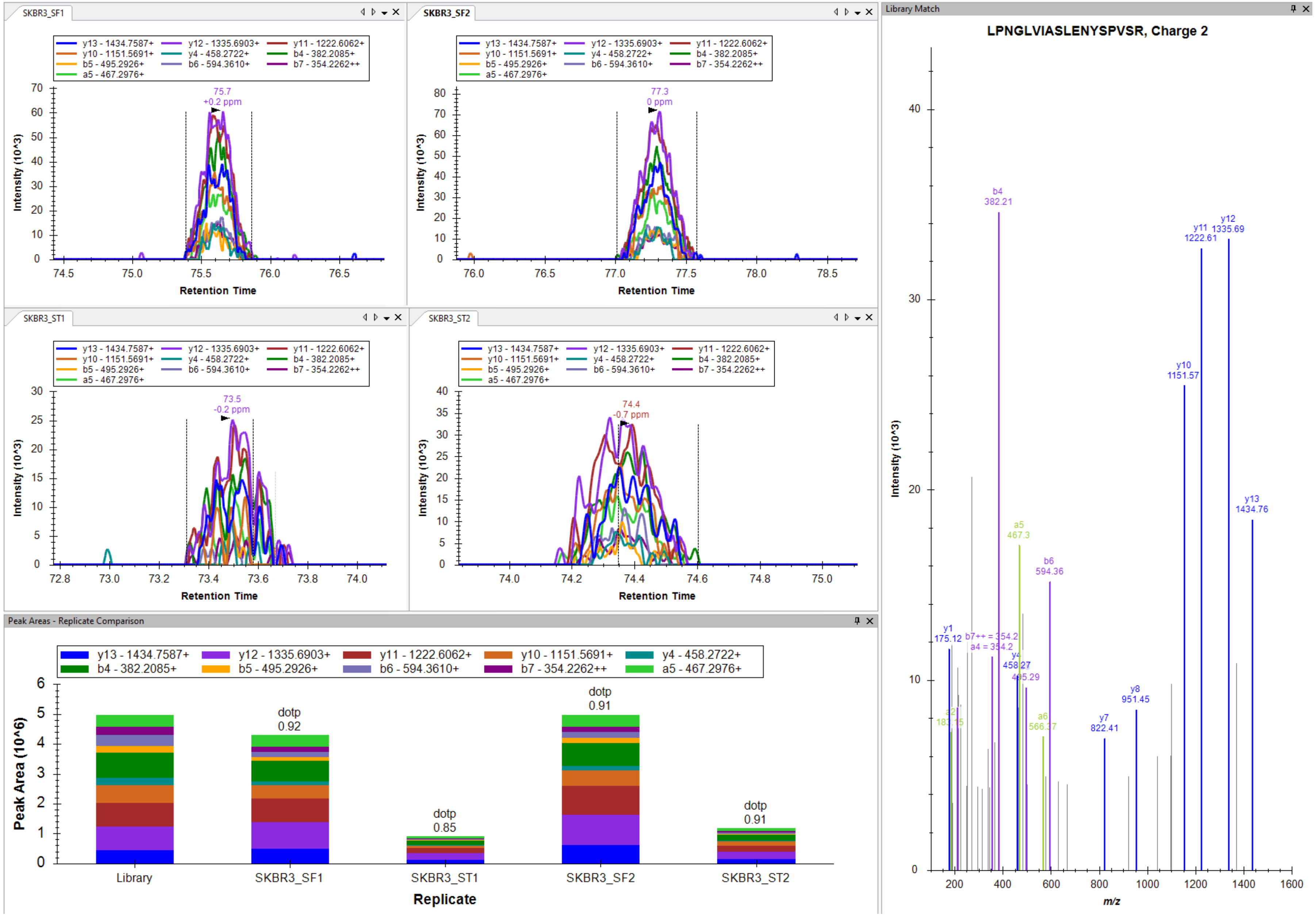

Succinate dehydrogenase [ubiquinone] flavoprotein subunit (SDHA)

LGANSLLDLVVFGR, Charge: +2, m/z = 737.42780 Da

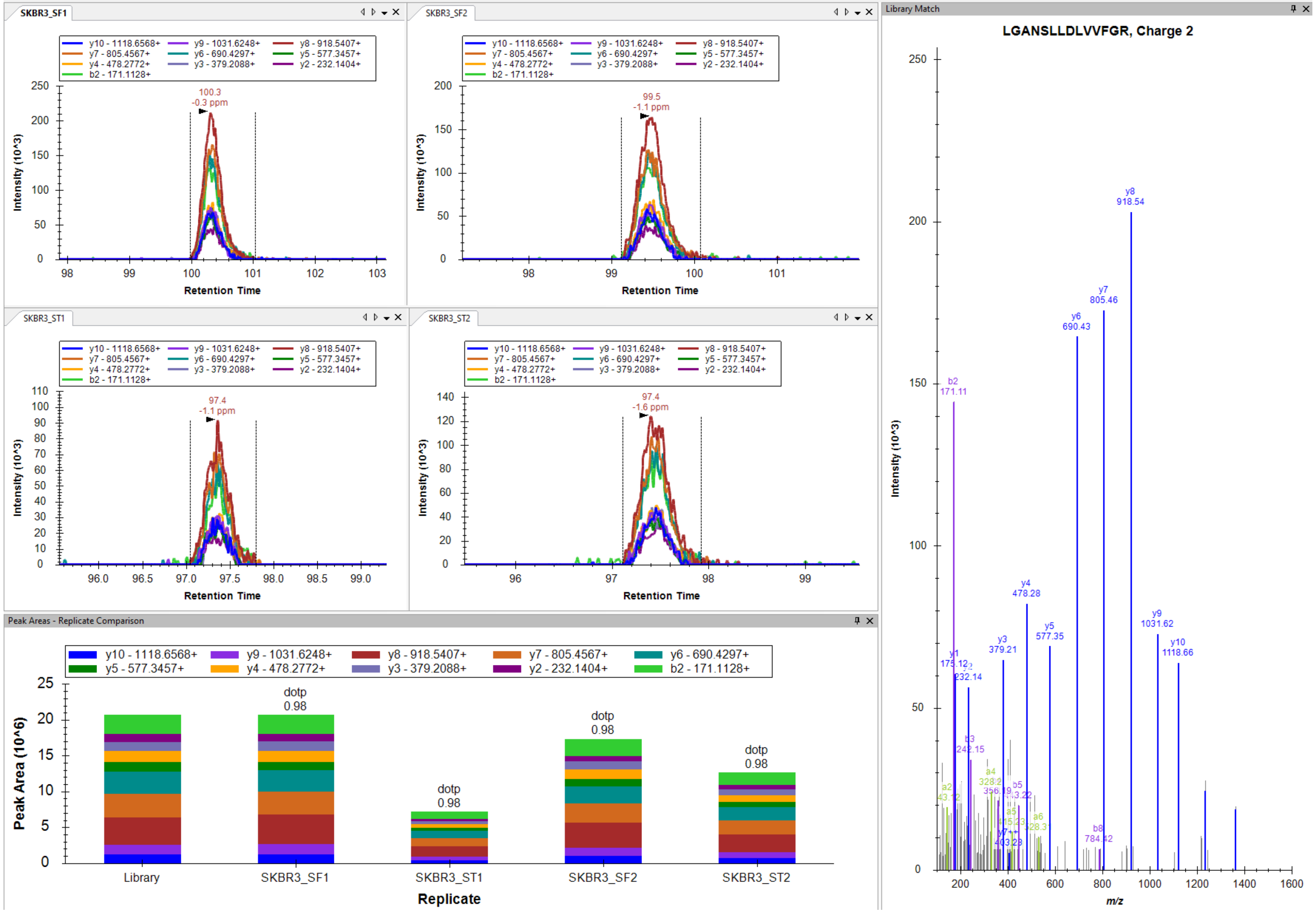

Stomatin-like protein 2 (STOML2)

ILEPGLNILIPVLDR, Charge: +2, m/z = 838.01489 Da

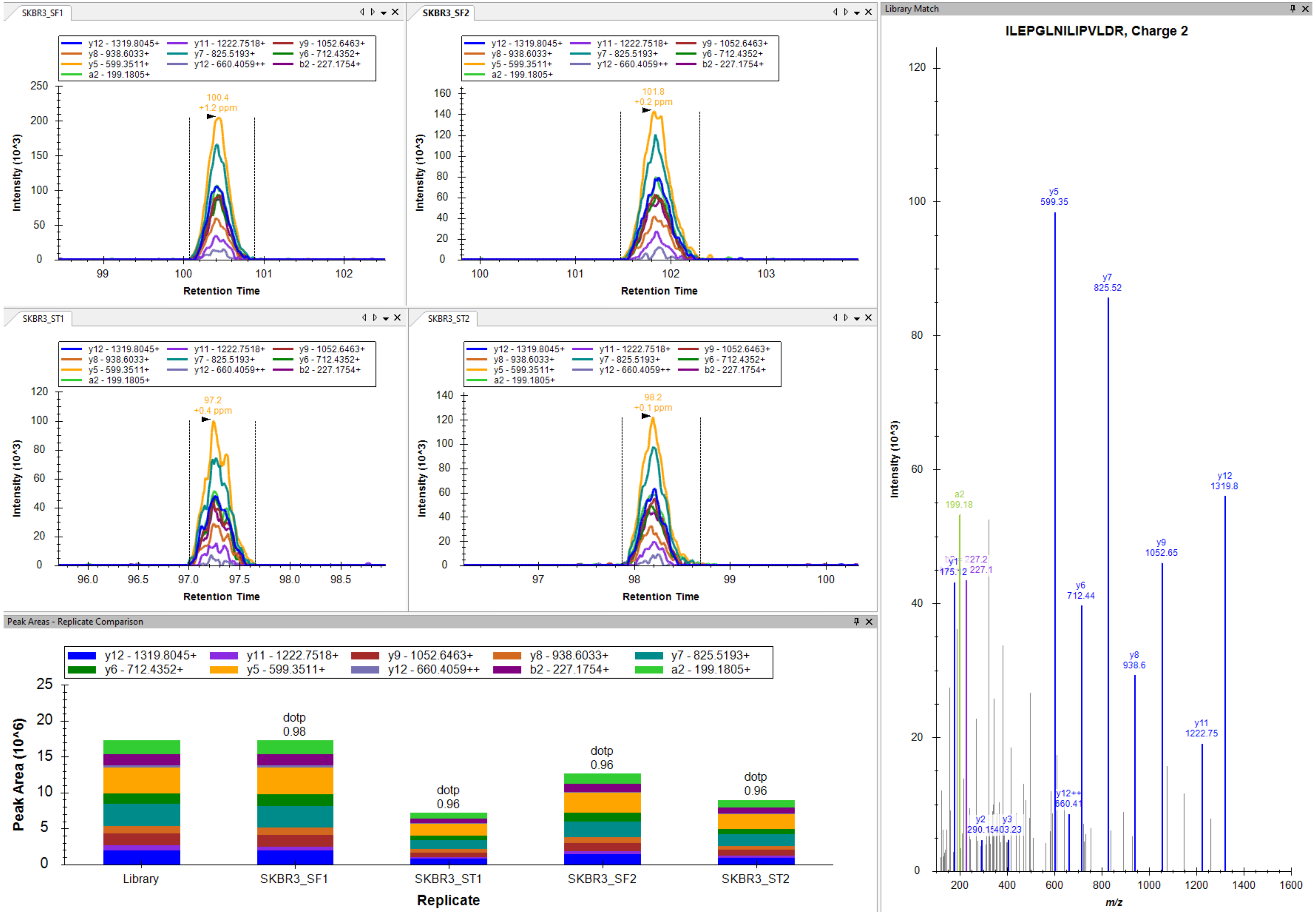

Elevated expression in the serum-treated (ST) cells

Hepatocyte growth factor receptor (MET)

TEFTTALQR, Charge: +2, m/z = 533.78027 Da

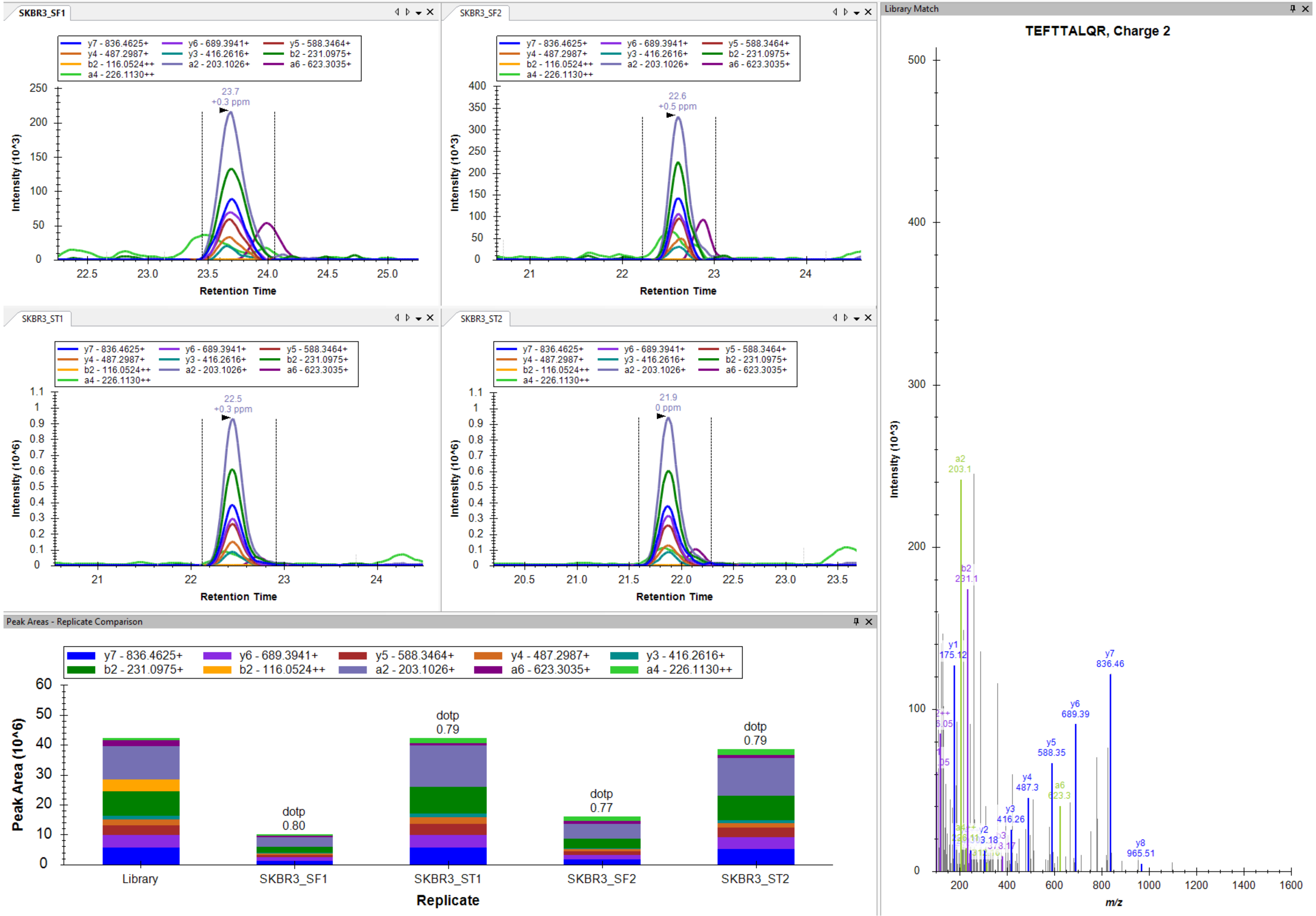

Hepatocyte growth factor receptor (MET)

ETLDAQTFHTR, Charge: +2, m/z = 659.82532 Da

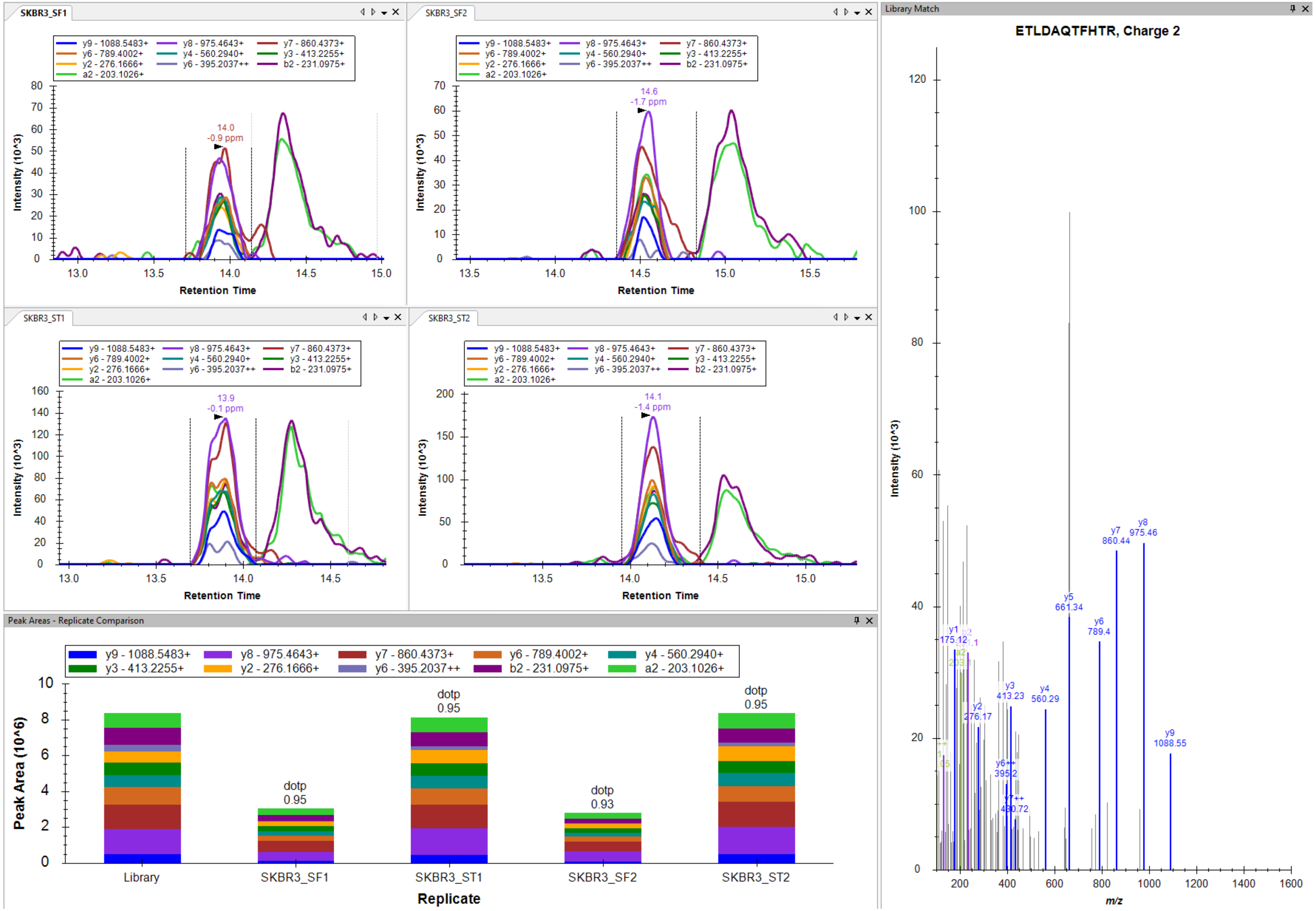

Insulin-like growth factor 1 receptor (IGF1R)

QPQDGYLYR, Charge: +2, m/z = 570.27856 Da

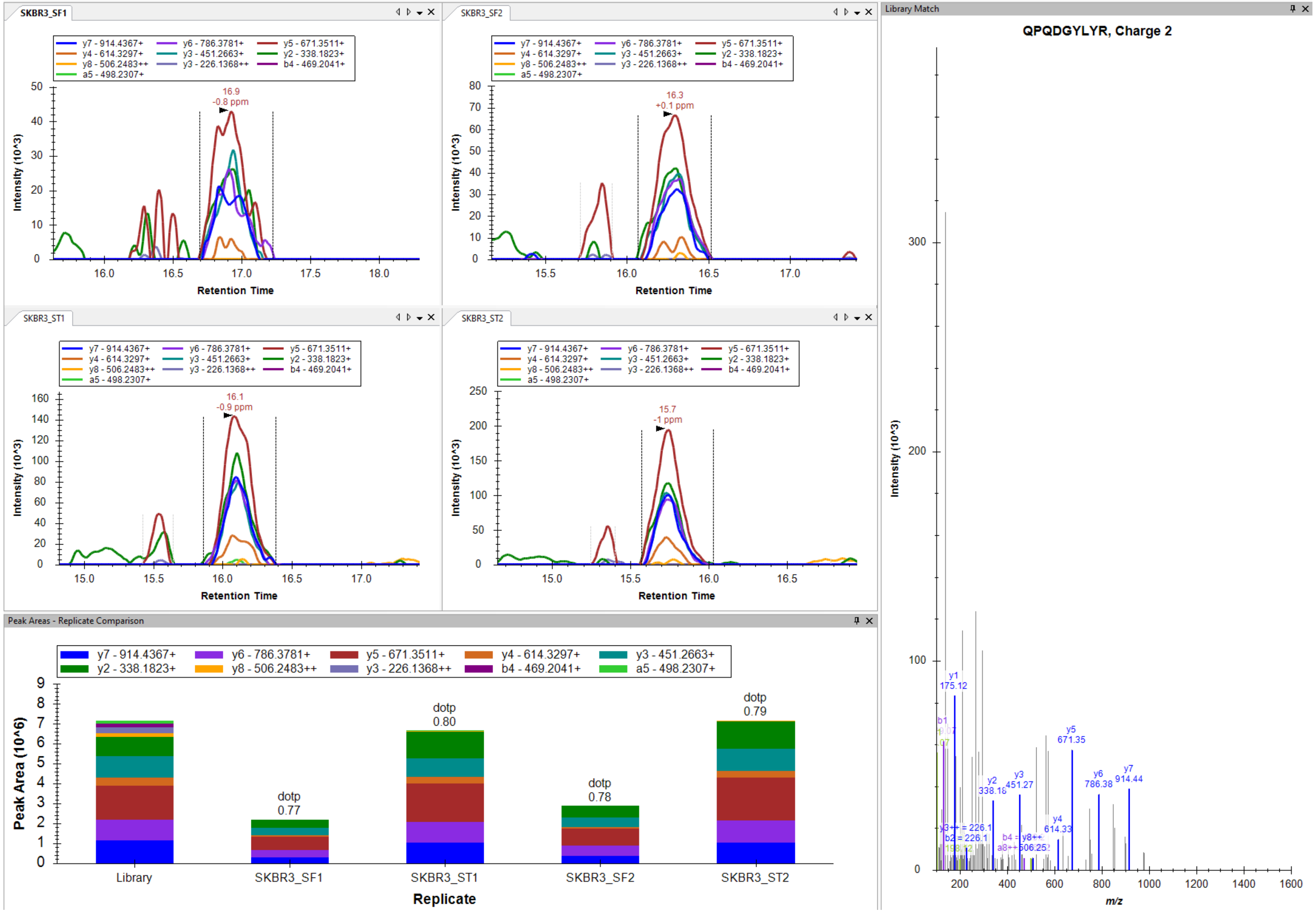

Insulin-like growth factor 1 receptor (IGF1R)

VAGLESLGDLFPNLTVIR, Charge: +2, m/z = 957.54065 Da

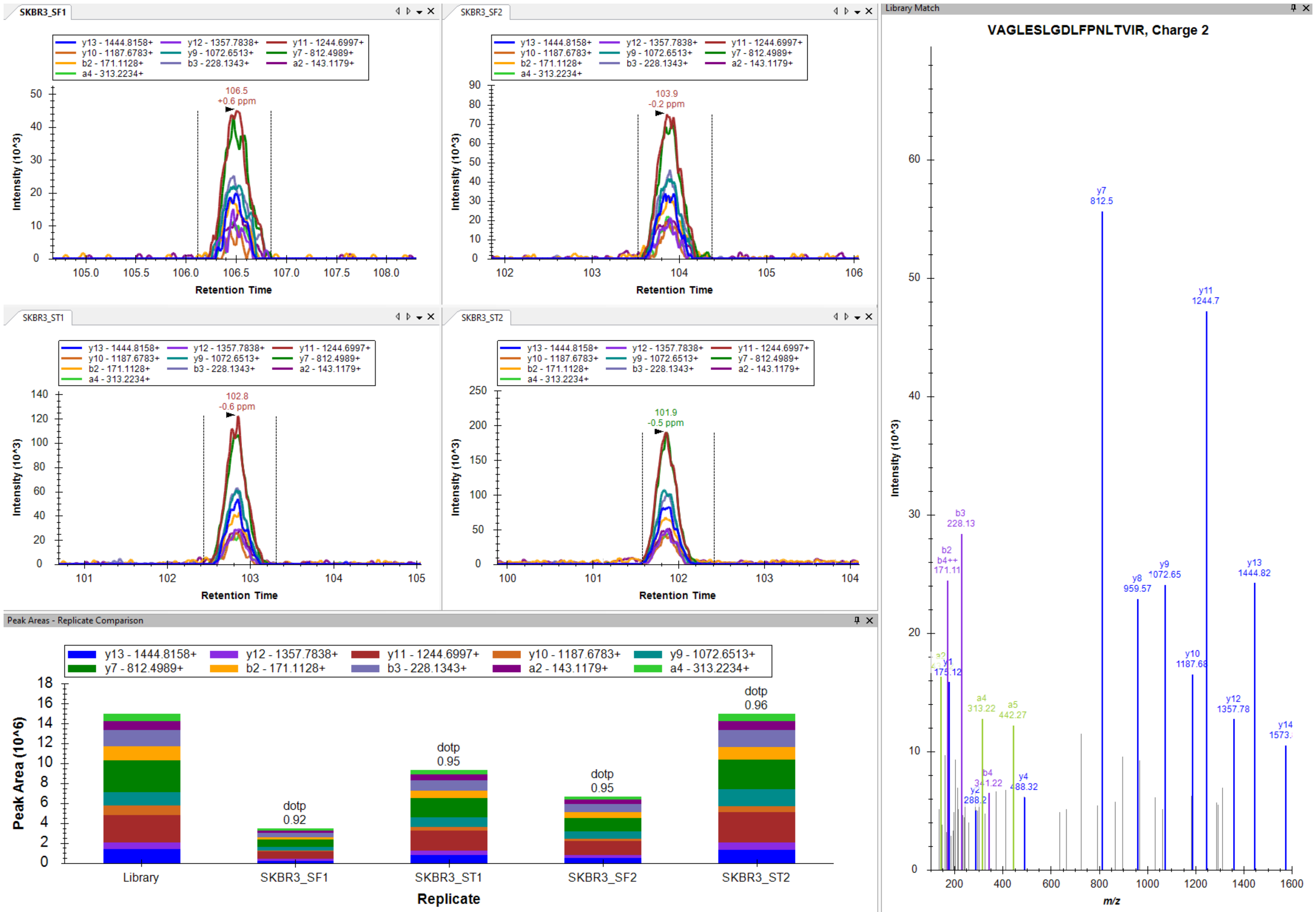

CD44 antigen (CD44)

LVINSGNGAVEDR, Charge: +2, m/z = 672.34949 Da

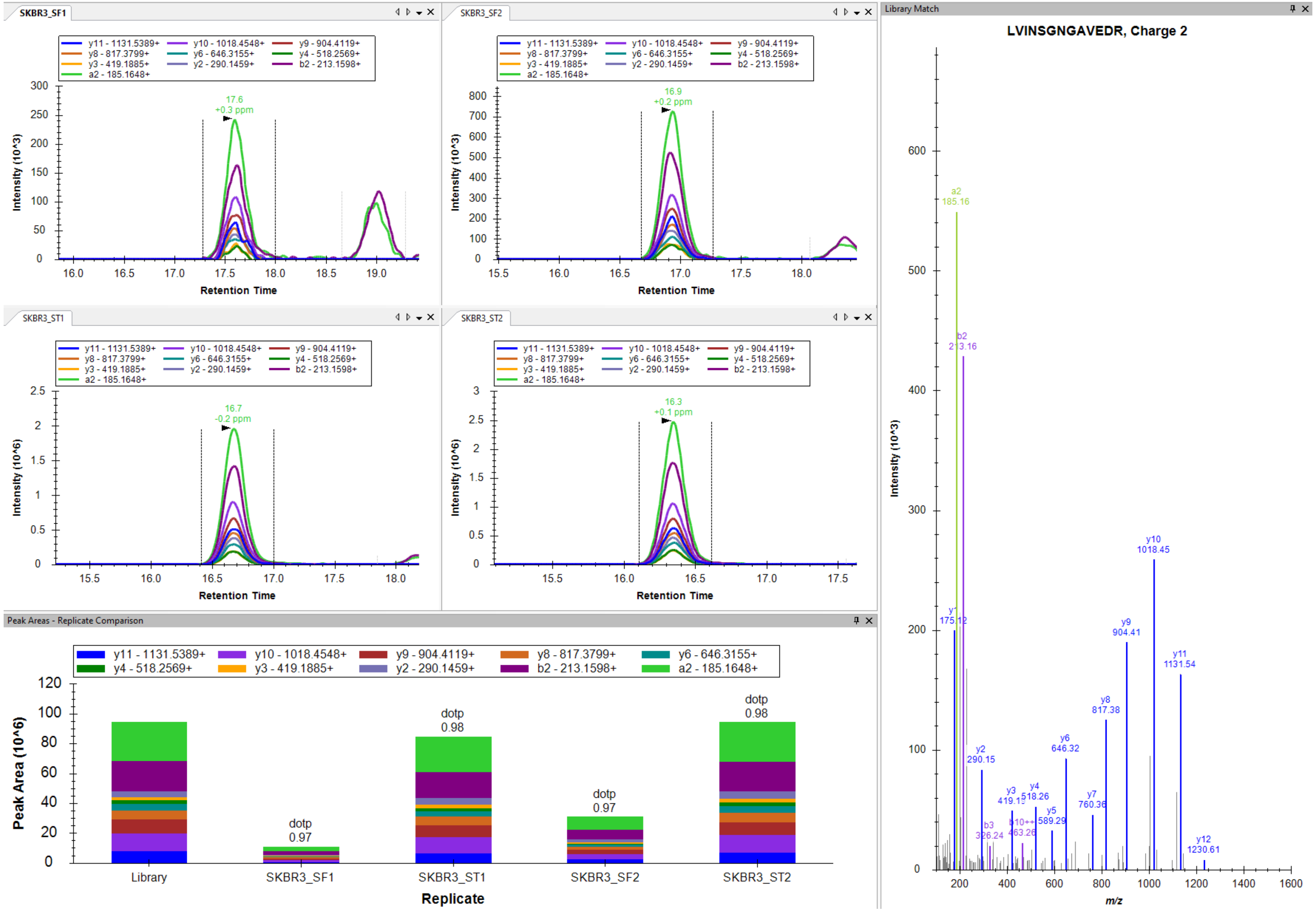

CD44 antigen (CD44)

ESSETPDQFMTADETR, Charge: +2, m/z = 922.38654 Da

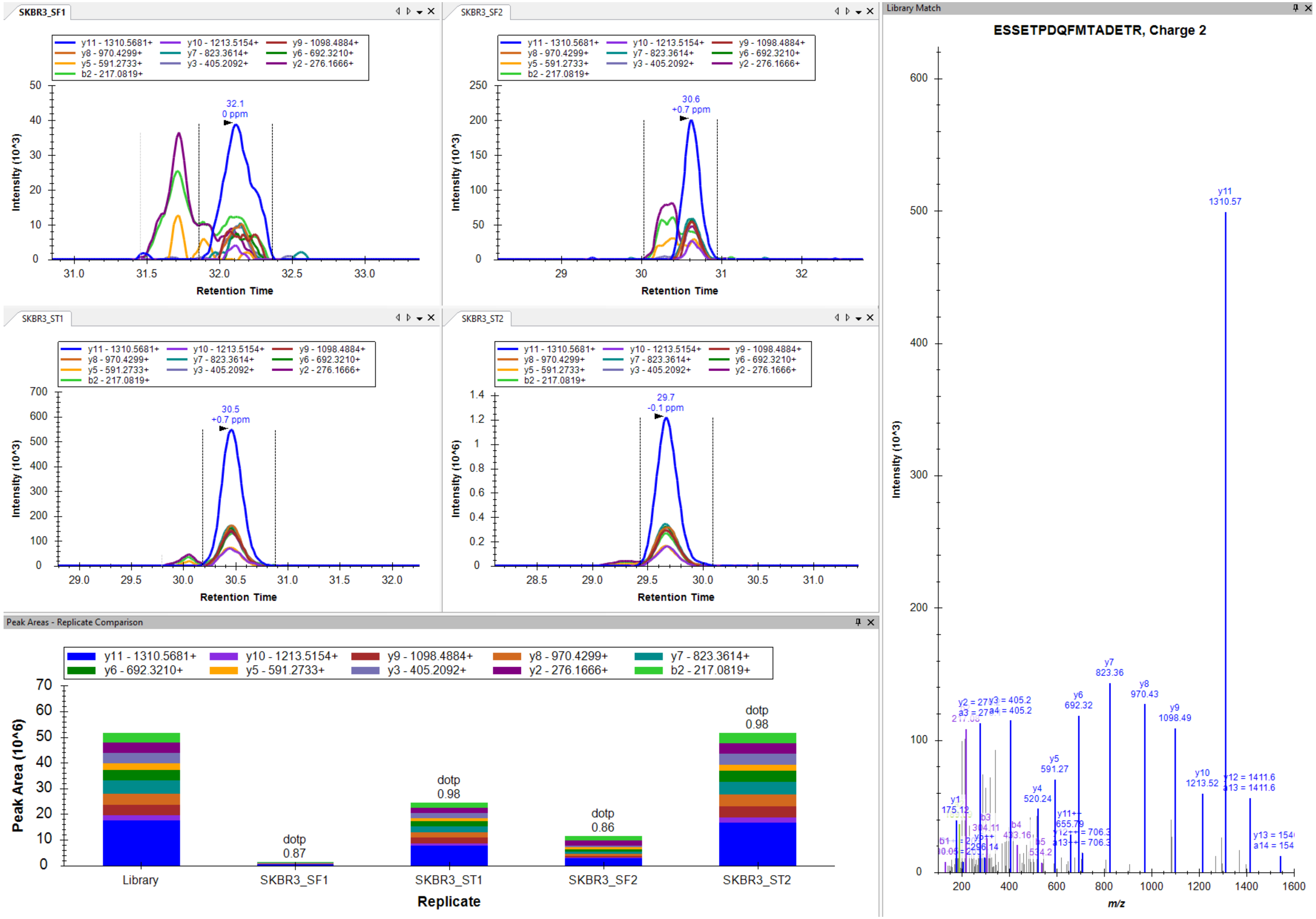

CD44 antigen (CD44)

SQEMVHLVNK, Charge: +2, m/z = 592.80890 Da

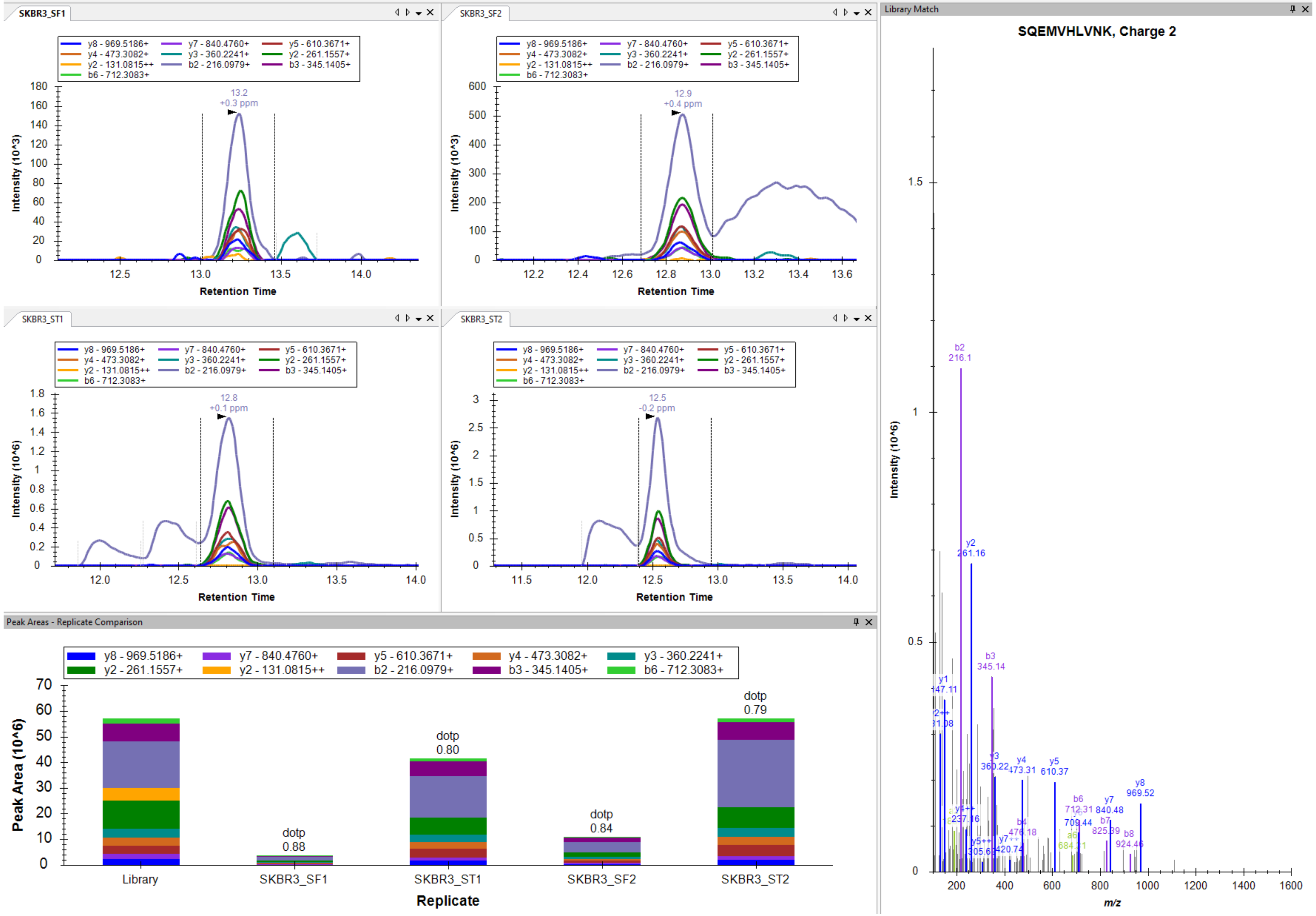

P2Y purinoceptor 2 (P2RY2)

TLYYSFR, Charge: +2, m/z = 475.24411 Da

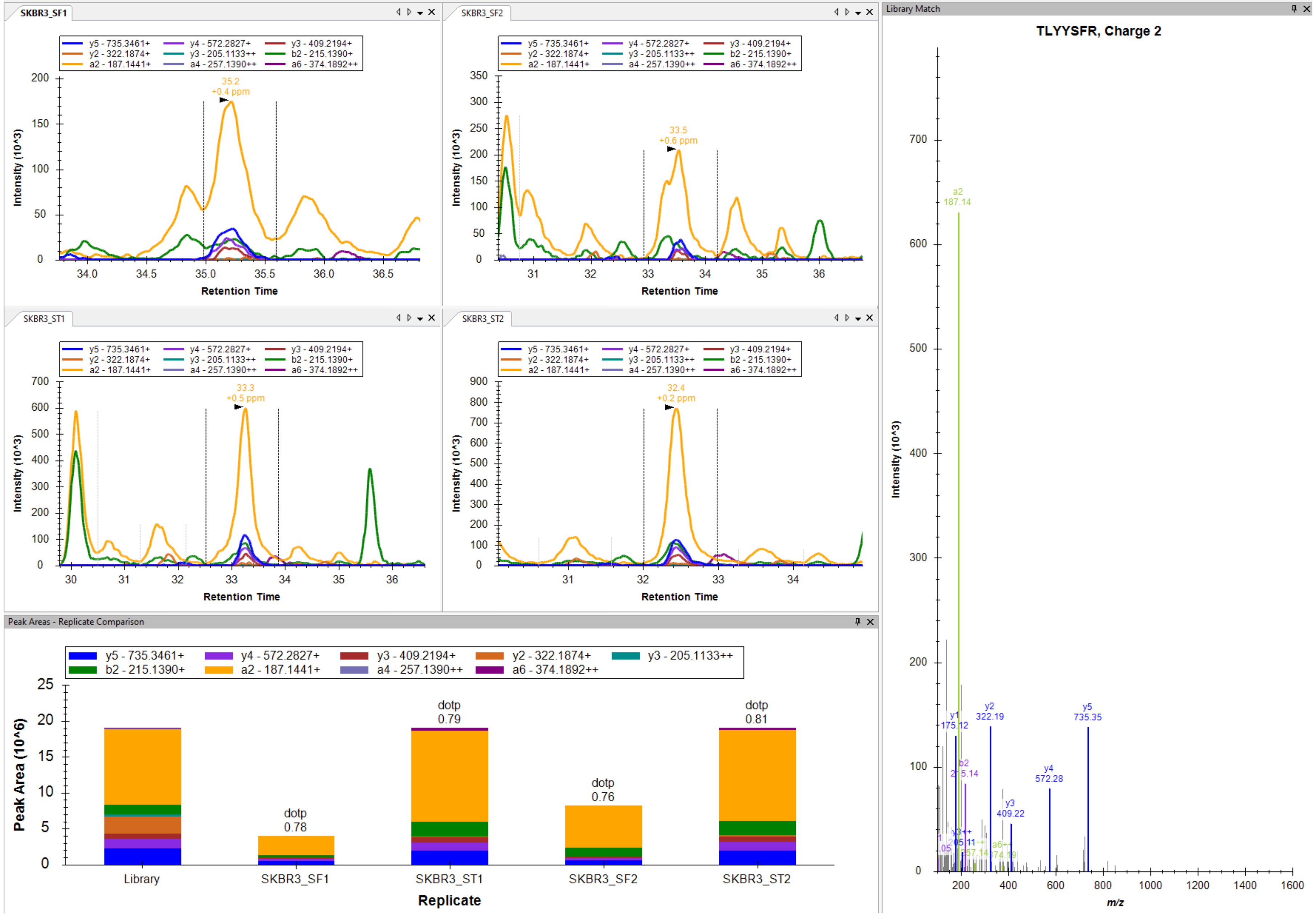

Supplement: Supplementary file 7 — Supplementary Information 7. [file 41598_2022_14418_MOESM7_ESM.pdf]
