# Supplementary material for: The SKBR3 cell-membrane proteome reveals telltales of aberrant cancer cell proliferation and targets for precision medicine applications
Source: Sci Rep. 2022 Jun 27;12:10847. doi: 10.1038/s41598-022-14418-0 (PMC9237123; doi:10.1038/s41598-022-14418-0)
Supplement: Supplementary file 8 — Supplementary Information 8. [file 41598_2022_14418_MOESM8_ESM.pdf]

The SKBR3 Cell-Membrane Proteome Reveals Telltales of Aberrant Cancer Cell Proliferation and Targets for Precision Medicine Applications

Arba Karcini and Iulia M. Lazar*

Virginia Tech, Blacksburg, VA

ATP5FA immunofluorescence visualization (w. cell permeabilization)

SKBR3 cells
Cell fixation: PFA (2 %), room temperature, 15 min
Cell permeabilization: Triton X-100 (0.5 % in PBS), room temperature, 5 min
Blocking: BSA (5 % in PBS), room temperature, 1 h
Primary antibody (1:100 dilution) incubation: 4 °C, overnight
Secondary antibody (1:2500 dilution) incubation: room temperature, 1 h, dark
DAPI staining: mountant media with DAPI, curing at room temperature, 24 h, dark

Microscopy: Nikon Eclipse Ti2
Objective: 40X/water
Confocal scanning: SoRa mode
DAPI exposure: 80 ms
DyLight 488 exposure: 200 ms
Image processing: Batch Denoise (Denoise.ai – Nikon); NIS-Elements AR Analysis software 5.11.01
Intensity profile feature

ATP5A immunofluorescence visualization

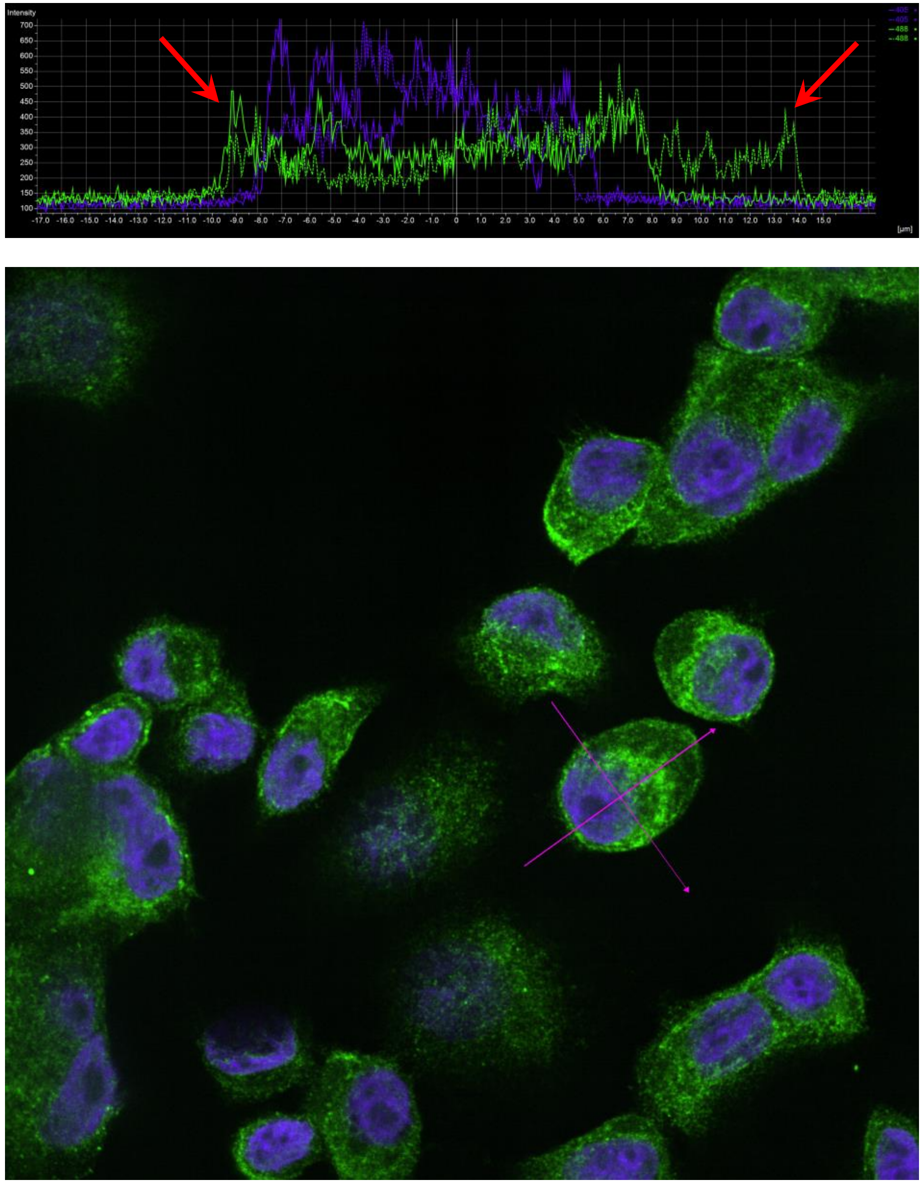

ATP5A_SKBR3_48h arrest

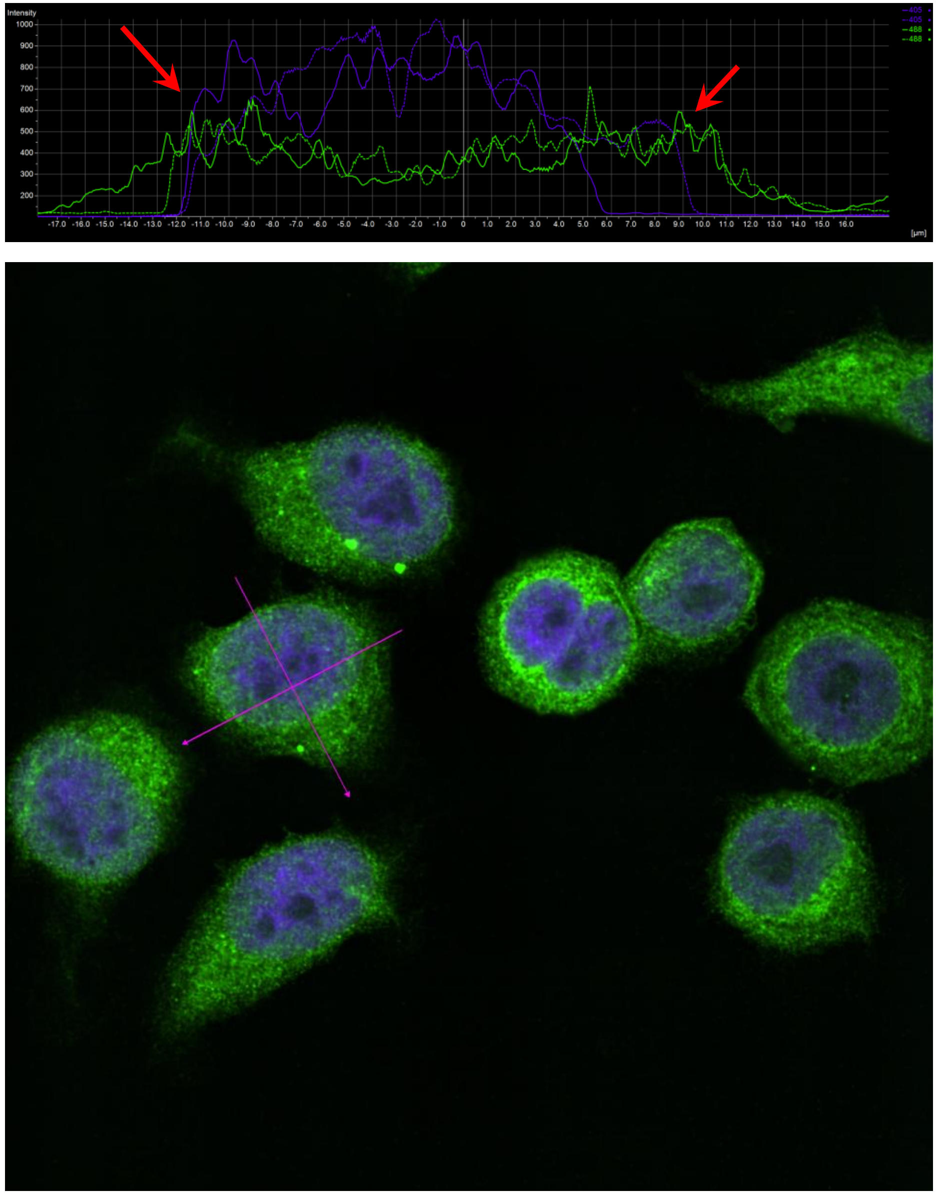

ATP5A_SKBR3_48h arrest/24h release

ATP5FA immunofluorescence visualization (w/o cell permeabilization)

SKBR3 cells
Cell fixation: methanol, -20 °C, 5 min
Cell permeabilization: none
Blocking: BSA (5 % in PBS), room temperature, 1 h
Primary antibody (1:100 dilution) incubation: 4 °C, overnight
Secondary antibody (1:2500 dilution) incubation: room temperature, 1 h, dark
DAPI staining: mountant media with DAPI, curing at room temperature, 24 h, dark

Microscopy: Nikon Eclipse Ti2
Objective: 40X/water
Confocal scanning: SoRa mode
DAPI exposure: 80 ms
DyLight 488 exposure: 200 ms
Image processing: Batch Denoise (Denoise.ai – Nikon); NIS-Elements AR Analysis software 5.11.01

ATP5A immunofluorescence visualization

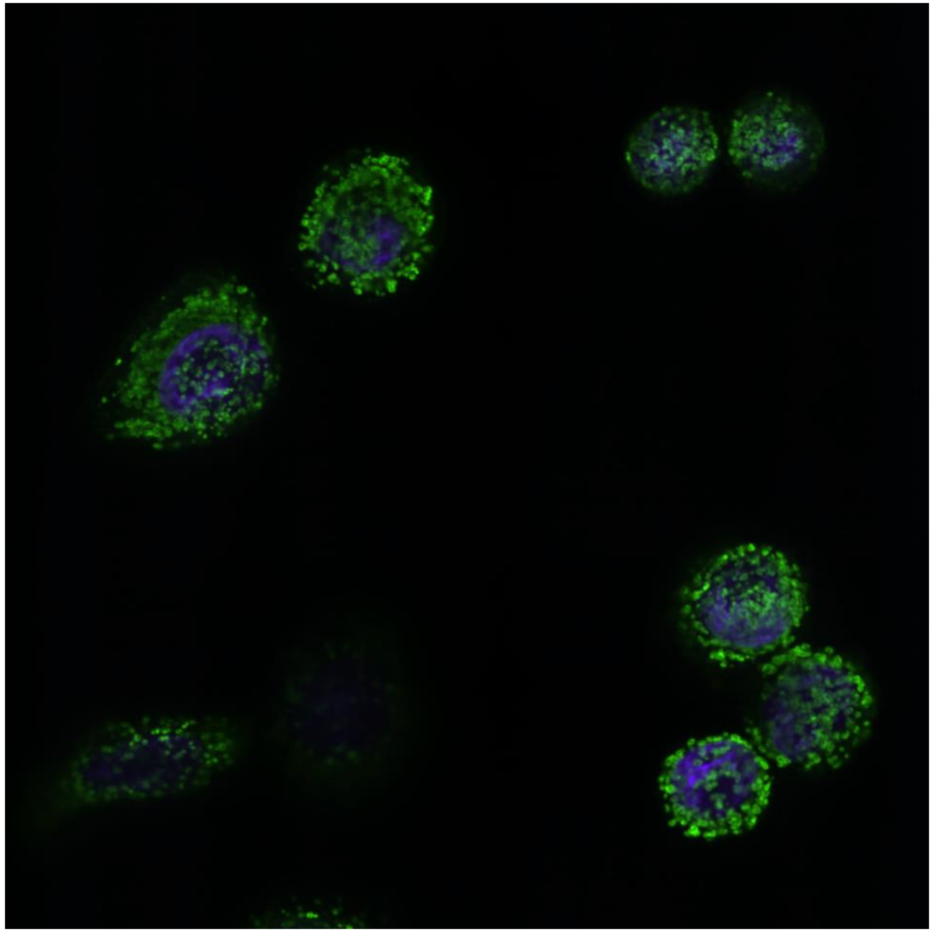

ATP5A_SKBR3_48h arrest

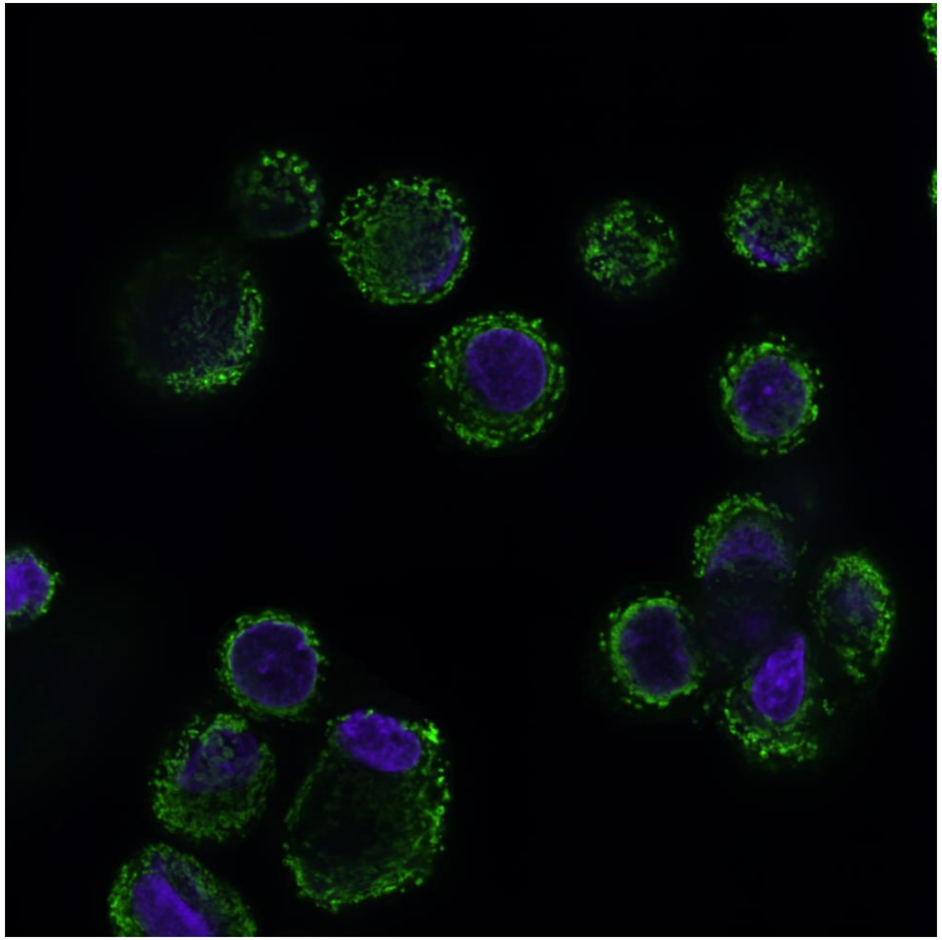

ATP5A_SKBR3_48h arrest/24h release

P2RY2 immunofluorescence visualization

SKBR3 cells
Cell fixation: methanol, -20 °C, 5 min
Cell permeabilization: none
Blocking: BSA (5 % in PBS), room temperature, 1 h
Primary antibody (1:100 dilution) incubation: 4 °C, overnight
Secondary antibody (1:2500 dilution) incubation: room temperature, 1 h, dark
DAPI staining: DAPI solution (1 ug/mL), room temperature, 5 min, dark

Microscopy: Nikon Eclipse TE2000-U
Objective: 20X
DAPI exposure: 10 ms
DyLight 488 exposure: 600 ms
Image processing: NIS-Elements AR Analysis software 5.11.01
Intensity profile feature

P2RY2 immunofluorescence visualization

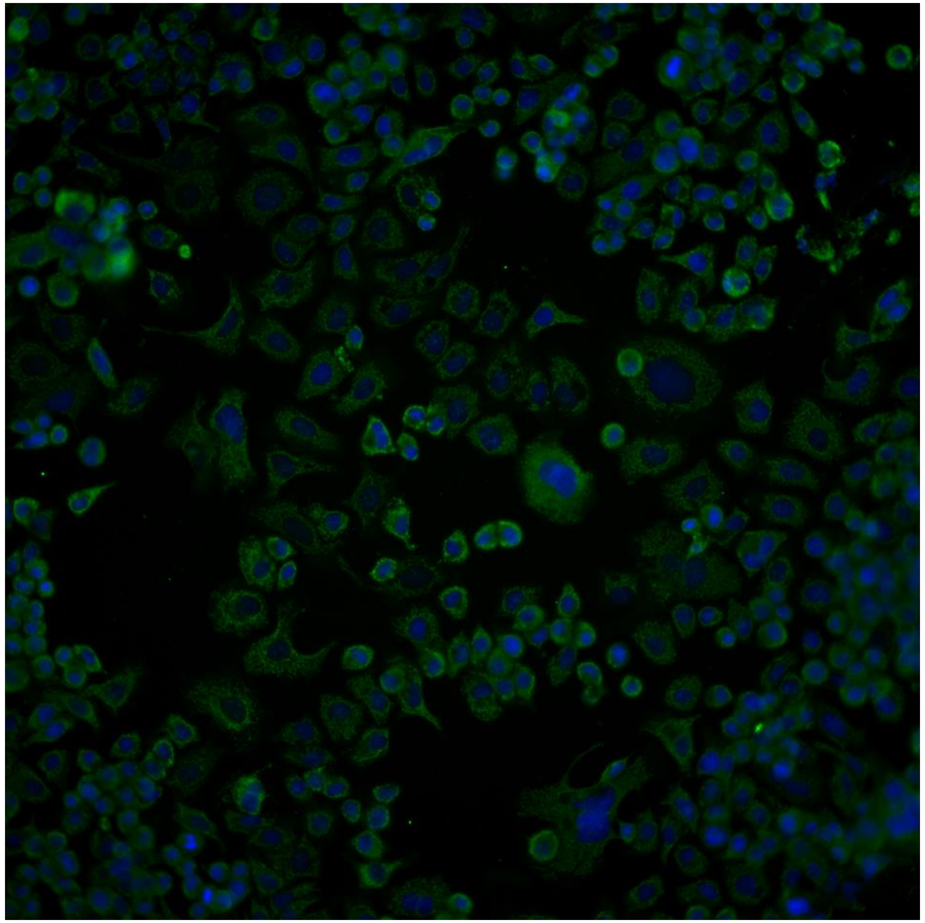

P2RY2_SKBR3_48h arrest

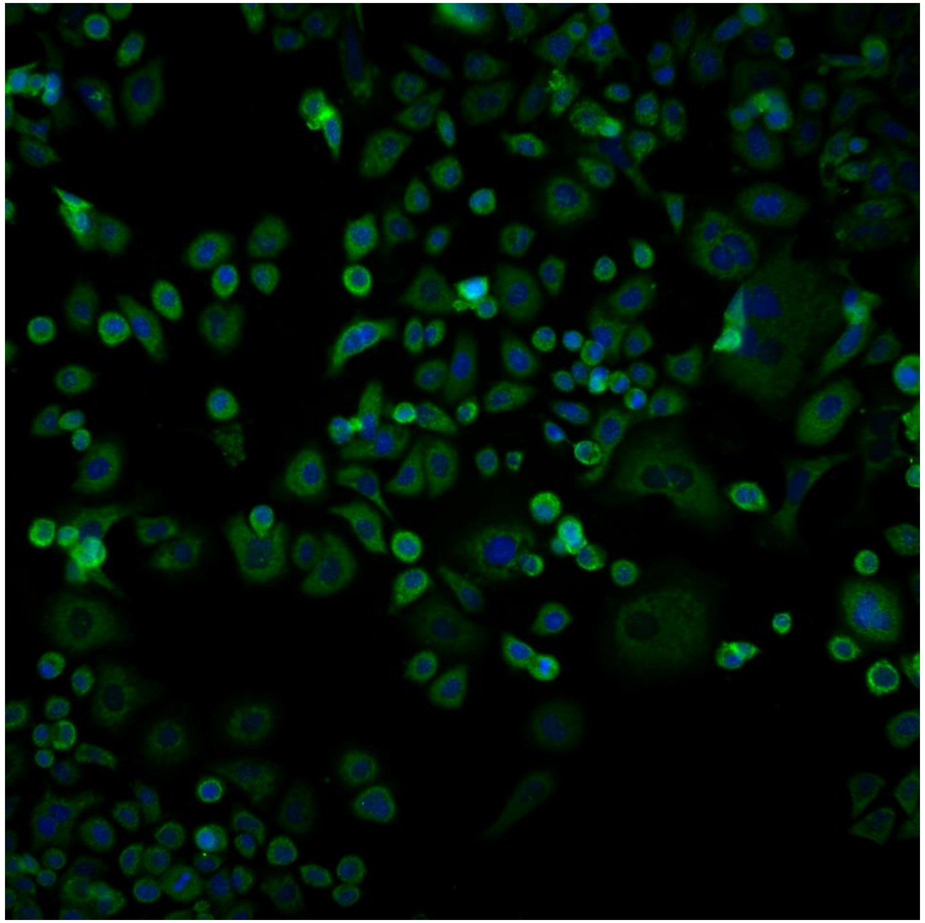

P2RY2_SKBR3_48h arrest/24h release

P2RY2 immunofluorescence visualization (zoom image)

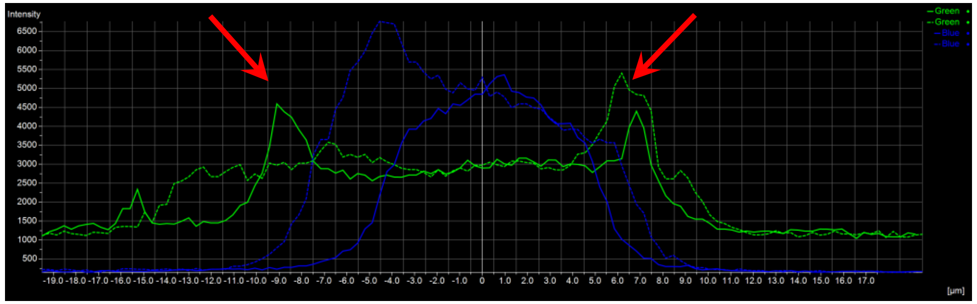

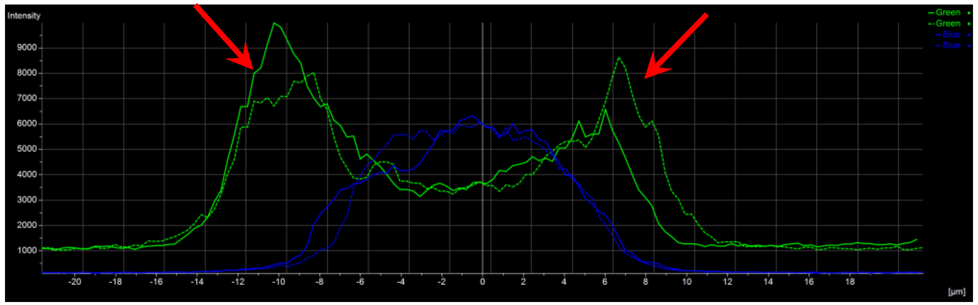

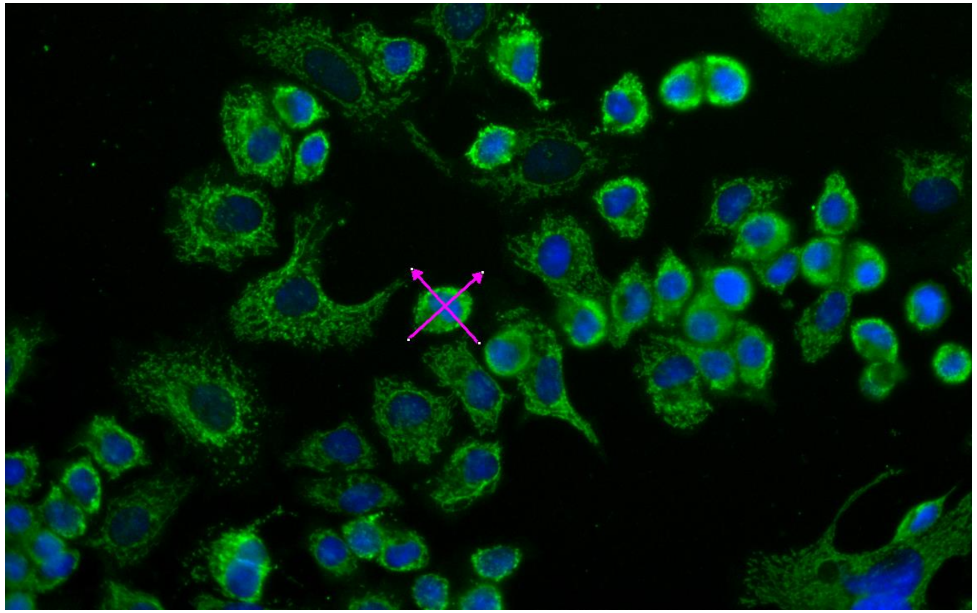

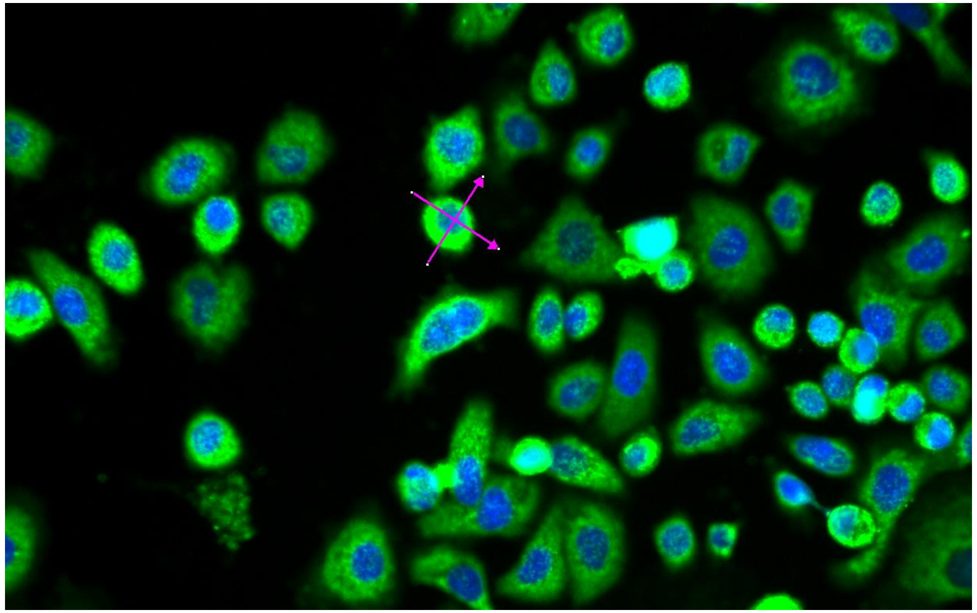

P2RY2_SKBR3_48h arrest

P2RY2_SKBR3_48h arrest/24h release
